# Role of fatty liver index in risk-stratifying comorbid disease outcomes in non-alcoholic fatty liver disease

**DOI:** 10.1016/j.jhepr.2023.100896

**Published:** 2023-08-24

**Authors:** Brian Ho, Andrew Thompson, Andrea L Jorgensen, Munir Pirmohamed

**Affiliations:** 1 Wolfson Centre for Personalised Medicine, Institute of Translational Medicine, University of Liverpool, Liverpool, UK; 2 Department of Pharmacology and Therapeutics, Institute of Translational Medicine, University of Liverpool, Liverpool, UK; 3 Health Analytics, Lane Clark & Peacock LLP, London, UK; 4 Biostatistics, Institute of Translational Medicine, University of Liverpool, Liverpool, UK

**Keywords:** NAFLD, Fatty Liver Index, Comorbidities, Risk Stratification, non-invasive tests

## Abstract

**Background & Aims:**

Population screening for non-alcoholic fatty liver disease (NAFLD) and associated comorbidities remains an unaddressed clinical need. We aimed to assess the utility of the fatty liver index (FLI) for risk stratification of NAFLD and related comorbidities using the UK Biobank.

**Methods:**

Electronic health records and liver MRI-proton density fat fraction (PDFF) were used to define NAFLD cases. FLI was calculated and individuals with high alcohol intake and other liver diseases were excluded. Using listwise deletion analysis, the area under receiver-operating characteristic curve (AUROC) of FLI for NAFLD risk was determined. Thereafter, time-dependent covariate-adjusted Cox regression models were used to estimate FLI’s risk stratification potential for comorbidities of interest.

**Results:**

FLI was derived for 327,800 individuals with a median age of 58 (IQR 51.5-64.5), of whom 59.8% were females. Using Perspectum Diagnostics and AMRA protocols as references, FLI identified the risk of NAFLD with AUROCs (95% CI, n) of 0.858 (0.848-0.867, n = 7,566) and 0.851 (0.844-0.856, n = 10,777), respectively. Intermediate and high-risk FLI was associated with increased cardiometabolic and malignant disease. In the first 3 years, high-risk FLI conferred an increased risk (adjusted hazard ratio, 95% CI) of ischaemic heart disease (2.14, 1.94-2.36), hypertension (2.84, 2.70-2.98), type 2 diabetes mellitus (4.55, 4.04-5.12), dyslipidaemia (2.48, 2.32-2.64), ischaemic stroke (1.31, 1.20-1.42) and hepatic malignancy (1.69, 1.23-2.30). FLI was not associated with risk of extrahepatic malignancy but was associated with a higher risk of specific cancers (colon, upper gastrointestinal and breast). All-cause mortality was similarly stratified by FLI, independently of non-invasive fibrosis scores.

**Conclusions:**

FLI identifies NAFLD and holds potential for the risk stratification of cardiometabolic and malignant disease outcomes (including some extrahepatic malignancies), as well as all-cause mortality. Its use in population screening for primary and secondary prevention of NAFLD should be considered.

**Impact and implications:**

Our analysis using the UK Biobank study shows the potential of the fatty liver index as a risk stratification tool for identifying the risk of developing NAFLD, ischaemic heart disease, ischaemic stroke, type 2 diabetes mellitus, hypertension, hyperlipidaemia, hepatic malignancy, specific metabolism-related malignancies and all-cause mortality. These results suggest that the fatty liver index should be considered as a non-invasive steatosis score that may help guide primary prevention strategies for NAFLD and related outcomes.

## Highlights

• FLI calculated at middle to late age (first attendance to UK Biobank) identifies risk for NAFLD cross-sectionally.
• FLI stratifies incident risk of cardiometabolic diseases, hepatic malignancy, specific metabolism-related malignancies, and all-cause mortality.
• FLI and either NAFLD fibrosis score or FIB4 index independently predict risk of all-cause mortality.
• FLI could be a tool considered for one-off screening of NAFLD and its associated comorbidities.

## Graphical abstract

**Figure:**
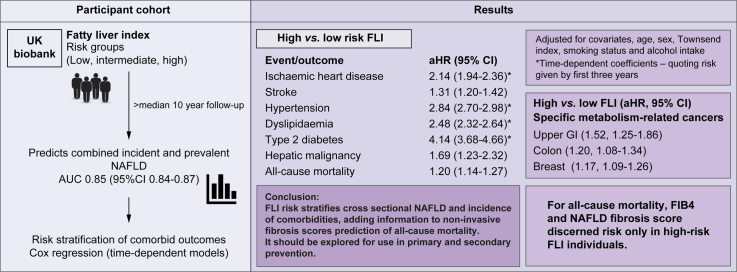

## Introduction

Non-alcoholic fatty liver disease (NAFLD) is estimated to affect 25% of the world’s population and is predicted to become the most prevalent liver disease globally, contributing to healthcare cost and burden.^1^^,^^2^ NAFLD is independently associated with cardiovascular and malignant outcomes, in addition to increasing the risk of progressive liver disease, contributing to morbidity and mortality.[3], [4], [5], [6] As such, NAFLD can be considered as part of a disease syndrome encompassing multiple disease-related comorbidities. This syndrome or “comorbidome” may largely be preventable by addressing metabolic risk factors, but NAFLD-specific primary prevention measures are lacking. In the community, NAFLD is often detected through abnormal liver function tests, such as aminotransferase levels, which are not sensitive and can be normal in advanced disease.^7^^,^^8^ Abdominal ultrasound, often conducted for other reasons, is another modality which NAFLD is identified, but it performs poorly at detecting mild steatosis and is operator-dependent.^9^ As such, identification and clinical stratification of NAFLD risk in the community remains suboptimal, with most individuals with disease being undiagnosed and patient risk factors unaddressed.^10^ This is in contrast to cardiovascular diseases, where primary prevention is widely practiced by addressing risk factors, such as hypertension and dyslipidaemia.

There is interest in identifying chronic liver disease, including NAFLD, at the population level.^11^ To achieve this, automated integrated reflex testing systems have been proposed in the primary care setting – these have been shown to be effective, practical and cost effective.[12], [13], [14] For the identification of NAFLD specifically, a variety of non-invasive clinical tests have also been devised to assess the risk of the presence of steatosis – one such test is the fatty liver index (FLI).^15^ FLI uses BMI, waist circumference, gamma-glutamyltransferase (GGT) and total triglyceride levels for its calculation. The components are simple measures commonly available in primary care and not dependent on co-existent disease.^16^ Since comorbidities of NAFLD can develop before or after the development of liver disease, FLI has been suggested as a promising tool to identify NAFLD-prone individuals and their risk of associated comorbid conditions in primary care, enabling stratification for primary or secondary prevention. However, FLI has not been widely implemented in clinical practice, and indeed there are no national screening programmes to identify individuals with NAFLD, with significant variation in practice between different localities.^17^

In this study, we have utilised the UK Biobank (UKB) to explore the utility of FLI to identify individuals at risk of NAFLD, and its ability to risk stratify incident comorbidities across multiple cardiovascular, metabolic, and malignant outcomes. Further, we mimicked current NAFLD risk stratification strategies by examining the risk of mortality predicted by FLI alone and in combination with two non-invasive fibrosis scores, fibrosis-4 index (FIB4) and NAFLD fibrosis score (NFS).

## Materials and methods

### UK biobank study

The UKB is a prospective study that recruited ∼500,000 individuals aged between 40-69 years in the UK between 2006 and 2010.^18^ The study collected demographic, behavioural, physical, biological sampling, clinical bedside measurement and imaging data from its participants. There is also linked data to national inpatient and primary care records.^19^ Ethics approval was obtained from the UK North-West Multi-Centre Ethics Committee (ref: 16/NW/0274) and informed consent was obtained from each participant for data usage in research purposes. The following data analysis stems from UKB research application ID 54764.

### Study population

FLI was calculated for every individual in the UKB from measurements collected at the initial visit as published by Bedogni *et al.*^16^ All participants with available FLI were included in the study. Individuals with FLI scores of <30, 30-59 and ≥60 were classified as having low, intermediate and high risk of steatosis, respectively. We calculated the weekly alcohol intake of participants as described previously by our group and excluded males and females who drank >21 and 14 weekly standard UK alcohol units, respectively.^20^ Further exclusion criteria included other causes of liver disease, including inherited, viral and alcohol, defined by ICD-9 and ICD-10 codes from linked hospital inpatient data (Tables S1 and S2) or positive serum virology results. NFS and FIB4, two non-invasive fibrosis scores, were also calculated and used to stratify patients into low, intermediate and high-risk groups as previously reported.^21^^,^^22^

### NAFLD case/control definitions

MRI imaging was performed on a Siemens 1.5 Tesla MAGNETOM Aera scanner (Siemens Healthineers, Erlangen, Germany). The analysis protocol for liver MRI-derived proton density fat fraction (PDFF) was developed by two separate companies, Perspectum Diagnostics and AMRA.^23^^,^^24^ At time of data extraction, there were 4,614 and 9,892 participants with available MRI-PDFF data from each company’s protocol, respectively. We arbitrarily derived two case-control cohorts with overlap using each MRI protocol, whereby participants with ≥5% PDFF were defined as having NAFLD. To increase our sample size, ICD9/ICD10 codes and primary care data were used to identify additional NAFLD cases (Fig. S1, Table S3). Healthy individuals were defined by MRI-PDFF <5%.

### Exposure, covariate and outcome variables

Covariates of interest for time-to-event modelling were selected based on previous literature for known associations with NAFLD: age, gender, Townsend deprivation index, smoking status, alcohol intake and type 2 diabetes mellitus (T2DM). We investigated ischaemic heart disease, ischaemic stroke, T2DM, hypertension, hyperlipidaemia, hepatic malignancy, extrahepatic malignancies, and all-cause mortality as events of interest. For extrahepatic malignancies, an additional sub-analysis specific to metabolism-associated cancers, namely colon (including rectal), upper gastrointestinal (GI: oesophageal and stomach) and breast cancer, was performed.Waist-hip ratio and BMI were not used as covariates due to inclusion of BMI and waist circumference in the calculation of FLI. All disease covariates and events of interest were identified through available linked clinical data registries (supplementary methods). The earliest date of recorded disease was considered the time of diagnosis. Exposures of interest are FLI classification for all outcomes and additionally non-invasive fibrosis scores when examining all-cause mortality. Participants who withdrew from the study or died were censored. The analysis was right censored using an arbitrary study end date of 31 December 2019, and the start date for each participant was defined at the UKB’s initial visit date (time of FLI calculation).

### Statistical analysis

The methodology used for statistical analysis is detailed in the supplementary methods. Briefly, descriptive statistics are presented as median and interquartile ranges and proportions for continuous and categorical descriptors, respectively. Performance of FLI in identifying combined incident and prevalent NAFLD was first assessed using receiver-operating characteristic (ROC) analyses, while calculated sensitivities, specificities, positive predictive and negative predictive values are presented for previously published cut-offs of FLI, against defined NAFLD cases and controls. ROC analysis was further performed for two other non-invasive steatosis tests, lipid accumulation product and hepatic steatosis index, calculated as previously published.^25^^,^^26^ Incidence disease rates were calculated for each FLI risk category and for misclassified individuals by FLI for NAFLD. Subsequently, Cox proportional hazard models were fitted to assess incidental disease risk stratification by FLI classification. Univariate models were first fitted to select variables to be included in subsequent multivariate models, with *p* <0.10 used as a loose selection threshold. Model assumptions were tested, and our models were modified to account for any violations of proportional hazards and non-linearity. Two multivariate models were fitted for each outcome. The first model included age and sex, while the second one additionally included all covariates selected from the univariate analysis. Incidental all-cause mortality was investigated in a similar manner through Cox regression modelling. In addition, modelling of FLI and one of the non-invasive fibrosis scores (NFS or FIB4) were investigated with and without adjustment for covariates. In all time-to-event analyses, a listwise deletion dataset was used. *p* values were adjusted by Bonferroni-correction for multiplicity of tests and usefulness of model fitting with FLI and/or fibrosis scores were tested with likelihood ratio tests where appropriate. Additional sensitivity analyses were performed for significance of components of FLI (BMI, waist circumference, triglyceride and GGT levels), and missing covariate data. Analyses was performed on R version 4.0.2 using package pROC, ggplot2, forestplot, survival and survminer.

## Results

### FLI analysis cohort

From the initial UKB cohort of 502,460 participants, 327,800 had all data available for the calculation of FLI after implementing our exclusion criteria. Participant characteristics are described in Table 1. In contrast to individuals in the low-risk FLI, those with higher FLI risk tended to be older, male, smokers, and had higher BMI, waist-hip ratios, as well as greater levels of socioeconomic deprivation. Biochemistry results demonstrated that higher FLI risk was reflective of individuals with increased risk of metabolic and hepatic disease. The median hepatic MRI-PDFF values increased from low- to high-risk FLI.

**Table 1:** Characteristics of patient cohort.

Characteristic	FLI risk						Entire cohort	
	Low (n = 124,126)	n	Intermediate (n = 86,062)	n	High (n = 177,612)	n	Total (n = 327,800)	n
Female	78.62%	97,592	54.03%	46,501	44.17%	51,946	59.80%	196,039
Type 2 diabetes	6.58%	8,162	8.71%	7,495	15.96%	18,765	10.50%	34,422
Smoking status								
Never	66.44%	82,139	61.31%	52,462	54.28%	63,370	60.48%	197,971
Previous	25.72%	31,798	30.14%	25,792	35.94%	41,957	30.41%	99,547
Current	7.84%	9,696	8.55%	7,314	9.79%	11,425	8.69%	28,435
Age (years)	56 (48–62)	124,126	59 (51–64)	86,062	59 (52–64)	117,612	58 (51.5–64.5)	327,800
BMI (kg/h^2^)	23.52 (21.94–25.1)	124,126	26.75 (25.27–28.4)	86,062	31.05 (28.67–34.22)	117,612	26.66 (23.7–29.63)	327,800
Waist-hip ratio	0.47 (0.44–0.49)	124,126	0.53 (0.51–0.55)	86,062	0.6 (0.56–0.64)	117,612	0.53 (0.48–0.58)	327,800
Systolic blood pressure (mmHg)	125 (115–139)	124,075	133 (122–145)	86,016	136 (125–148)	117,372	131 (119–143)	327,463
Diastolic blood pressure (mmHg)	76 (69–82)	124,075	80 (74–86)	86,017	83 (76–89)	117,373	79 (72.5–85.5)	327,465
Alcohol intake (units/week)	7.5 (4–10.5)	88,048	8.2 (4.1–12.1)	60,040	8.6 (4–13.1)	75,490	7.8 (3.8–11.8)	223,578
Townsend deprivation index	–2.33 (–3.75–0.13)	123,980	–2.26 (–3.7–0.33)	85,950	–1.84 (–3.47–1.14)	117,455	–2.15 (–4.24––0.05)	327,385
**Blood parameters**								
Hb (g/dl)	13.6 (12.92–14.3)	120,921	14.2 (13.4–15)	83,839	14.5 (13.62–15.36)	114,417	14.03 (13.2–14.86)	319,177
WBC (x10^9^ cells/L)	6.26 (5.3–7.39)	120,920	6.61 (5.66–7.77)	83,839	7.14 (6.1–8.39)	114,413	6.67 (5.56–7.78)	319,172
Platelet (x10^9^ cells/L)	248.1 (214–286.6)	120,919	249.5 (214.2–288.92)	83,840	250.2 (214.3–291)	114,417	249.2 (211.85–286.55)	319,176
Creatinine (μmol/L)	65.3 (58.4–73.9)	124,109	71.4 (62.1–81.8)	86,046	73.9 (63.9–84.6)	117,602	69.6 (59.9–79.3)	327,757
Total bilirubin	7.92 (6.36–10.21)	123,690	7.91 (6.28–10.28)	85,800	7.8 (6.18–10.12)	117,110	7.87 (5.91–9.83)	326,600
ALT (U/L)	16.01 (13.01–19.98)	124,089	19.86 (15.85–25.29)	86,048	25.2 (19.22–33.99)	117,483	19.59 (13.92–25.26)	327,620
AST (U/L)	22.8 (19.8–26.3)	123,719	24 (20.9–27.9)	85,822	25.7 (22–30.7)	117,068	24.1 (20.35–27.85)	326,609
ALP (U/L)	75.9 (63.1–90.7)	124,092	82.6 (69.8–98)	86,052	86.5 (72.9–102.8)	117,580	81.4 (66.85–95.95)	327,724
GGT (U/L)	17.8 (14.4–23.2)	124,126	24.7 (19.2–33.7)	86,062	35.7 (25.9–53.3)	117,612	24.4 (14.8–34)	327,800
Alb (g/dl)	45.2 (43.52–46.91)	112,878	45.07 (43.4–46.79)	78,753	44.96 (43.22–46.71)	108,369	45.09 (43.38–46.8)	300,000
Urate (μmol/L)	250.9 (214.5–292.7)	123,936	299.3 (256.6–346)	85,923	339.8 (292.4–390.5)	117,467	293.7 (241.2–346.2)	327,326
CRP (mg/L)	0.78 (0.42–1.55)	123,851	1.37 (0.74–2.59)	85,900	2.27 (1.21–4.39)	117,301	1.34 (0.27–2.42)	327,052
HDL-C (mmol/L)	1.6 (1.37–1.85)	112,855	1.35 (1.17–1.57)	78,746	1.18 (1.02–1.38)	108,345	1.37 (1.13–1.62)	299,946
LDL-C (mmol/L)	3.39 (2.88–3.94)	123,936	3.62 (3.05–4.22)	85,931	3.6 (2.95–4.24)	117,415	3.52 (2.93–4.11)	327,282
Triglyceride (mmol/L)	1.05 (0.82–1.36)	124,126	1.54 (1.19–2.02)	86,062	2.13 (1.57–2.9)	117,612	1.48 (0.94–2.02)	327,800
Cholesterol (mmol/L)	5.56 (4.88–6.28)	124,098	5.7 (4.94–6.47)	86,041	5.63 (4.79–6.47)	117,602	5.62 (4.85–6.38)	327,741
**MRI-PDFF quantification (% steatosis)**								
Perspectum Diagnostics algorithm	1.3 (0.97–2)	1,246	2.3 (1.49–4.22)	755	4.21 (2.18–9.32)	790	1.96 (0.51–3.41)	2,791
AMRA Algorithm	1.52 (1.16–2.22)	2,636	2.52 (1.67–4.49)	1,632	4.47 (2.44–9.24)	1,682	2.2 (0.74–3.65)	5,950

Table showing characteristics of UKB participants with data available to calculate FLI and also by the index risk categories. Summary statistics are shown in median and interquartile ranges for continuous values and percentages for categorical values, with the number of available for analysis.

ALP, alkaline phosphatase; ALT, alanine aminotransferase; AST, aspartate aminotransferase; CRP, C-reactive protein; FLI, fatty liver index; GGT, gamma-glutamyltransferase; HDL-C, high-density lipoprotein-cholesterol; LDL-C, low-density lipoprotein-cholesterol; UKB, UK biobank; WBC, white blood cell count.

### Identification of NAFLD risk by FLI

**Fig. 1:**
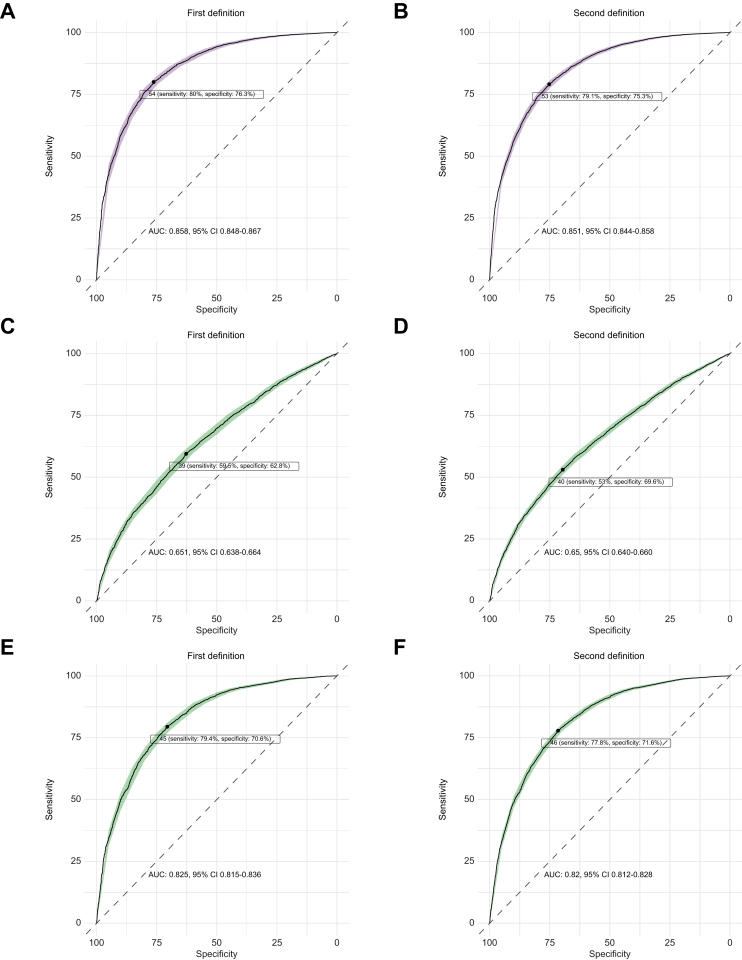
Performance of non-invasive steatosis scores estimating combined prevalent and incident NAFLD risk. Receiver-operating characteristic curves of FLI (A, B), hepatic steatosis index (C, D) and lipid accumulation product (E, F) against two case-control definitions of NAFLD. First definition (left column) and second definition (right column) based on MRI results derived from Perspectum Diagnostics and AMRA, respectively. AUC and best binary cut-off shown, with sensitivity and specificity values. 95% CI were derived from bootstrapping (n = 1,000) and shown in shaded areas. FLI, fatty liver index; NAFLD, non-alcoholic fatty liver disease.

The performance of FLI to identify combined incident and prevalent NAFLD in the UKB was first assessed against two NAFLD case-control definitions, which differ by the proprietary protocol used to measure MRI liver fat fraction. Using the Perspectum Diagnostics’ protocol and clinical health records coding to define NAFLD in the UKB enabled an analysis of 7,656 individuals with available FLI data. Partitioning at a lower FLI cut-off (>30) resulted in a sensitivity, specificity, positive predictive value (PPV) and negative predictive value (NPV) of 93.04%, 53.46%, 75.03% and 83.63%, respectively. Partitioning at the higher cut-off (>60) resulted in a sensitivity, specificity, PPV and NPV of 75.28%, 80.55%, 56.04% and 90.82% respectively (Fig. S2). Similarly, using the alternative MRI protocol derived by AMRA and clinical coding enabled an analysis of 10,777 participants with FLI data. Partitioning at the lower FLI cut-off resulted in a sensitivity, specificity, PPV and NPV of 92.49%, 53.38%, 84.15% and 72.65%, respectively. At the higher cut-off, the values were 73.39%, 80.59%, 69.34% and 83.51%, respectively. On ROC analyses, the AUROC was 0.858 (95% CI 0.848-0.867) and 0.851 (95% CI 0.844-0.856) for these two definitions of NAFLD (Fig. 1). With FLI, 24.3% and 15.6% would be classified as low risk despite having NAFLD, whereas 9.4% and 16.8% would be classified as high risk despite having a normal liver, against our two respective NAFLD definitions. This corresponds to a misclassification rate of 13.3% and 16.4%, when excluding the intermediate-risk group from the calculation. Further comparison was made with two other non-invasive steatosis scores, hepatic steatosis index and lipid accumulation product, which showed inferior ROC results compared to FLI in identifying the risk of NAFLD against these two case-control definitions (Fig. 1).

### Univariate analysis of incident outcomes

**Fig. 2:**
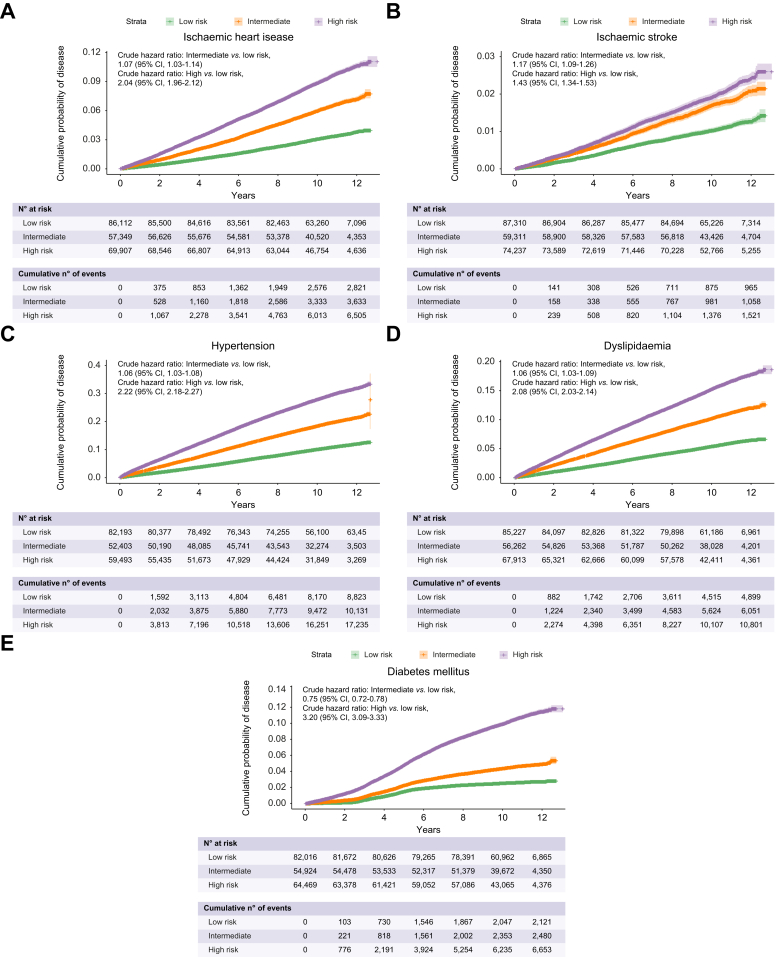
FLI category and cumulative incidence of NAFLD comorbid outcomes. Shown are Kaplan-Meier curves of disease outcomes of interest for low-, intermediate- and high-risk FLI, as defined by scores <30, 30-59 and ≥60, respectively. Incident (A) ischaemic heart disease, (B) ischaemic stroke, (C) hypertension, (D) dyslipidaemia and (E) diabetes mellitus. 95% CIs are plotted (shaded area) with + signs indicating data censoring. Crude hazard ratios are unadjusted derived from univariate Cox regression. FLI, fatty liver index; NAFLD, non-alcoholic fatty liver disease.

**Fig. 3:**
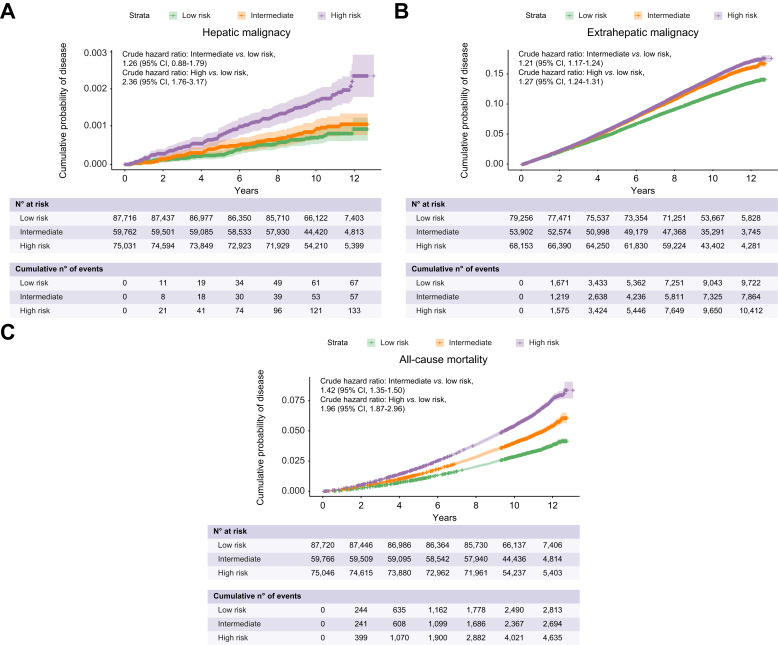
FLI category and cumulative incidence of hepatic and extrahepatic malignancy. Shown are Kaplan-Meier curves for incident (A) hepatic malignancy (B) extrahepatic malignancy and (C) All-cause mortality for low-, intermediate- and high-risk FLI, as defined by scores <30, 30-59 and ≥60, respectively. 95% CIs are plotted for general interpretation (shaded area) with + signs indicating data censoring. Crude hazard ratios are derived from univariate Cox. FLI, fatty liver index.

We next assessed whether FLI and potential covariates may predict the selected comorbid outcomes of NAFLD. The incident rates for low- to high-risk FLI (per 100 person-years) increased from 0.31 to 0.93 for ischaemic heart disease, 0.11 to 0.20 for ischaemic stroke, 1.07 to 3.25 for hypertension, 0.56 to 1.65 for dyslipidaemia, 0.25 to 1.03 for T2DM, 0.01 to 0.02 for hepatic malignancy and 1.22 to 1.56 for extrahepatic malignancy (Fig. 2, Fig. 3, Table S4). Having determined the misclassification rates of FLI, we also calculated the subgroup absolute incidence rates of individuals with NAFLD classified as low-risk FLI and healthy persons with high-risk FLI (Table S5).

Using a listwise deletion approach, over 190,000 individuals were available for univariate and downstream multivariable time-to-event analyses across disease outcomes of interest, with a median follow-up of >10 years (Fig. S3). Univariate analysis showed FLI was associated with all disease outcomes. All covariates selected on univariate analysis also passed the threshold for selection (*p* <0.10) in multivariate models, with some exceptions (Table S6). These were Townsend index (*p* = 0.69) and alcohol intake (*p* = 0.55) for hepatic malignancy and T2DM (*p* = 0.16) for extrahepatic malignancy. Similarly, for all-cause mortality, all covariates passed the selection threshold for multivariable analysis. For FIB-4 and NFS, individuals with intermediate-risk (HR [95% CI] 1.81 [1.74-1.89], *p* = 2.5x10^-^^176^ and 1.98 [1.90-2.06], *p* = 2x10^-^^220^) and high-risk (HR [95% CI] 3.51 [3.19-3.86], *p* = 7.10x10^-^^148^ and 3.77 [3.38-4.19], *p* = 1.4x10^-^^131^) were at significantly increased risk of death, respectively. Furthermore, intermediate and high FLI risk (HR [95% CI] 1.42 [1.35-1.50], *p* = 1.13x10^-38^ and 1.96 [1.87-2.06], *p* = 5.3x10^-^^176^) had increased risk of all-cause mortality compared to low-risk counterparts (Fig. 3 and Fig. S4, Table S7).

### Multivariate analysis of FLI for incident disease outcomes

**Fig. 4:**
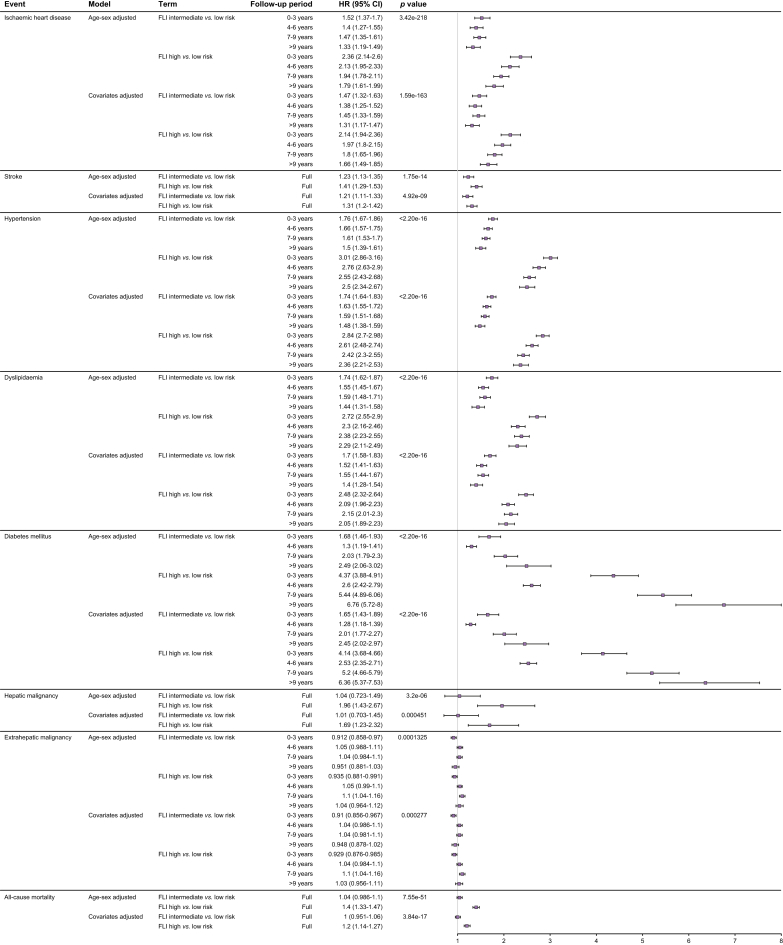
FLI risk stratification of incident comorbidities. Forest plot shows the HR estimates and 95% CIs comparing intermediate- and high-risk FLI with low-risk individuals in multivariable Cox regression analysis, adjusted for covariates selected from univariate regression. The *p* value in the diagram shows log-likelihood ratio test between models with and without FLI as a variable in the regression, assessing the value of FLI in risk prediction. FLI, fatty liver index; HR, hazard ratio.

To assess the ability of FLI to stratify the incidence of selected NAFLD comorbidities, Cox proportional hazards models were fitted with covariates selected from univariate analysis. Of the disease outcomes investigated, FLI met the proportional hazards assumption required for Cox regression on examination of scaled Schoenfeld residuals for ischaemic stroke and hepatic malignancy. Given this, time-dependent coefficient modelling was employed for FLI for all other disease outcomes investigated. For the two outcomes where time-dependent models were not applied, individuals with intermediate-risk FLI had an increased incident risk of ischaemic stroke (HR [95% CI] 1.21 [1.11-1.33], *p* = 2.55x10^-5^), but similar risk of hepatic malignancy (HR [95% CI] 1.01 [0.70-1.45], *p* = 0.965) when compared to those with low-risk FLI. However, high-risk FLI risk-stratified both diseases (HR [95% CI] 1.31 [1.20-1.42], *p* = 1.26x10^-9^ and 1.69 [1.23-2.32], *p* = 0.001, respectively) (Fig. 4).

For time-dependent modelling of FLI, we arbitrarily split the follow-up period into 3-yearly intervals to allow for more refined estimation of risk by FLI during follow-up. Our analysis shows that the incident risk of ischaemic heart disease, hypertension, dyslipidaemia, and T2DM was informed by the non-invasive steatosis score, for both the intermediate- and high-risk category at all intervals of the follow-up period, but not for extrahepatic malignancy (Fig. 4, Table S8). To illustrate, an intermediate-risk FLI was associated with a 1.52-fold [95% CI 1.37-1.70] increased risk of ischaemic heart disease in the first 3 years of follow-up, which reduced to 1.31-fold [95% CI 1.17-1.47] at >9 years of follow-up, when compared to individuals with low-risk FLI. Similarly, high-risk FLI was associated with a 2.14-fold [95% CI 1.94-2.36] and 1.66-fold [95% CI 1.49-1.85] increase in risk when compared to low-risk FLI for these two periods of follow-up, respectively. There was a general trend in the reduction of risk with both intermediate- and high-risk FLI with longer periods of follow-up, except for T2DM. The reduction in estimated risk was most prominent when comparing incident risk of hypertension and dyslipidaemia at up to 3 years with >9 years of follow-up by FLI risk: for intermediate-risk FLI (HR [95% CI] 1.74 [1.64-1.83] *vs.* 1.48 [1.38-1.59] and 1.70 [1.58-1.83 *vs.* 1.40 [1.28-1.54], respectively); and for high-risk FLI (HR [95% CI] 2.84 [2.7-2.98] *vs.* 2.36 [2.21-2.53] and 2.48 [2.32-2.64] *vs.* 2.05 [1.89-2.23], respectively).

In risk-stratifying T2DM, our analysis showed a bimodal pattern of fold-change in the incident risk given by FLI, with higher hazard ratios during the first 3 years and after 6 years of follow-up. When compared to individuals with low-risk FLI (HR, [95% CI]), the intermediate-risk FLI group had 1.83-fold [1.59-2.10], 1.36-fold [1.25-1.48], 2.23-fold [1.97-2.52] and 2.76-fold [2.78-3.34] and the high-risk FLI group had 4.55-fold [4.04-5.12], 2.69-fold [2.50-2.88], 5.69-fold [5.11-6.34] and 7.05-fold [5.95-8.34] higher risk of developing diabetes at 0-3, 4-6, 7-9 and >9 years of follow-up, respectively.

### FLI risk stratification of metabolism-related cancer

**Fig. 5:**
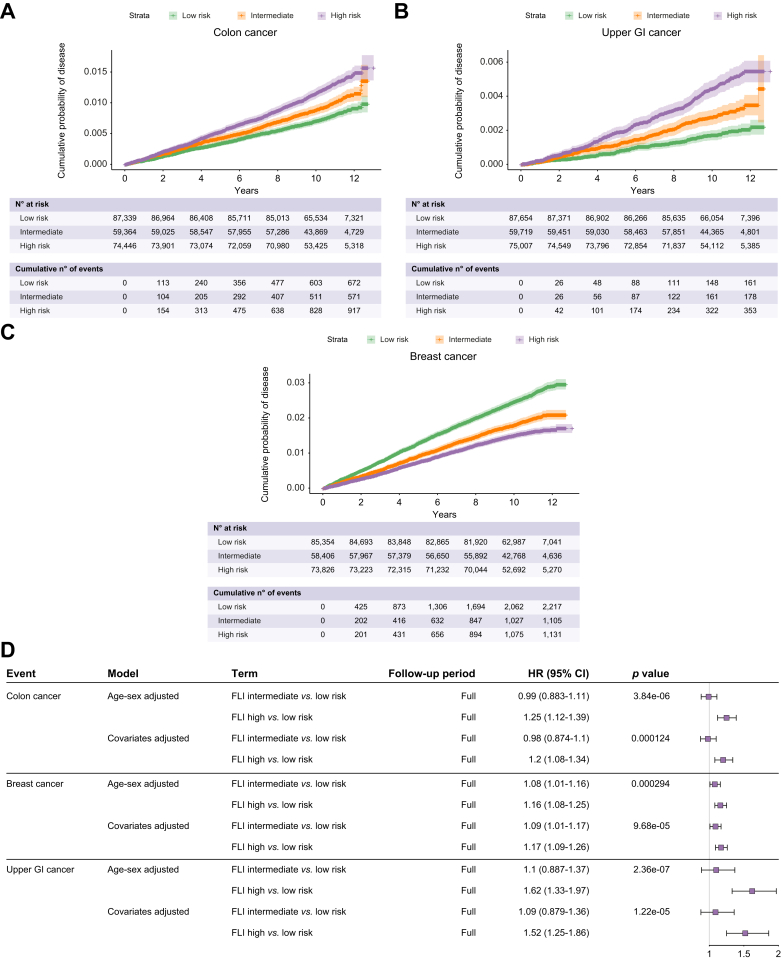
FLI risk stratification of selected incident metabolism-related cancers. Shown are Kaplan-Meier curves for (A) colon, (B) breast and (C) upper GI cancer (oesophageal and gastric) for low-, intermediate- and high-risk FLI, as defined by scores of <30, 30-59 and ≥60. 95% CIs are plotted for general interpretation (shaded area) with + signs indicating data censoring. (D) The forest plot shows the HRs derived from Cox proportional hazards regression adjusted for selected covariates for each of these cancers. FLI, fatty liver index; HR, hazard ratio.

As the value of FLI risk stratification was minimal for overall extrahepatic malignancy, we surmised that non-metabolism-related cancer risk is poorly captured by the components of FLI, which correlate strongly with metabolic disease states, and may therefore dilute estimates of metabolism-related cancers. Additional analysis was therefore performed for selected cancers, which have a known association to metabolic syndrome: colorectal, upper GI (oesophageal and stomach) and breast cancer (Fig. 5). High-risk FLI conferred an increased risk of developing colorectal, upper GI and breast cancer (HR [95% CI] 1.2 [1.08-1.34], 1.52 [1.25-1.86] and 1.17 [1.09-1.26], respectively), when compared to low-risk FLI in the covariate-adjusted model. An intermediate-risk FLI was associated with a 1.09-fold [1.01-1.17] higher risk of breast cancer but not with an increased risk of the luminal GI cancers tested.

### FLI and non-invasive fibrosis scores stratify all-cause mortality

**Fig. 6:**
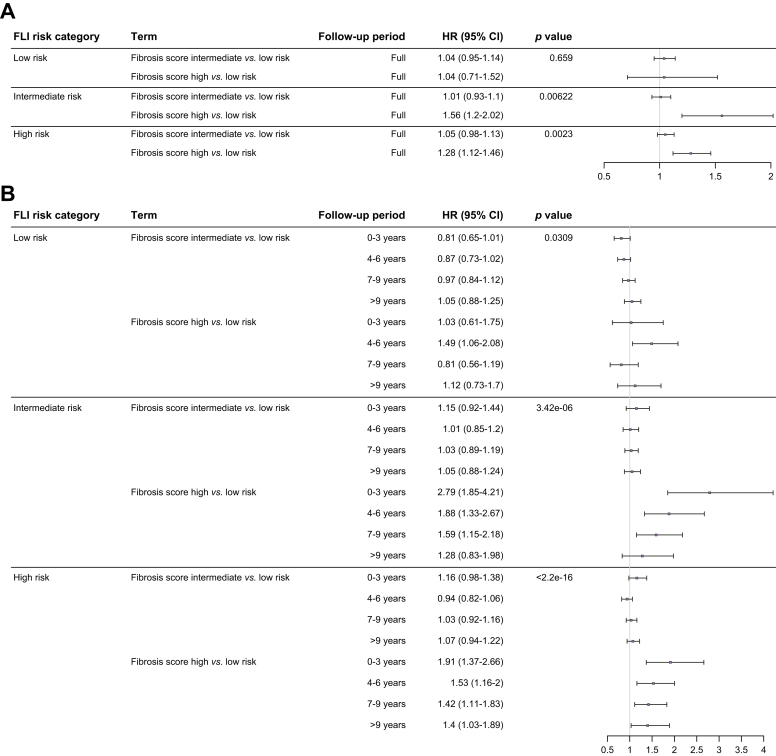
Stratification of mortality with non-invasive fibrosis scores by FLI category. Forest plot shows (A) Fibrosis-4 index and (B) NAFLD fibrosis score category and risk of incident all-cause mortality by FLI risk category. Dot and whiskers show HRs and 95% CIs for adjusted Cox regression adjusted for covariates: age, sex, Townsend index, type 2 diabetes mellitus, smoking status, and alcohol intake. *p* value indicates likelihood ratio test comparing models with and without non-invasive fibrosis score as a regressor, assessing their value in predicting mortality in addition to FLI. FLI, fatty liver index; HR, hazard ratio.

Finally, all-cause mortality was examined in a similar manner. In the covariate-adjusted model, high-risk FLI was associated with a 1.2-fold [95% CI 1.14-1.27] increased risk of death from any cause, whereas individuals with intermediate-risk scores had a similar risk of death as those with low-risk FLI (Fig. 4). As current advanced liver disease risk assessment involves confirming the presence of fibrosis, which is independently associated with mortality, we examined whether FLI provides any independent information over non-invasive fibrosis scores (NFS or FIB4), a surrogate to the presence of fibrotic liver disease. To do this, we first fitted separate stratified Cox models by FLI risk category, which showed that high-risk NFS and FIB4 predicted an increased risk of all-cause mortality in individuals with intermediate- or high-risk, but not low-risk, FLI (Fig. 6 and Fig. S7). Incorporation of FLI with each non-invasive fibrosis score into a unified covariate-adjusted model showed that only the high-risk category of each non-invasive fibrosis score was significantly associated with a higher risk of death from all-causes. In each of these models, individuals with a high-risk FLI had a 1.2-fold [95% CI: 1.13 - 1.26] and 1.22-fold [1.16-1.28] higher risk of all-cause mortality compared to individuals with low-risk FLI when adjusted for NFS and FIB4, respectively (Table 2 and Table S9).

**Table 2:** Risk stratification of all-cause mortality when combining FLI and non-invasive fibrosis scores.

Cox regression model	Follow-up period	Fibrosis score (intermediate *vs.* low risk)		Fibrosis score (high *vs.* low risk)		FLI (intermediate *vs.* low risk)		FLI (high *vs.* low risk)	
		HR (95% CI)	*p* value	HR (95% CI)	*p* value	HR (95% CI)	*p* value	HR (95% CI)	*p* value
FLI + FIB4	0-3 years	1.80 (1.61–2.00)	3.83E^-27^	4.03 (3.23–5.04)	1.80E^-34^	1.42 (1.35–1.50)	1.21E^-37^	2.03 (1.93–2.13)	4.44E^-187^
	4-6 years	1.68 (1.55–1.83)	1.95E^-36^	3.78 (3.18–4.50)	8.32E^-51^	–	–	–	–
	7-9 years	1.89 (1.76–2.03)	8.13E^-71^	3.13 (2.64–3.71)	8.23E^-40^	–	–	–	–
	>9 years	2.06 (1.90–2.23)	1.25E^-66^	3.33 (2.70–4.11)	2.37E^-29^	–	–	–	–
FLI + NFS	0-3 years	1.68 (1.51–1.88)	9.74E^-21^	3.39 (2.63–4.37)	3.39E^-21^	1.33 (1.26–1.41)	1.72E^-23^	1.68 (1.60–1.77)	8.46E^-90^
	4-6 years	1.71 (1.57–1.86)	2.52E^-36^	2.89 (2.34–3.57)	5.40E^-23^	–	–	–	–
	7-9 years	1.83 (1.70–1.97)	4.92E^-60^	3.21 (2.69–3.84)	1.68E^-37^	–	–	–	–
	>9 years	2.00 (1.84–2.18)	3.25E^-58^	3.23 (2.55–4.08)	1.17E^-22^	–	–	–	–
FLI + FIB4 + Cov	0-3 years	1.06 (0.94–1.18)	0.36	1.85 (1.46–2.33)	2.08E^-07^	1.01 (0.95–1.07)	0.76	1.22 (1.16–1.28)	6.71E^-14^
	4-6 years	0.94 (0.86–1.03)	0.16	1.61 (1.34–1.93)	2.37E^-07^	–	–	–	–
	7-9 years	1.01 (0.94–1.09)	0.74	1.26 (1.06–1.50)	0.010	–	–	–	–
	>9 years	1.05 (0.96–1.15)	0.27	1.27 (1.03–1.58)	0.027	–	–	–	–
FLI + NFS + Cov	0-3 years	1.04 (0.93–1.16)	0.52	1.60 (1.23–2.07)	0.0004	1.00 (0.94–1.06)	0.97	1.20 (1.13–1.26)	8.3E^-11^
	4-6 years	1.01 (0.92–1.10)	0.85	1.24 (1.00–1.53)	0.052	–	–	–	–
	7-9 years	1.03 (0.95–1.11)	0.47	1.27 (1.05–1.52)	0.012	–	–	–	–
	>9 years	1.05 (0.97–1.15)	0.24	1.15 (0.90–1.46)	0.263	–	–	–	–

Table shows time-dependent Cox regression results of FLI and one non-invasive fibrosis scores (FIB4 or NAFLD fibrosis score), adjusted and not adjusted for covariates, which include age, sex, Townsend index, type 2 diabetes mellitus, smoking status, and alcohol intake.

Cov, covariates; FIB4, fibrosis-4 index; FLI, fatty liver index; HR, hazard ratio; NFS, NAFLD fibrosis score.

### Sensitivity analysis for components of FLI and missing data

Given the strong risk differences in FLI risk groups for the disease outcomes investigated, sensitivity analyses were performed to investigate whether components of FLI vary in their risk predictive capacity for these different outcomes. Using multivariate analysis to determine the role of the individual FLI components showed that ischaemic heart disease, T2DM, hypertension, dyslipidaemia and extrahepatic malignancy were informed by all components of FLI. The only outcome associated with three FLI components was all-cause mortality (waist circumference, BMI and GGT levels). Hepatic malignancy and ischaemic stroke were only significantly associated with two components of FLI (Table S10). Of note, waist circumference and BMI exhibited opposite effects towards the risk of extrahepatic malignancy. A further sensitivity analysis was performed for the main outcome analysis to investigate effects of missing data when using listwise deletion. To crudely investigate this, we ran the same time-to-event regression analysis for disease outcomes of interest with a listwise deletion cohort excluding alcohol intake as a variable, which had the largest proportion of missingness (of up to 20%). Comparing it with the main analysis cohort, little difference in risk estimates was shown for FLI, whereby estimates of risk were consistently more conservative in the final models used (Table S11).

## Discussion

In one of the largest studies undertaken to date, we have demonstrated that a higher FLI from a single measurement performed in the UKB identified individuals at risk of existent and incident NAFLD and its comorbidities. A previous meta-analysis of 27,221 individuals showed that the sensitivity, specificity, PPV, and NPV for FLI were 81%, 65%, 53%, and 84%, respectively, for the lower cut-off, while the corresponding values at the higher cut-off were 44%, 90%, 67%, and 76%, respectively.^27^ Our data compare favourably with these estimates, but with slightly higher sensitivity values and slightly lower specificity at both cut-off thresholds. A previous study comparing FLI with ^1^H-magnetic resonance spectroscopy showed an AUROC of 0.79 compared with our value of 0.85, suggesting FLI has good diagnostic value.^28^ However, in contrast to its use for point of care diagnosis in the previous studies, our results suggest that FLI may be capable of identifying NAFLD and associated comorbidities over a lifetime period up to middle and early old-age. This is reflected by the age group recruited in the UKB and reflects risk stratification of a one-off score between the ages of 40-69.

A misclassification rate of FLI for NAFLD is also observed from our analysis. This is expected given only a small proportion of risk is captured by the index for the complex trait disorder. As such, it is important to distinguish that our time-to-event analysis results do not reflect a direct assessment of NAFLD towards the risk of developing the disease, but rather the risk from metabolic factors associated with NAFLD development, which it shares with many other metabolic conditions. FLI thus provides a validated score to link NAFLD with incident disease outcome assessment, which has not been considered in the development of many established risk stratification tools in use, such as QRISK3 for cardiovascular disease.^29^ Misclassification may hamper the prediction capacity of disease outcomes, but from our results, there were generally comparable comorbid event rates in NAFLD cases with a low-risk FLI and those with healthy livers and a high-risk FLI.

Bearing this in mind, our results show that the risk of ischaemic heart disease, stroke, diabetes, hypertension, hyperlipidaemia, hepatic malignancy, and all-cause mortality was stratified by FLI longitudinally over a median follow-up >10 years, after adjustment of known covariates associated with NAFLD development. Several studies have shown that FLI can predict the risk of cardiometabolic disease and overall mortality, which is consistent with our finding.[30], [31], [32], [33], [34] However, we have also shown that FLI predicts of the risk of hepatic and certain extrahepatic malignancies as well, which is a novel finding. A previous study has examined FLI stratification of cardiac events and stroke within the UKB, in which the authors used FLI deciles for modelling.^35^ This may hamper ease of clinical interpretation given the index was originally designed as a tripartite classification system (low, intermediate and high risk for NAFLD). The presented results show a similar direction of the estimated effect, with our use of time-dependent models potentially providing more precise risk estimates in an updated analysis with a longer follow-up period. Separately, FLI has also been shown to risk stratify incident hypertension and dyslipidaemia in comparatively smaller Asian cohorts.^31^^,^^36^ Our results therefore provide further information on FLI’s utility for risk stratification, specifically in a UK cohort composed mainly of Caucasians.

We show that a high-risk FLI (>60) was predictive of a higher risk of hepatic malignancy when compared to an index score of <30. This conforms well with the current understanding that NAFLD-related metabolic risk factors increase the risk of disease progression. However, there was no overall risk prediction of incident extrahepatic malignancy, which is contrary to the association of NAFLD with the development of and mortality from extrahepatic malignancy.^37^^,^^38^ The discrepancy is likely related to differences in the outcome investigated, whereby cancer-related mortality is more strongly predicted by metabolic comorbidities reflected by FLI but potentially less so with incidence of all types of cancer. Supporting this, in a recent study investigating incident cancer and cancer-related mortality, using raised alanine aminotransferase levels as a surrogate marker for NAFLD in three independent Scottish cohorts, it was shown that FLI predicted risk of mortality from cancer.^39^ However, the differences in modelling and covariates with the present study could also influence these results. In other studies, FLI was shown to be associated with risk of colorectal, pancreatic and breast cancer development in a Korean population.[40], [41], [42] We also examined specific incidences of metabolism-related malignancies, namely colorectal, breast and upper GI cancers (oesophageal and gastric), and show that high-risk FLI was able to identify increased risk relative to low-risk scoring individuals, independent of common risk factors for NAFLD.

Lastly, we demonstrate that high-risk FLI was also associated with increased all-cause mortality independent of age, sex, diabetes, alcohol intake and socioeconomic deprivation, consistent with NAFLD and metabolic risk factors being predictive factors. To mimic the current two-step strategy of clinical NAFLD assessment which first stratifies risk of disease and then subsequently risk of advance disease, we explored whether FLI combined with non-invasive fibrosis scores can be used to assess mortality risk. Results show that non-invasive fibrosis scores and FLI independently stratified risk of all-cause mortality. Furthermore, in individuals with intermediate- and high-risk FLI, both fibrosis scores predicted all-cause mortality in their high-risk category in our adjusted model. This suggests FLI adds information to risk assessment of all-cause mortality and can potentially be used in tandem with fibrosis scores for this purpose.

Our study has certain limitations. The use of a listwise deletion cohort may have created bias, but our sensitivity analysis suggests our estimates were relatively precise when discounting for the largest missing variable (alcohol intake). Given the observational nature of the UKB, FLI’s use in the general population and in individuals with NAFLD as a means of follow-up should be directly assessed in future intervention studies. Lastly, cost-effectiveness analysis will be required to build a case for implementation, which is beyond the scope of our study. With different components of FLI showing different directions of risk in certain disease outcomes assessed, further refinement of the index for specific outcomes may be attractive, but this must be balanced against the fact that diversification of risk stratification tools may increase complexity and thereby difficulty in clinical implementation.

Overall our results suggest that the FLI is an attractive potential option to identify individuals at risk of NAFLD and related comorbidities within the community, particularly during routine follow-up for other metabolic diseases in primary care. Whether the degree of misclassification we have identified is clinically acceptable warrants further debate, but it is probably better than the current standard of care which varies with geography and mostly amounts to doing nothing.^17^ Clearly, as we have observed, the risk effect estimates observed from a single FLI may change over time, and thus repeated FLI measurements may provide better indication of comorbid disease and mortality risk, as shown by a previous study.^43^

In summary, the FLI identified prevalent and incident NAFLD and stratified the risk of incident cardiovascular and metabolic diseases, hepatic malignancy, and some extrahepatic cancers. FLI alone or in combination with NFS or FIB-4 independently enabled the risk assessment of all-cause mortality. FLI shows potential as a tool to stratify individuals for further assessment and to guide the development of prevention strategies for NAFLD and its related comorbidities in the population.

## Financial support

BH is an MRC Clinical Training Fellow based at the 10.13039/501100000836University of Liverpool supported by the North West England 10.13039/501100000265Medical Research Council Fellowship Scheme in Clinical Pharmacology and Therapeutics, which is funded by the 10.13039/501100000265Medical Research Council (Award Ref. MR/N025989/1), Roche Pharma, Eli Lilly and Company Limited, UCB Pharma, Novartis, the University of Liverpool and the University of Manchester.

## Authors’ contributions

Funding: MP, Manuscript concept: BH, AT and MP. Manuscript design and writing: BH. Statistical analysis: BH and AJ. Revision, editing and acceptance of final version: all authors.

## Data availability statement

The data used for analysis is obtained from the UK Biobank and processes of obtaining this is stated in http://www.ukbiobank.ac.uk/using-the-resource/.

## Conflicts of interest

M.P. has received partnership funding (to the University of Liverpool) for the following: MRC Clinical Pharmacology Training Scheme (co-funded by MRC and Roche, UCB, Eli Lilly and Novartis; this scheme was used to fund BH for this work); a PhD studentship jointly funded by EPSRC and Astra Zeneca; and grant funding from Vistagen Therapeutics. He has also unrestricted educational grant support for the UK Pharmacogenetics and Stratified Medicine Network from Bristol-Myers Squibb. He has developed an HLA genotyping panel with MC Diagnostics, but does not benefit financially from this. He is part of the IMI Consortium ARDAT (www.ardat.org). Other investigators have no conflicts of interest to declare.

Please refer to the accompanying ICMJE disclosure forms for further details.

## References

[1] Younossi Z.M., Koenig A.B., Abdelatif D., Fazel Y., Henry L., Wymer M. (2016). Global epidemiology of nonalcoholic fatty liver disease—meta-analytic assessment of prevalence, incidence, and outcomes. Hepatology.

[2] Adejumo A.C., Samuel G.O., Adegbala O.M., Adejumo K.L., Ojelabi O., Akanbi O. (2019). Prevalence, trends, outcomes, and disparities in hospitalizations for nonalcoholic fatty liver disease in the United States. Ann Gastroenterol.

[3] Ekstedt M., Franzén L.E., Mathiesen U.L., Thorelius L., Holmqvist M., Bodemar G. (2006). Long-term follow-up of patients with NAFLD and elevated liver enzymes. Hepatology.

[4] Alexander M., Loomis A.K., Van DerLei J., Duarte-Salles T., Prieto-Alhambra D., Ansell D. (2019). Non-alcoholic fatty liver disease and risk of incident acute myocardial infarction and stroke: findings from matched cohort study of 18 million European adults. BMJ.

[5] Alexander M., Loomis A.K., Van DerLei J., Duarte-Salles T., Prieto-Alhambra D., Ansell D. (2019). Risks and clinical predictors of cirrhosis and hepatocellular carcinoma diagnoses in adults with diagnosed NAFLD: real-world study of 18 million patients in four European cohorts. BMC Med.

[6] Huang D.Q., El-Serag H.B., Loomba R. (2020). Global epidemiology of NAFLD-related HCC: trends, predictions, risk factors and prevention. Nat Rev Gastroenterol Hepatol.

[7] Mofrad P., Contos M.J., Haque M., Sargeant C., Fisher R.A., Luketic V.A. (2003). Clinical and histologic spectrum of nonalcoholic fatty liver disease associated with normal ALT values. Hepatology.

[8] Browning J.D., Szczepaniak L.S., Dobbins R., Nuremberg P., Horton J.D., Cohen J.C. (2004). Prevalence of hepatic steatosis in an urban population in the United States: impact of ethnicity. Hepatology.

[9] Hernaez R., Lazo M., Bonekamp S., Kamel I., Brancati F.L., Guallar E. (2011). Diagnostic accuracy and reliability of ultrasonography for the detection of fatty liver: a meta-analysis. Hepatology.

[10] Alexander M., Loomis A.K., Fairburn-Beech J., van derLei J., Duarte-Salles T., Prieto-Alhambra D. (2018). Real-world data reveal a diagnostic gap in non-alcoholic fatty liver disease. BMC Med.

[11] Yeoman A.D. (2021). Novel approaches to detect significant liver disease in the general population. Clin Liver Dis.

[12] Chalmers J., Wilkes E., Harris R., Kent L., Kinra S., Aithal G. (2020). Original research: development and implementation of a commissioned pathway for the identification and stratification of liver disease in the community. Frontline Gastroenterol.

[13] Dillon J.F., Miller M.H., Robinson E.M., Hapca A., Rezaeihemami M., Weatherburn C. (2019). Intelligent liver function testing (iLFT): a trial of automated diagnosis and staging of liver disease in primary care. J Hepatol.

[14] Srivastava A., Gailer R., Tanwar S., Trembling P., Parkes J., Rodger A. (2019). Prospective evaluation of a primary care referral pathway for patients with non-alcoholic fatty liver disease. J Hepatol.

[15] Wong V.W.S., Adams L.A., deLédinghen V., Wong G.L.H., Sookoian S. (2018). Noninvasive biomarkers in NAFLD and NASH — current progress and future promise. Nat Rev Gastroenterol Hepatol.

[16] Bedogni G., Bellentani S., Miglioli L., Masutti F., Passalacqua M., Castiglione A. (2006). The fatty liver index: a simple and accurate predictor of hepatic steatosis in the general population. BMC Gastroenterol.

[17] Neilson L.J., Macdougall L., Lee P.S., Hardy T., Beaton D., Chandrapalan S. (2021). Implementation of a care bundle improves the management of patients with non-alcoholic fatty liver disease. Frontline Gastroenterol.

[18] Sudlow C., Gallacher J., Allen N., Beral V., Burton P., Danesh J. (2015). UK biobank: an open access resource for identifying the causes of a wide range of complex diseases of middle and old age. PLOS Med.

[19] Littlejohns, T. J., Holliday, J., Gibson, L. M., Garratt, S., Oesingmann, N., Alfaro-Almagro, F., et al. The UK Biobank imaging enhancement of 100,000 participants: rationale, data collection, management and future directions. doi:10.1038/s41467-020-15948-9. 10.1038/s41467-020-15948-9 PMC7250878 32457287

[20] Thompson A., Cook J., Choquet H., Jorgenson E., Yin J., Kinnunen T. (2020). Functional validity, role, and implications of heavy alcohol consumption genetic loci. Sci Adv.

[21] Angulo P., Hui J.M., Marchesini G., Bugianesi E., George J., Farrell G.C. (2007). The NAFLD fibrosis score: a noninvasive system that identifies liver fibrosis in patients with NAFLD. Hepatology.

[22] McPherson S., Stewart S.F., Henderson E., Burt A.D., Day C.P. (2010). Simple non-invasive fibrosis scoring systems can reliably exclude advanced fibrosis in patients with non-alcoholic fatty liver disease. Gut.

[23] Linge J., Borga M., West J., Tuthill T., Miller M.R., Dumitriu A. (2018). Body composition profiling in the UK biobank imaging study. Obesity.

[24] Wilman H.R., Kelly M., Garratt S., Matthews P.M., Milanesi M., Herlihy A. (2017). Characterisation of liver fat in the UK Biobank cohort. PLoS One.

[25] Cuthbertson D.J., Weickert M.O., Lythgoe D., Sprung V.S., Dobson R., Shoajee-Moradie F. (2014). External validation of the fatty liver index and lipid accumulation product indices, using 1H-magnetic resonance spectroscopy, to identify hepatic steatosis in healthy controls and obese, insulin-resistant individuals. Eur J Endocrinol.

[26] Lee J.H., Kim D., Kim H.J., Lee C.H., Yang J.I., Kim W. (2010). Hepatic steatosis index: a simple screening tool reflecting nonalcoholic fatty liver disease. Dig Liver Dis.

[27] Castellana M., Donghia R., Guerra V., Procino F., Lampignano L., Castellana F. (2021). Performance of fatty liver index in identifying non-alcoholic fatty liver disease in population studies. A meta-analysis. J Clin Med.

[28] Cuthbertson D.J., Koskinen J., Brown E., Magnussen C.G., Hutri-Kähönen N., Sabin M. (2021). Fatty liver index predicts incident risk of prediabetes, type 2 diabetes and non-alcoholic fatty liver disease (NAFLD). Ann Med.

[29] Hippisley-Cox J., Coupland C., Brindle P. (2017). Development and validation of QRISK3 risk prediction algorithms to estimate future risk of cardiovascular disease: prospective cohort study. BMJ.

[30] Franch-Nadal J., Caballeria L., Mata-Cases M., Mauricio D., Giraldez-García C., Mancera J. (2018). Fatty liver index is a predictor of incident diabetes in patients with prediabetes: the PREDAPS study. PLoS One.

[31] Huh J.H., Ahn S.V., Koh S.B., Choi E., Kim J.Y., Sung K.-C. (2015). A prospective study of fatty liver index and incident hypertension: the KoGES-ARIRANG study. PLoS One.

[32] Khang A.R., Lee H.W., Yi D., Kang Y.H., Son S.M. (2019). The fatty liver index, a simple and useful predictor of metabolic syndrome: analysis of the Korea National Health and Nutrition Examination Survey 2010–2011. Diabetes Metab Syndr Obes Targets Ther.

[33] Kim J.Y.J.H., Moon J.S., Byun S.J., Lee J.H., Kang D.R., Sung K.C. (2020). Fatty liver index and development of cardiovascular disease in Koreans without pre-existing myocardial infarction and ischemic stroke: a large population-based study. Cardiovasc Diabetol.

[34] Yadav D., Choi E., Ahn S.V., Koh S.B., Sung K.C., Kim J.Y. (2016). Fatty liver index as a simple predictor of incident diabetes from the KoGES-ARIRANG study. Med (United States).

[35] Zou B., Yeo Y.H., Cheung R., Ingelsson E., Nguyen M.H. (2021). Fatty liver index and development of cardiovascular disease: findings from the UK biobank. Dig Dis Sci.

[36] Higashiura Y., Furuhashi M., Tanaka M., Takahashi S., Mori K., Miyamori D. (2021). Elevated fatty liver index is independently associated with new onset of hypertension during a 10-year period in both male and female subjects. J Am Hear Assoc Cardiovasc Cerebrovasc Dis.

[37] Musso G., Gambino R., Cassader M., Pagano G. (2011). Meta-analysis: natural history of non-alcoholic fatty liver disease (NAFLD) and diagnostic accuracy of non-invasive tests for liver disease severity. Ann Med.

[38] Liu Y., Zhong G.C., Tan H.Y., Hao F.B., Hu J.J. (2019). Nonalcoholic fatty liver disease and mortality from all causes, cardiovascular disease, and cancer: a meta-analysis. Sci Rep.

[39] Taylor A., Siddiqui M.K., Ambery P., Armisen J., Challis B.G., Haefliger C. (2022). Metabolic dysfunction-related liver disease as a risk factor for cancer. BMJ Open Gastroenterol.

[40] Park J.H., Hong J.Y., Han K., Kang W., Park J.K. (2022). Increased risk of pancreatic cancer in individuals with non-alcoholic fatty liver disease. Sci Rep.

[41] Park J.H., Choi I.S., Han K.Do, Park H., Kim K.H., Kim J.S. (2020). Association between fatty liver index and risk of breast cancer: a nationwide population-based study. Clin Breast Cancer.

[42] Choi Y.J., Lee D.H., Han K.Do. (2020). Association between high fatty liver index and development of colorectal cancer: a nationwide cohort study with 21,592,374 Korean. Korean J Intern Med.

[43] Lee C.-H., Han K.-D., Kim D.H., Kwak M.-S. (2021). The repeatedly elevated fatty liver index is associated with increased mortality: a population-based cohort study. Front Endocrinol (Lausanne).

