# Supplementary material for: Role of fatty liver index in risk-stratifying comorbid disease outcomes in non-alcoholic fatty liver disease
Source: JHEP Rep. 2023 Aug 24;5(12):100896. doi: 10.1016/j.jhepr.2023.100896 (PMC10624587; doi:10.1016/j.jhepr.2023.100896)
Supplement: Multimedia component 1 [file mmc1.pdf]

Role of fatty liver index in risk-stratifying comorbid disease outcomes in non-alcoholic fatty liver disease

Brian Ho, Andrew Thompson, Andrea L Jorgensen, Munir Pirmohamed

Table of contents

Supplementary materials...2

Supplementary figures..6

Supplementary tables..14

Supplementary methods

Disease Outcomes/Events Definitions

All disease covariates and outcomes were identified using ICD-9/ICD-10, primary care and death registry information. UK cancer registry data was used for identification of additional cases of hepatic and extrahepatic malignancies. Similarly, for diabetes, HBA1c measurements performed during UKB assessment attendances, using the WHO threshold of >48 mmol/mol were used, while GP prescription data of antidiabetic drugs were used to find additional cases of T2DM. Lastly GP prescription of statin or fibrates were also used to identify individuals with dyslipidaemia. Specific metabolic syndrome/metabolism related malignancy outcomes were also extracted using diagnostic codes, which are colon, breast and upper GI (oesophageal and stomach) cancers. Incidence rate of diseases were calculated for each FLI category.

Receiver Operating Characteristic Analysis

Receiver operating characteristics were examined and area under the curve was used to assess FLI, hepatic steotosis index, and lipid accumulation product performance in identifying combined prevalent and incident NAFLD, i.e a cross-sectional analysis. Case definitions were defined by MRI-PDFF value derived by two separate companies for the UK Biobank (Perspectum Diagnostics and AMRA). Additional case finding was performed using ICD9/10 clinical codes and primary care record data, which both shared in their definition. These non-invasive scores was therefore tested against two case control definitions of NAFLD in a small subset of UK Biobank participants which have available MRI and healthcare records data. Optimum cut off for was also derived using greatest distance from line of no-effect, i.e 0.50 AUC line. AUROC was calculated and 95% CIs of ROC sensitivities were obtained through stratified

bootstrapping data 2000 times at intervals of 5% for specificities and the distribution smoothened with the loess method.

Time to Event Analysis

Cox proportional hazards model was used for time to event analyses. Study start date was defined at recruitment to UK Biobank when FLI score was derived. Listwise deletion dataset was used in Cox regression modelling and individuals who have experienced the event of interest prior to study start date were excluded from the respective time to event analysis. Univariate regression was first performed for each time to event outcome for exposures of interest. A p-value of <0.1 was used as threshold for inclusion for downstream multivariate analysis. To assess linearity of Cox regression model, penalised splines were applied to continuous covariates to assess for significant non-linear component in univariate cox regression. Exposure covariates with significant non-linear component ($p < 0.05$) were subsequently regressed by applying a natural cubic spline for better model fitting. T2DM, as an exposure of interest, was fitted as a time dependent covariate for all models, except in the model where T2DM is the event of interest. Subsequently, two models were fitted to assess risk stratification by FLI: the first model is termed the "Basic Model", which included covariates age and sex, and the second model included additional covariates brought forward from univariate regression. Finally, proportional hazards assumption was tested in these models using Schoenfeld residuals. If breaches of assumptions were found, defined again by significance level of $p<0.05$, time dependent coefficients were created using a step function, which divided follow-up from FLI into 0-3, 4-6, 7-9 and >9 years. This division in follow-up period as a step function from UK Biobank recruitment was chosen from the variation in median >10 years observed in our UK Biobank participants in all our time to event analyses. Models whereby FLI was treated

as categorical variable was additionally assessed with likelihood ratio test between models with and without FLI as a covariate. This allowed testing whether FLI provided additional predictive risk stratification for event of interest as a single covariate. Both dummy variable p-values within the model and model comparison tests p-values are reported. P-values <0.05 were considered significant after Bonferonni correction for multiple testing (this is 26 models/tests for FLI, $p < 0.0019$)

Additional and Sensitivity Analyses

An additional analysis was performed to look at incidence rates of misclassified individuals, with high-risk FLI but normal livers on MRI and low-risk FLI with evidence/diagnosis of NAFLD. Two sensitivity analyses were performed. Firstly, examining time to event analysis using components used to calculate FLI (BMI, waist circumference, serum GGT and total triglyceride levels) in multivariate Cox regression. This is to allow estimate of significance and risk estimate direction for each of these components toward the incident disease outcome. Secondly, listwise deletion cohort with/without accounting for missing data from alcohol intake for each time to event analyses was examined. Alcohol has the highest overall proportion of missing data of 31% and allows best chance to see examine whether exclusion of missing broadly altered the model risk estimates.

Incident All-cause Mortality with FLI and Fibrosis Scores

For incident all-cause mortality, a similar approach was adopted for Cox regression analysis investigating risk stratification by FLI and non-invasive fibrosis scores (NAFLD Fibrosis Score and FIB4). Univariate modelling and assumptions testing was performed similarly as detailed above for FLI on incident disease outcomes. Separate multivariate modelling for fibrosis scores was further performed after

stratifying individuals by FLI risk to examine NFS and FIB4's performance in these subgroups. Further multivariate modelling included FLI, and a single fibrosis score (NFS or FIB4) was used or with all exposure covariates brought forward by univariate analysis. This allowed examining differences in risk effect estimates given by fibrosis scores and the effect exposure covariates have. P-values <0.05 were considered significant after Bonferonni correction for multiple testing.

Supplementary figures

Fig. S1 – NAFLD Case Definition Flowchart

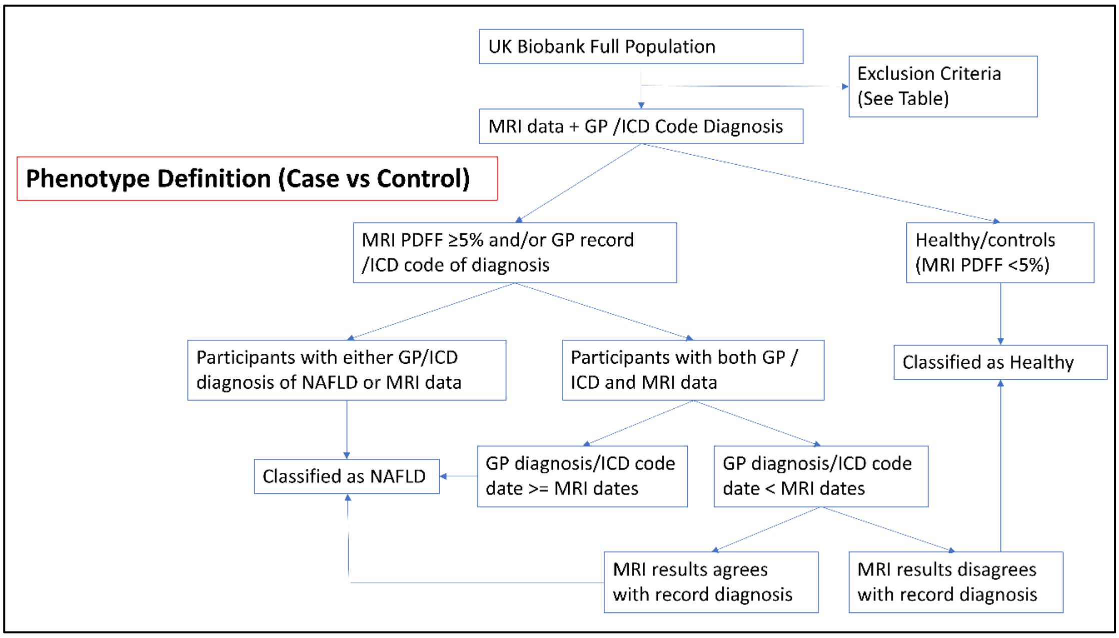

Flowchart shows the derivation of case control definition of NAFLD used based on inpatient hospital ICD diagnostic codes, primary care record data, and available MRI proton density fat fraction generated by companies Perspectum Diagnostic and AMRA. The case-control provides an assessment of ground truth for analysing FLI's capability in cross-sectional disease identification (i.e identifying both incident and prevalent NAFLD).

Fig. S2 - Diagnostic Performance at Low and High Cut-offs of FLI

Diagram here shows the equation used to derive Fatty Liver Index score as published by Bedogni et al, 2016. Cut-offs chosen here are recommended cut-offs for stratifying risk of NAFLD at point of care. Frequency tables and cross-sectional diagnostic performance (sensitivity, specificity, positive predictive value (PPV) and negative predictive value (NPV)) are shown for these cut-offs is being used as a binary classifier for NAFLD. Our NAFLD ground truth is defined by diagnostic codes and MRI measurements (See Figures S1). The 1st definition denotes case control definition derived from MRI proton density fat fraction from Perspectum Diagnostics and 2nd definition originates from AMRA; both cohorts haring clinically/primary care coded cases.

Fatty Liver Index = $e^y / (1 + e^y) \times 100$

y = 0.953 x ln(triglyceride levels (mg/dL) + 0.139 x BMI (kg/m2) + 0.718 x ln(GGT (U/L)) + 0.053 x (waist circumference - 15.745)

1st Case Control Definition

Low cut off (≥30)

Case/Control<br>---------------------------------<br>Index Classification	Healthy	NAFLD
Healthy	1182	379
NAFLD	1029	5066

Sensitivity – 93.04%
Specificity – 53.46%
PPV – 75.03%
NPV – 83.63%

High cut-off (≥60)

Case/Control<br>---------------------------------<br>Index Classification	Healthy	NAFLD
Healthy	1781	1346
NAFLD	430	4099

Sensitivity – 75.28%
Specificity – 80.55%
PPV – 56.04%
NPV – 90.82%

2nd Case Control Definition

Low cut off (≥30)

Case/Control<br>---------------------------------<br>Index Classification	Healthy	NAFLD
Healthy	2489	459
NAFLD	2174	5655

Sensitivity – 92.49%
Specificity – 53.38%
PPV – 84.15%
NPV – 72.65%

High cut-off (≥60)

Case/Control<br>---------------------------------<br>Index Classification	Healthy	NAFLD
Healthy	3758	1627
NAFLD	905	4487

Sensitivity – 73.39%
Specificity – 80.59%
PPV – 69.34%
NPV – 83.51%

Fig. S3 – Listwise Deletion Flow Chart for Outcomes of Interest

Flowchart showing derivation of the listwise deletion dataset from UK Biobank for each incident disease outcome. The final numbers were subsequently used in each individual time to event analysis.

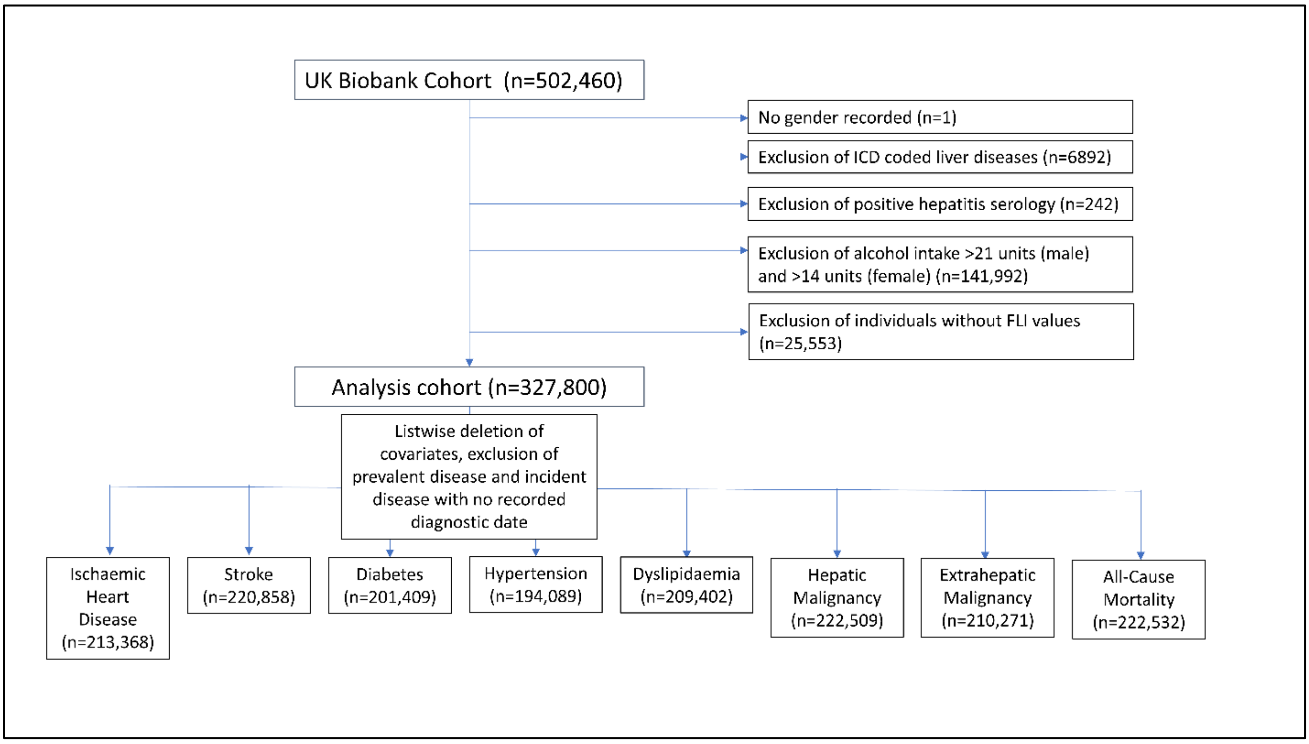

Fig. S4 – Incident All-cause Mortality By Fibrosis Scores

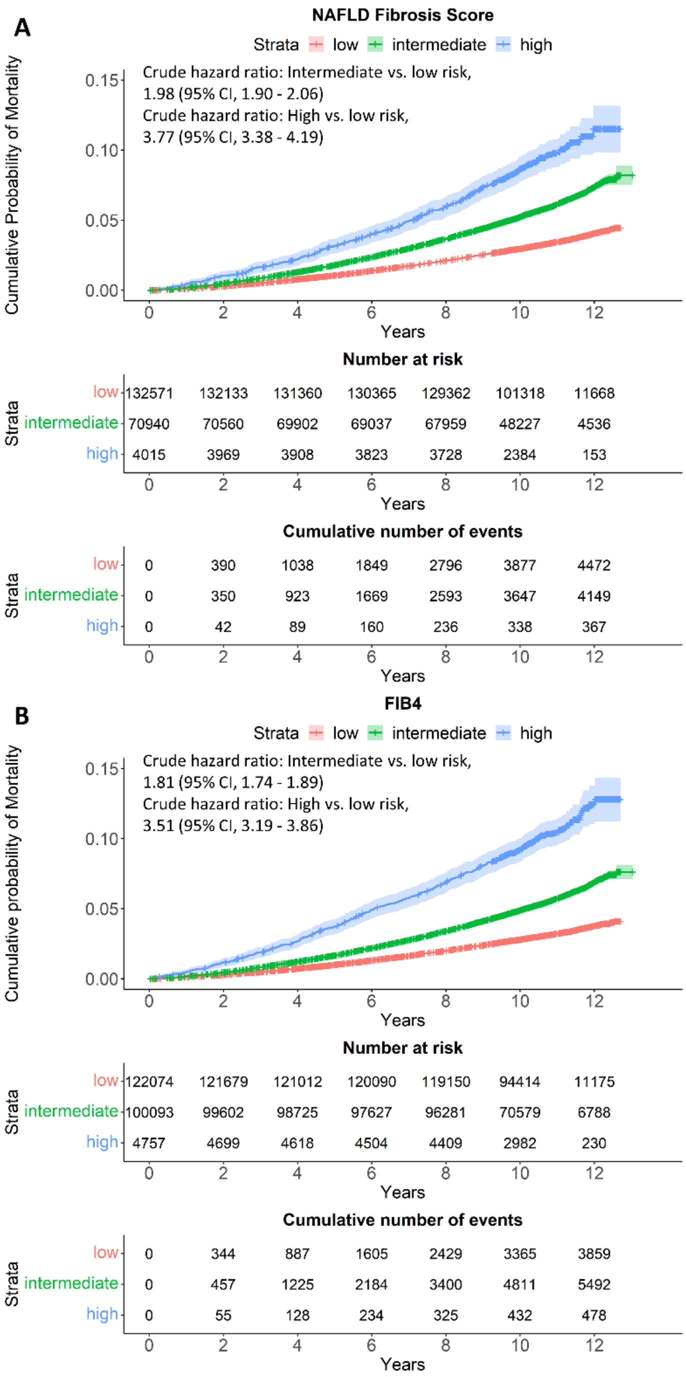

Kaplan-Meier Curves showing incident all-cause mortality comparing individuals with low, intermediate and high-risk (A) NAFLD fibrosis score and (B) FIB4. Crude hazard ratios and 95% CI (shaded area) are derived from univariate Cox regression modelling to give broad interpretation of risk. + is used to mark individuals who are censored.

Fig. S5 – Incident All-cause Mortality By Fibrosis Scores Stratified By FLI Risk

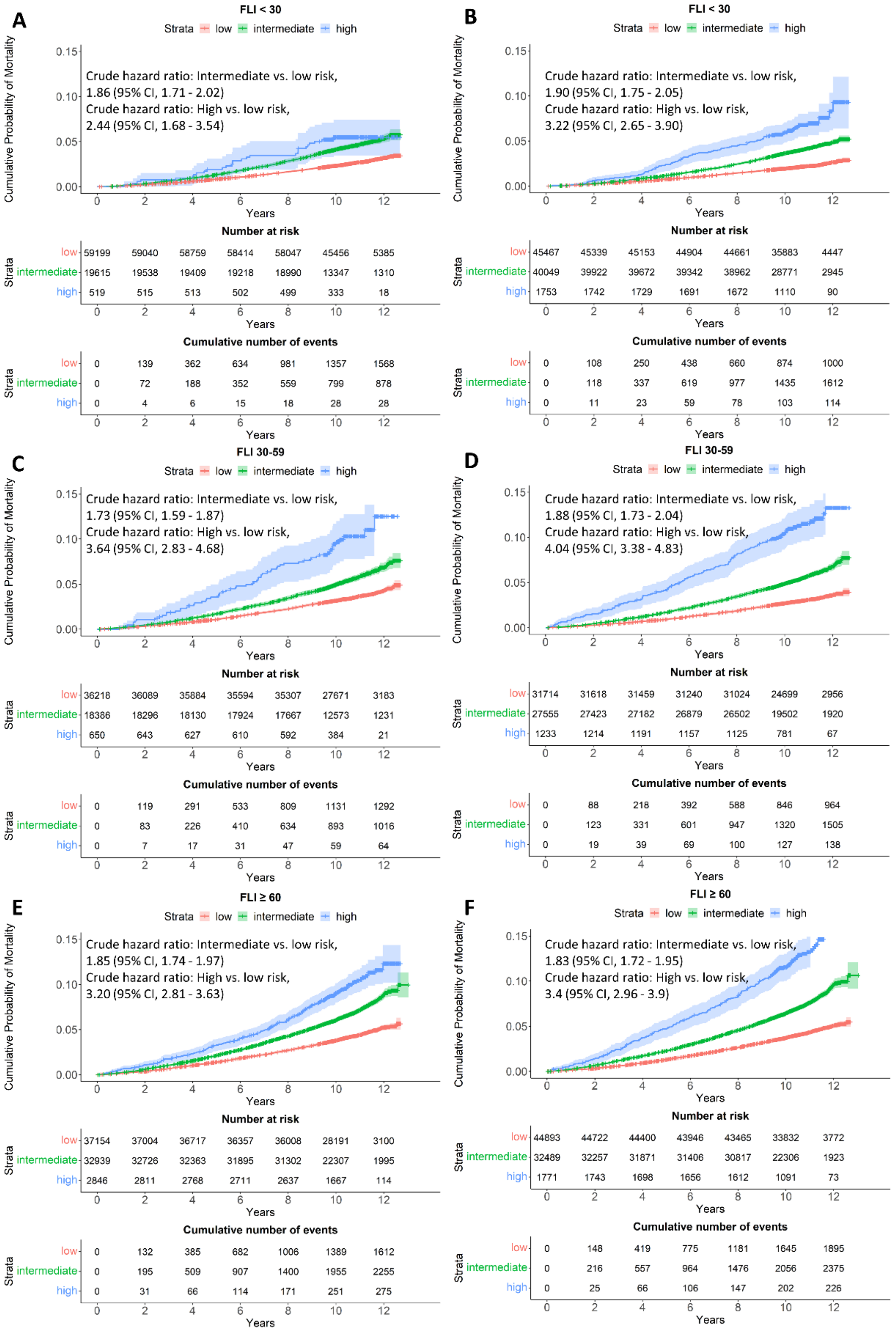

The Kaplan-Meier Curves are stratified by FLI risk categories: low – first row, intermediate – second row, high – third row. Cumulative incidence by risk categories of NAFLD fibrosis score (right column) and FIB4 (left column) are shown. Crude hazard ratios and 95% Cis (shaded area) are obtained from univariate Cox regression analysis. + signs mark censored individuals.

Fig. S6 - Unified Model FIB4 or NAFLD Fibrosis Score with FLI For All-Cause Mortality

Model	Term	Follow-up Period	HR (95% CI)	p-value
FLI + FIB4	Fibrosis score intermediate vs. low risk	0-3 years	1.8 (1.61-2)	2.28e-258
		4-6 years	1.68 (1.55-1.83)	
		7-9 years	1.89 (1.76-2.03)	
		>9 years	2.06 (1.9-2.23)	
	Fibrosis score high vs. low risk	0-3 years	4.03 (3.23-5.04)	
		4-6 years	3.78 (3.18-4.5)	
		7-9 years	3.13 (2.64-3.71)	
		>9 years	3.33 (2.7-4.11)	
FLI + FIB4 + Covariates	Fibrosis score intermediate vs. low risk	0-3 years	1.06 (0.94-1.18)	1.23e-11
		4-6 years	0.94 (0.86-1.03)	
		7-9 years	1.01 (0.94-1.09)	
		>9 years	1.05 (0.96-1.15)	
	Fibrosis score high vs. low risk	0-3 years	1.85 (1.46-2.33)	
		4-6 years	1.61 (1.34-1.93)	
		7-9 years	1.26 (1.06-1.5)	
		>9 years	1.27 (1.03-1.58)	
FLI + NFS	Fibrosis score intermediate vs. low risk	0-3 years	1.68 (1.51-1.88)	1.93e-198
		4-6 years	1.71 (1.57-1.86)	
		7-9 years	1.83 (1.7-1.97)	
		>9 years	2 (1.84-2.18)	
	Fibrosis score high vs. low risk	0-3 years	3.39 (2.63-4.37)	
		4-6 years	2.89 (2.34-3.57)	
		7-9 years	3.21 (2.69-3.84)	
		>9 years	3.23 (2.55-4.08)	
FLI + NFS + Covariates	Fibrosis score intermediate vs. low risk	0-3 years	1.04 (0.93-1.16)	0.00487
		4-6 years	1.01 (0.92-1.1)	
		7-9 years	1.03 (0.95-1.11)	
		>9 years	1.05 (0.96-1.15)	
	Fibrosis score high vs. low risk	0-3 years	1.6 (1.23-2.07)	
		4-6 years	1.24 (1-1.53)	
		7-9 years	1.27 (1.05-1.52)	
		>9 years	1.15 (0.9-1.46)	

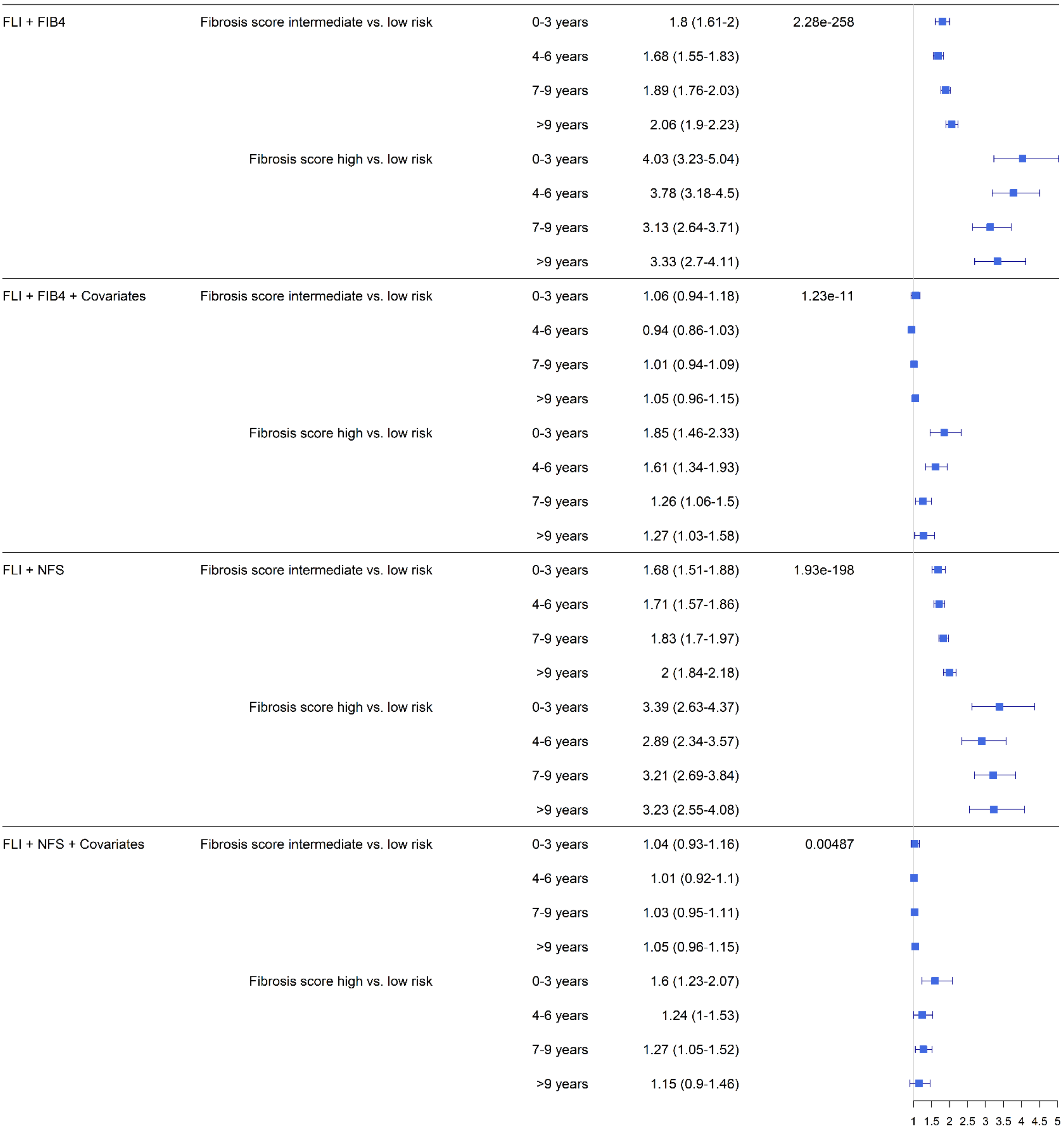

Forest plot showing hazard ratios of intermediate and high-risk non-invasive fibrosis score from Cox regression models. Regressors include FLI alone and FLI with other covariate exposures (age, sex, Townsend Index, smoking status, alcohol intake and T2DM diagnosis). Both FLI and fibrosis scores were treated as dummy variables here. Proportional hazard assumption was violated in all models and thus time dependent coefficients were utilised, dividing follow-up period into intervals of 0-3, 4-6, 7-9 and >9 years from time of risk score calculation. P-values are derived from likelihood ratio test between models with and without (null model) fibrosis score as a regressor.

Supplementary tables

Table S1 – ICD 9 Diagnostic Codes and Definitions For Exclusion

**VIRAL HEPATITIS**	
0700	Viral hepatitis a with hepatic coma
0701	Viral hepatitis a without mention of hepatic coma
0702	Viral hepatitis b with hepatic coma
0703	Viral hepatitis b without mention of hepatic coma
0704	Other specified viral hepatitis with hepatic coma
0705	Other specified viral hepatitis without mention of hepatic coma
0706	Unspecified viral hepatitis with hepatic coma
0709	Unspecified viral hepatitis without mention of hepatic coma
5732	Hepatitis in other infectious diseases classified elsewhere
5731	Hepatitis in viral diseases classified elsewhere
V026	Carrier or suspected carrier of viral hepatitis
77440	Perinatal jaundice due to hepatocellular damage (from hepatitis a)
77441	Perinatal jaundice due to hepatocellular damage (from hepatitis b)
77448	Perinatal jaundice due to other specified hepatitis
**ALCOHOL LIVER DISEASE**	
5710	Alcoholic fatty liver
5711	Acute alcoholic hepatitis
5712	Alcoholic cirrhosis of liver
5713	Alcoholic liver damage, unspecified
**AUTOIMMUNE LIVER DISEASE**	
5714	Chronic hepatitis
**WILSON’s DISEASE**	
2751	Disorders of copper metabolism
**BILIARY CIRRHOSIS**	
5716	Biliary Cirrhosis
**IRON METABOLISM RELATED LIVER DISEASE**	
27509	Disorders of iron metabolism (other and unspecified)
27501	Haemosiderosis
27502	Haemochromatosis

**ALPHA-1 ANTITRYPSIN DEFICIENCY**	
27761	Other deficiencies of circulating enzymes (alpha-1-antitrypsin hepatitis)
27762	Other alpha-1-antitrypsin deficiency
**HEPATOVENOUS DISEASE**	
4530	Budd-chiari syndrome
**PRIMARY SCLEROSING CHOLANGITIS**	
5761	Cholangitis
**OTHER**	
27103	Glycogenosis (associated with hepatic cirrhosis)
57152	Cirrhosis of liver without mention of alcohol (childhood function) - Indian childhood

Table S2 – ICD 10 Diagnostic Codes and Definitions For Exclusion

**VIRAL HEPATITIS**	
B160	Acute hepatitis B with delta-agent (coinfection) with hepatic coma
B161	Acute hepatitis B with delta-agent (coinfection) without hepatic coma
B162	Acute hepatitis B without delta-agent with hepatic coma
B169	Acute hepatitis B without delta-agent and without hepatic coma
B171	Acute hepatitis C
B172	Acute hepatitis E
B178	Other specified acute viral hepatitis
B179	Acute viral hepatitis, unspecified
B180	Chronic viral hepatitis B with delta-agent
B181	Chronic viral hepatitis B without delta-agent
B182	Chronic viral hepatitis C
B189	Chronic viral hepatitis, unspecified
B190	Unspecified viral hepatitis with coma
B199	Unspecified viral hepatitis without coma
B251	Cytomegaloviral hepatitis
B942	Sequelae of viral hepatitis
O984	Viral hepatitis complicating pregnancy, childbirth and the puerperium
P353	Congenital viral hepatitis
Z225	Carrier of viral hepatitis
**ALCOHOL LIVER DISEASE**	
K700	Alcoholic fatty liver
K701	Alcoholic hepatitis
K702	Alcoholic fibrosis and sclerosis of liver
K703	Alcoholic cirrhosis of liver
K704	Alcoholic hepatic failure
K709	Alcoholic liver disease, unspecified
**TOXIC LIVER DISEASE**	
K710	Toxic liver disease with cholestasis
K711	Toxic liver disease with hepatic necrosis
K712	Toxic liver disease with acute hepatitis

K713	Toxic liver disease with chronic persistent hepatitis
K715	Toxic liver disease with chronic active hepatitis
K716	Toxic liver disease with hepatitis, not elsewhere classified
K717	Toxic liver disease with fibrosis and cirrhosis of liver
K718	Toxic liver disease with other disorders of liver
K719	Toxic liver disease, unspecified
K753	Granulomatous hepatitis, not elsewhere classified
**AUTOIMMUNE DISEASE**	
K754	Autoimmune hepatitis
**WILSON’S DISEASE**	
E830	Disorders of copper metabolism
**PRIMARY BILIARY CIRRHOSIS**	
K743	Primary biliary cirrhosis
K744	Secondary biliary cirrhosis
K745	Biliary cirrhosis, unspecified
**IRON METABOLISM RELATED LIVER DISEASE**	
E831	Disorders of iron metabolism

S3 – ICD and Primary Care (Read2 & Read3) Codes for NAFLD Classification

The table denotes diagnostic codes and respective clinical definitions used for case finding of NAFLD individuals in the UK Biobank (ICD HESIN and GP primary care dataset). These are used in conjunction with MRI data.

ICD Code	Definition
K760 (ICD10)	Fatty (change of) liver, not elsewhere classified
5718 (ICD9)	Other chronic non-alcoholic liver disease
**Read2**	**Definition**
J61y	Other non-alcoholic chronic liver disease
J61y1	Non-alcoholic fatty liver
J61y7	Steatosis of liver
J61y9	Fatty Change of Liver
**Read3**	**Definition**
J61y.	Other non-alcoholic chronic liver disease
J61y1	Non-alcoholic fatty liver
J61y7	Steatosis of liver
X307v	Fatty change of liver

S4 - Incidence Rates of Disease Outcomes by FLI Category

Table shown looks at number of individuals at risk, person-years of and event frequency for each FLI risk groups over the follow-up period: <30 (low risk), 30-59 (intermediate risk) and ≥60 (high risk). Incident disease per 100-person years subsequently calculated and shown here.

Disease Outcome	FLI Risk group	person-years	n	Event Frequency	Event per 100-person years
Ischaemic Heart Disease	Low	903312	86112	2827	0.31
	Intermediate	590376	57349	3645	0.62
	High	703059	69907	6513	0.93
Stroke	Low	923394	87310	971	0.11
	Intermediate	621927	59311	1060	0.17
	High	770244	74237	1527	0.20
Hypertension	Low	829440	82193	8844	1.07
	Intermediate	501032	52403	10153	2.03
	High	530773	59493	17263	3.25
Dyslipidaemia	Low	881732	85227	4905	0.56
	Intermediate	563581	56262	6061	1.08
	High	655883	67913	10817	1.65
Type 2 Diabetes Mellitus	Low	860877	82016	2124	0.25
	Intermediate	568649	54924	2487	0.44
	High	644163	64469	6660	1.03
Hepatic Malignancy	Low	932102	87716	67	0.01
	Intermediate	631477	59762	57	0.01
	High	785207	75031	133	0.02
Extrahepatic Malignancy	Low	769905	79256	9740	1.22
	Intermediate	534473	53902	7875	1.47
	High	670190	68153	10423	1.56

S5- Unadjusted Incidence Rates of Comorbidites in Individuals Misclassified by FLI with and without NAFLD.

	Incidence (per 100-person years)			
	Perspectum Algorithm Definition		AMRA Definition	
Outcome	Low-Risk FLI with NAFLD	High Risk FLI without NAFLD	Low-Risk FLI with NAFLD	High Risk FLI without NAFLD
Ischaemic Heart Disease	0.27	0.26	0.27	0.3
Ischaemic Stroke	0.025	0.087	0.021	0.052
Type 2 Diabetes Mellitus	0.27	0.27	0.27	0.18
Hypertension	0.92	1.28	0.88	1.08
Dyslipidaemia	0.58	0.88	0.51	0.73
Hepatic Malignancy	0	0.021	0	0.01
Extrahepatic Malignancy	0.94	0.69	0.77	0.61
All-cause Mortality	0.51	0.085	0.44	0.11

Table showing incidence rate of comorbid outcomes in misclassified individuals whereby they have low-risk FLI but found to have hepatic steatosis and high-risk FLI, but normal liver based on MRI-PDFF and ICD9/10 classification. The definition of NAFLD is based on MRI-PDFF values derived by Perspectum Diagnostics (our first definition) and AMRA (our second definition), whereas the diagnostic code is obtained from a common source: diagnostic codes from hospital inpatient records.

S6 - Univariate Cox Regression: FLI and Covariates for Disease Outcomes

Table showing univariate cox regression modelling outputs (hazards ratios and 95% CI) with associated with p-values disease outcomes with all exposures of interest. These are age, alcohol intake and Townsend index were treated as continuous variables, whereas sex, diabetes smoking status, FLI were treated as categorical variables using dummy variables. P <0.1 was used as the threshold for variable selection for downstream multivariate regression modelling.

Outcome	Covariate	HR (95% CI)	p-value
Ischaemic Heart Disease	Age (years)	1.08 (1.08 - 1.09)	<1 E-100
	Male (reference Female)	1.79 (1.74 - 1.83)	<1 E-100
	Alcohol Intake (units/week)	1.02 (1.01- 1.02)	3.26E-23
	Previous smoker (reference non-smoker)	1.48 (1.42 - 1.54)	3.68E-77
	Current smoker (reference non-smoker)	0.88 (0.85 - 0.91)	1.45E-14
	Diabetes (reference healthy)	1.62 (1.55 - 1.69)	1.86E-101
	Townsend Index	1.03 (1.02 - 1.03)	6.60E-17
	FLI intermediate vs. low risk	1.98 (1.88 - 2.08)	1.34E-162
	FLI high vs. low risk	2.97 (2.84 - 3.1)	<1 E-100
Stroke	Age (years)	1.10 (1.10 - 1.10)	7.75E-288
	Male (reference Female)	1.37 (1.31 - 1.44)	1.08E-40
	Alcohol Intake (units/week)	1.01 (1.01 - 1.02)	2.55E-04
	Previous smoker (reference non-smoker)	1.32 (1.22 - 1.43)	2.34E-11
	Current smoker (reference non-smoker)	0.91 (0.86 - 0.97)	5.83E-03
	Diabetes (reference healthy)	1.54 (1.42 - 1.68)	2.06E-24
	Townsend Index	1.02 (1.01 - 1.03)	5.90E-05
	FLI intermediate vs. low risk	1.62 (1.49 - 1.77)	1.29E-27
	FLI high vs. low risk	1.89 (1.74 - 2.05)	3.56E-54
Hypertension	Age (years)	1.08 (1.08 - 1.08)	<1 E-100
	Male (reference Female)	1.26 (1.24 - 1.27)	1.35E-206

	Alcohol Intake (units/week)	1.00 (1.00 - 1.01)	4.25E-03
	Previous smoker (reference non-smoker)	1.19 (1.16 - 1.23)	9.69E-38
	Current smoker (reference non-smoker)	0.85 (0.83 - 0.87)	7.75E-53
	Diabetes (reference healthy)	1.54 (1.50 - 1.59)	2.30E-188
	Townsend Index	1.03 (1.02 - 1.03)	2.11E-44
	FLI intermediate vs. low risk	1.9 (1.85 - 1.96)	<1 E-100
	FLI high vs. low risk	3.05 (2.98 - 3.13)	<1 E-100
Dyslipidaemia	Age (years)	1.08 (1.08 - 1.08)	<1 E-100
	Male (reference Female)	1.34 (1.32 - 1.37)	2.10E-208
	Alcohol Intake (units/week)	1.00 (1.00 - 1.01)	4.17E-02
	Previous smoker (reference non-smoker)	1.41 (1.37 - 1.46)	1.57E-95
	Current smoker (reference non-smoker)	0.88 (0.86 - 0.91)	2.04E-21
	Diabetes (reference healthy)	1.76 (1.70 - 1.82)	8.89E-238
	Townsend Index	1.05 (1.04 - 1.05)	3.66E-86
	FLI intermediate vs. low risk	1.93 (1.86 - 2.01)	3.36E-258
	FLI high vs. low risk	2.96 (2.87 - 3.07)	0.00E+00
Diabetes	Age (years)	1.05 (1.04 - 1.05)	3.00E-267
	Male (reference Female)	1.36 (1.32 - 1.40)	6.55E-117
	Alcohol Intake (units/week)	1.00 (0.99 - 1.00)	5.04E-03
	Previous smoker (reference non-smoker)	1.33 (1.27 - 1.39)	2.79E-34
	Current smoker (reference non-smoker)	0.92 (0.88 - 0.95)	1.39E-06
	Townsend Index	1.04 (1.04 - 1.05)	4.83E-41
	FLI intermediate vs. low risk	1.77 (1.67 - 1.88)	1.33E-83
	FLI high vs. low risk	4.19 (3.99 - 4.4)	<1 E-100
Hepatic Malignancy	Age (years)	1.09 (1.07 - 1.11)	1.43E-18
	Male (reference Female)	1.22 (1.03 - 1.45)	2.42E-02
	Alcohol Intake (units/week)	0.99 (0.97 - 1.02)	5.53E-01
	Previous smoker (reference non-smoker)	1.07 (0.75 - 1.51)	7.10E-01
	Current smoker (reference non-smoker)	0.73 (0.57 - 0.95)	1.67E-02
	Diabetes (reference healthy)	2.62 (2.01 - 3.43)	2.10E-12
	Townsend Index	0.99 (0.95 - 1.04)	6.91E-01

	FLI intermediate vs. low risk	1.26 (0.88 - 1.79)	2.06E-01
	FLI high vs. low risk	2.36 (1.76 - 3.17)	1.00E-08
Extrahepatic Malignancy	Age (years)	1.066(1.065 -1.068)	<1 E-100
	Male (reference Female)	1.17 (1.151 -1.19)	1.69E-77
	Alcohol Intake (units/week)	1.012 (1.009 -1.014)	3.37E-25
	Previous smoker (reference non-smoker)	1.116 (1.082 -1.151)	5.46E-12
	Current smoker (reference non-smoker)	0.874 (0.853 -0.895)	3.58E-28
	Diabetes (reference healthy)	1.025 (0.99 -1.061)	0.16
	Townsend Index	0.978 (0.974 -0.982)	8.95E-26
	FLI intermediate vs. low risk	1.084(1.057-1.113)	1.07E-09
	FLI high vs. low risk	1.119(1.092-1.147)	7.79E-20

S7 – Univariate Cox Regression Analysis for Incident All-cause Mortality

Outcome	Covariate	HR (95% CI)	p-value
All-Cause Mortality	Age (years)	1.107 (1.104 - 1.111)	<1E-100
	Male (reference Female)	1.478 (1.437 - 1.519)	3.13E-167
	Alcohol Intake (units/week)	1.01 (1.006 - 1.013)	2.39E-07
	Previous smoker (reference non-smoker)	1.936 (1.856 - 2.019)	2.45E-206
	Current smoker (reference non-smoker)	0.914 (0.883 - 0.946)	3.35E-07
	Diabetes (reference healthy)	1.997 (1.908 - 2.09)	9.06E-194
	Townsend Index	1.044 (1.037 - 1.05)	8.68E-40
	FLI intermediate vs. low risk	1.42 (1.35-1.50)	1.13E-38
	FLI high vs. low risk	1.96 (1.87-2.06	5.3E-176
	FIB4 intermediate vs. low risk	1.81 (1.74-1.89)	2.5E-176
	FIB4 high risk vs. low risk	3.51 (3.19-3.86)	7.1E-148
	NFS intermediate risk vs. low risk	1.98 (1.90-2.06)	2E-220
	NFS high risk vs. low risk	3.77 (3.38-4.19)	1.4E-131

Table showing hazards ratios (95% CI) for univariate Cox regression outputs in time to event analysis for incident all-cause mortality with all exposure covariates of interest. Categorical variables are analysed as dummy variables.

S8 – Adjusted Model Outputs of Cox Regression for Incident Diseases of Interest

Table output of multivariate cox regression analysis for incident ischaemic heart disease, stroke, hypertension, dyslipidaemia, hepatic malignancy, and extrahepatic malignancy. This is replicate data from the main article's forest plot, but p-values for the regression dummy variable created for FLI risk categories is shown here, with the significance column showing whether level reaches Bonferonni-correction threshold.

	**FLI (Intermediate vs. Low Risk)**			**FLI (High vs. Low Risk)**		
Follow Up Period	HR (95% CI)	p-value	Significance	HR (95% CI)	p-value	Significance
**Ischaemic Heart Disease**						
0-3 years	1.47 (1.32 - 1.63)	2.13E-12	*	2.14 (1.94 - 2.36)	4.78E-52	*
4-6 years	1.38 (1.25 - 1.52)	6.10E-11	*	1.97 (1.80 - 2.15)	1.71E-50	*
7-9 years	1.45 (1.33 - 1.59)	3.40E-16	*	1.80 (1.65 - 1.96)	3.08E-42	*
>9 years	1.31 (1.17 - 1.47)	2.61E-06	*	1.66 (1.49 - 1.85)	1.11E-20	*
**Stroke**						
Entire	1.21 (1.11 - 1.33)	2.55E-05	*	1.31 (1.20 - 1.42)	1.26E-09	*
**Diabetes**						
0-3 years	1.83 (1.59 - 2.10)	1.58E-17	*	4.55 (4.04 - 5.12)	2.81E-139	*
4-6 years	1.36 (1.25 - 1.48)	2.56E-13	*	2.69 (2.50 - 2.88)	6.14E-162	*
7-9 years	2.23 (1.97 - 2.52)	3.19E-36	*	5.69 (5.11 - 6.34)	4.48E-217	*
>9 years	2.76 (2.28 - 3.34)	2.20E-25	*	7.05 (5.95 - 8.34)	3.77E-113	*
**Hypertension**						
0-3 years	1.74 (1.64 - 1.83)	5.54E-87	*	2.84 (2.70 - 2.98)	<1.00E-100	*
4-6 years	1.63 (1.55 - 1.72)	2.59E-71	*	2.61 (2.48 - 2.74)	<1.00E-100	*
7-9 years	1.59 (1.51 - 1.68)	4.98E-64	*	2.42 (2.30 - 2.55)	1.66E-267	*
>9 years	1.48 (1.38 - 1.59)	4.39E-26	*	2.36 (2.21 - 2.53)	1.30E-142	*
**Dyslipidaemia**						
0-3 years	1.70 (1.58 - 1.83)	2.33E-47	*	2.48 (2.32 - 2.64)	2.31E-163	*
4-6 years	1.52 (1.41 - 1.63)	9.41E-31	*	2.09 (1.96 - 2.23)	1.87E-111	*
7-9 years	1.55 (1.44 - 1.67)	2.50E-32	*	2.15 (2.01 - 2.30)	3.26E-114	*
>9 years	1.40 (1.28 - 1.54)	1.34E-12	*	2.05 (1.89 - 2.23)	7.61E-64	*
**Hepatic Malignancy**						
Entire	1.01 (0.70 - 1.45)	0.965429969		1.69 (1.23 - 2.32)	0.001	*
**Extrahepatic Malignancy**						
0-3 years	0.91 (0.86 - 0.97)	0.002	*	0.93 (0.88 - 0.99)	0.014	
4-6 years	1.04 (0.99 - 1.10)	0.146		1.04 (0.98 - 1.10)	0.172	
7-9 years	1.04 (0.98 - 1.10)	0.198		1.10 (1.03 - 1.16)	0.001	*
>9 years	0.94 (0.88 - 1.02)	0.169		1.02 (0.96 - 1.11)	0.4553	

S9 – Cox Regression Analysis of Non-Invasive Fibrosis Score in High Risk FLI Individuals

Non-invasive fibrosis scores		intermediate risk		high risk	
	Follow-up Period	HR (95% CI)	p-value	HR (95% CI)	p-value
FIB4	0-3 Years	1.16 (0.98 - 1.38)	0.08	1.91 (1.37 - 2.66)	0.0002
	3-6 Years	0.94 (0.82 - 1.06)	0.31	1.53 (1.16 - 2.00)	0.0022
	6-9 Years	1.03 (0.92 - 1.16)	0.56	1.42 (1.11 - 1.83)	0.0061
	>9 Years	1.07 (0.94 - 1.22)	0.29	1.40 (1.03 - 1.89)	0.0308
NAFLD Fibrosis Scores	Entire	1.05 (0.98 - 1.13)	0.13	1.28 (1.12 - 1.46)	0.0004

The table shows hazards ratios for multivariate Cox regression models of intermediate and high-risk FIB4 and NAFLD Fibrosis score (reference: low risk) in UKB participants with high-risk FLI. All exposure covariates of interest are included. FIB4 did not meet proportional hazards assumption on regression, thus the risk estimates were modelled at three yearly intervals to allow estimation of time dependent coefficients.

S10- Multivariable Covariate Adjusted Regression Outputs with Components of FLI

Outcome	Ischaemic Heart Disease		Ischaemic Stroke		Type 2 Diabetes Mellitus		Hypertension		Dyslipidaemia		Hepatic Malignancy		Extrahepatic Malignancy		All-cause Mortality	
FLI Component	HR (95%CI)	p	HR (95%CI)	p	HR (95%CI)	p	HR (95%CI)	p	HR (95%CI)	p	HR (95%CI)	p	HR (95%CI)	p	HR (95%CI)	p
BMI	***1.03 (1.01-1.05)***	***0.000146***	0.99 (0.98-1.00)	0.164	***1.06 (1.05-1.07)***	***1.5E-15***	***1.06 (1.05-1.06)***	***3E-56***	1.02 (1.01-1.02)	7.96E-11	1.03 (0.98-1.08)	0.293	0.99 (0.98-1.00)	0.0002	***0.25 (0.19-0.32)***	***6.54E-27***
Waist Circumference (cm)	***1.01 (1.00-1.01)***	***0.005151***	1.01 (1.01-1.02)	2.67E-06	***1.03 (1.02-1.03)***	***4.3E-18***	***1.01 (1.01-1.02)***	***5E-23***	1.01 (1.01-1.01)	9.16E-18	1.00 (0.98-1.03)	0.731	1.01 (1.00-1.01)	4E-08	1.02 (1.02-1.02)	2.075E-29
GGT (U/L)	1.00 (1.00-1.00)	7.44E-13	1.00 (1.00-1.00)	0.009	***1.00 (1.00-1.00)***	***1.9E-12***	***1.00 (1.00-1.00)***	***5E-19***	1.00 (1.00-1.00)	1.1E-177	1.11 (0.99-1.24)	0.066	1.00 (1.00-1.00)	0.0027	***6.41 (4.80-8.56)***	***3.08E-36***
Total Triglyceride (mmol/L)	1.11 (1.09-1.13)	2.25E-37	1.01 (0.98-1.05)	0.576	1.18 (1.16-1.20)	4.09E-87	1.05 (1.04-1.07)	2E-23	***1.19 (1.17-1.20)***	***9.93E-18***	1.01 (1.00-1.01)	5E-38	***0.95 (0.92-0.97)***	***4E-05***	1.00 (0.95-1.05)	0.89

Table showing multivariate Cox regression model outputs for components of FLI (BMI, waist circumference, GGT and total triglyceride levels) and their associated p-value. Bolded and italicised values indicate that a time-dependent coefficient was fitted for the variable and the values indicate the first three years of follow-up from time of FLI calculation. Splines were not fitted in this instance to allow for ease of interpretation of the significance of the FLI component in predicting the comorbid outcome and its general direction of effect. Caution needs to be had with interpreting this due to likely correlation between these components and the magnitude of its risk effect, which is likely to be imprecise.

S11 – Sensitivity Analysis of Listwise Deletion Estimates Accounting for Missing Data in Alcohol Intake

Table below shows hazard ratios for intermediate and high-risk FLI with reference to low-risk FLI derived from multivariable of Cox proportional hazards models fitted without alcohol intake as covariate, for all comorbid outcomes of interest. Alcohol intake has the highest rate of missing and therefore allow assessment of risk estimates effect towards missing data. Models for each time to event outcome used a listwise deletion dataset accounting (not shaded) and not accounting (shaded) for missing data in alcohol intake to examine differences in risk effect estimation.

		intermediate vs. low risk		High vs. low risk		
Time to Event of Interest	Follow up Period	HR (95% CI)	p-value	HR (95% CI)	p-value	n
CVA	**Entire**	1.21 (1.12 - 1.30)	3.863E-07	1.33 (1.25 - 1.43)	1.34E-16	322837
	Entire	1.21 (1.11 - 1.33)	2.105E-05	1.31 (1.20 - 1.43)	6.05E-10	220858
IHD	**0-3 years**	1.44 (1.38 - 1.50)	5.364E-68	2.02 (1.95 - 2.10)	7.67E-307	310714
	**4-6 years**	1.40 (1.34 - 1.47)	1.013E-47	1.94 (1.86 - 2.02)	3.28E-214	
	**7-9 years**	1.40 (1.32 - 1.48)	2.137E-31	1.87 (1.77 - 1.96)	4.45E-123	
	**>9 years**	1.41 (1.29 - 1.54)	1.112E-13	1.79 (1.65 - 1.94)	3.16E-43	
	0-3 years	1.47 (1.32 - 1.64)	1.292E-12	2.16 (1.96 - 2.38)	2.78E-53	213368
	4-6 years	1.39 (1.26 - 1.53)	3.563E-11	1.99 (1.82 - 2.18)	7.70E-52	
	7-9 years	1.46 (1.33 - 1.60)	1.59E-16	1.82 (1.67 - 1.98)	1.41E-43	
	>9 years	1.32 (1.18 - 1.48)	2.163E-06	1.67 (1.50 - 1.86)	5.02E-21	
Hypertension	**0-3 years**	1.75 (1.67 - 1.83)	1.46E-130	2.89 (2.78 - 3.01)	<1.00 E-100	280617
	**4-6 years**	1.70 (1.63 - 1.78)	1.14E-121	2.72 (2.62 - 2.84)	<1.00 E-100	
	**7-9 years**	1.58 (1.52 - 1.66)	2.74E-92	2.46 (2.37 - 2.56)	<1.00 E-100	
	**>9 years**	1.52 (1.43 - 1.61)	4.29E-45	2.41 (2.28 - 2.53)	5.52E-236	
	0-3 years	1.74 (1.65 - 1.84)	4.78E-88	2.86 (2.72 - 3.00)	<1.00 E-100	194089
	4-6 years	1.64 (1.55 - 1.73)	3.02E-72	2.62 (2.50 - 2.76)	<1.00 E-100	
	7-9 years	1.60 (1.51 - 1.68)	6.96E-65	2.44 (2.32 - 2.56)	5.97E-272	
	>9 years	1.49 (1.38 - 1.60)	1.55E-26	2.38 (2.23 - 2.54)	5.28E-145	
Dyslipidaemia	**0-3 years**	1.72 (1.62 - 1.83)	1.26E-72	2.52 (2.39 - 2.65)	4.07E-257	305075
	**4-6 years**	1.58 (1.49 - 1.67)	1.41E-53	2.20 (2.09 - 2.32)	2.29E-192	
	**7-9 years**	1.55 (1.46 - 1.64)	3.54E-46	2.14 (2.03 - 2.26)	2.30E-169	
	**>9 years**	1.47 (1.37 - 1.59)	3.43E-24	2.09 (1.95 - 2.23)	5.23E-105	

	0-3 years	1.71 (1.59 - 1.84)	3.01E-48	2.51 (2.35 - 2.67)	1.36E-167	209402
	4-6 years	1.53 (1.42 - 1.64)	1.77E-31	2.11 (1.98 - 2.25)	7.93E-115	
	7-9 years	1.56 (1.45 - 1.68)	4.44E-33	2.17 (2.03 - 2.32)	1.71E-117	
	>9 years	1.41 (1.28 - 1.55)	5.63E-13	2.07 (1.91 - 2.26)	1.40E-65	
Diabetes	**0-3 years**	1.92 (1.70 - 2.16)	5.11E-27	5.28 (4.77 - 5.83)	5.20E-232	291437
	**4-6 years**	1.31 (1.22 - 1.40)	4.66E-15	2.68 (2.53 - 2.83)	4.92E-256	
	**7-9 years**	2.20 (2.00 - 2.42)	1.46E-59	5.57 (5.13 - 6.05)	<1.00 E-100	
	**>9 years**	2.57 (2.23 - 2.95)	4.33E-40	6.35 (5.62 - 7.18)	1.52E-191	
	0-3 years	1.69 (1.47 - 1.94)	1.18E-13	1.69 (1.47 - 1.94)	2.61E-130	201409
	4-6 years	1.26 (1.16 - 1.37)	5.31E-08	1.26 (1.16 - 1.37)	2.79E-143	
	7-9 years	2.04 (1.80 - 2.31)	5.46E-29	2.04 (1.80 - 2.31)	3.74E-200	
	>9 years	2.52 (2.08 - 3.05)	2.63E-21	2.52 (2.08 - 3.05)	2.76E-105	
Hepatic Malignancy	**Entire**	0.90 (0.67 - 1.21)	0.4829121	1.66 (1.29 - 2.12)	6.30E-05	325483
	Entire	1.01 (0.70 - 1.45)	0.956358	1.70 (1.24 - 2.33)	1.03E-03	222509
Extrahepatic Malignancy	**0-3 years**	0.99 (0.96 - 1.01)	0.2967836	1.01 (0.99 - 1.04)	0.36550	294213
	**4-6 years**	1.01 (0.98 - 1.04)	0.7210924	1.04 (1.02 - 1.07)	0.00236	
	**7-9 years**	1.00 (0.97 - 1.04)	0.8972509	1.07 (1.04 - 1.11)	0.00005	
	**>9 years**	0.97 (0.92 - 1.04)	0.4213545	1.07 (1.00 - 1.13)	0.03258	
	0-3 years	0.91 (0.85 - 0.97)	0.002405	0.93 (0.87 - 0.98)	0.01289	202879
	4-6 years	1.04 (0.99 - 1.10)	0.1477786	1.04 (0.98 - 1.10)	0.18354	
	7-9 years	1.04 (0.98 - 1.10)	0.201177	1.09 (1.04 - 1.15)	0.00106	
	>9 years	0.95 (0.88 - 1.02)	0.1692405	1.03 (0.96 - 1.11)	0.46389	
All-Cause Mortality	**Entire**	0.99 (0.95 - 1.03)	0.636152	1.18 (1.13 - 1.22)	2.05E-16	325517
	Entire	1.01 (0.95 - 1.06)	0.7951523	1.01 (0.95 - 1.06)	6.04E-14	222532

JHEP Reports

CTAT methods

Tables for a "Complete, Transparent, Accurate and Timely account" (CTAT) are now mandatory for all revised submissions. The aim is to enhance the reproducibility of methods.

- Only include the parts relevant to your study
- Refer to the CTAT in the main text as 'Supplementary CTAT Table'
- Do not add subheadings
- Add as many rows as needed to include all information
- Only include one item per row

If the CTAT form is not relevant to your study, please outline the reasons why:

1.1 Antibodies

Name	Citation	Supplier	Cat no.	Clone no.

1.2 Cell lines

Name	Citation	Supplier	Cat no.	Passage no.	Authentication test method

1.3 Organisms

Name	Citation	Supplier	Strain	Sex	Age	Overall n number

1.4 Sequence based reagents

Name	Sequence	Supplier

1.5 Biological samples

Description	Source	Identifier

1.6 Deposited data

Name of repository	Identifier	Link

1.7 Software

Software name	Manufacturer	Version
**RStudio**	**The R Foundation for Statistical Computing**	**4.1.3**

1.8 Other (*e.g.* drugs, proteins, vectors etc.)

1.9 Please provide the details of the corresponding methods author for the manuscript:

Brian Ho

Wolfson Centre for Personalised Medicine,
Institute of Translational Medicine,
University of Liverpool,
Liverpool, UK

b.ho@liverpool.ac.uk

2.0 Please confirm for randomised controlled trials all versions of the clinical protocol are included in the submission. These will be published online as supplementary information.
