# Supplementary material for: Role of fatty liver index in risk-stratifying comorbid disease outcomes in non-alcoholic fatty liver disease
Source: JHEP Rep. 2023 Aug 24;5(12):100896. doi: 10.1016/j.jhepr.2023.100896 (PMC10624587; doi:10.1016/j.jhepr.2023.100896)
Supplement: Multimedia component 4 [file mmc4.pdf]

Role of fatty liver index in risk-stratifying comorbid disease outcomes in non-alcoholic fatty liver disease

Authors
Brian Ho, Andrew Thompson, Andrea L Jorgensen, Munir Pirmohamed

Correspondence
b.ho@liverpool.ac.uk (B. Ho).

Graphical abstract

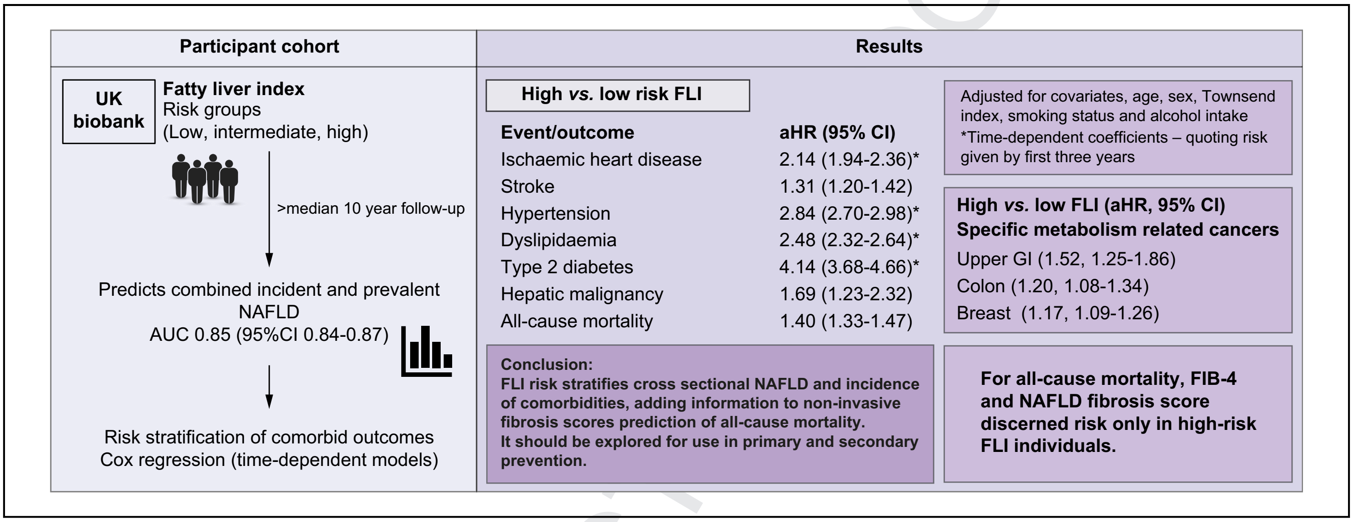

Highlights

- FLI calculated at middle to late age (first attendance to UK Biobank) identifies risk for NAFLD cross-sectionally.
- FLI stratifies incident risk of cardiometabolic diseases, hepatic malignancy, specific metabolism-related malignancies, and all-cause mortality.
- FLI and either NAFLD fibrosis score or FIB4 index independently predict risk of all-cause mortality.
- FLI could be a tool considered for one-off screening of NAFLD and its associated comorbidities.

Impact and implications

Our analysis using the UK Biobank study shows the potential of the fatty liver index as a risk stratification tool for identifying the risk of developing NAFLD, ischaemic heart disease, ischaemic stroke, type 2 diabetes mellitus, hypertension, hyperlipidaemia, hepatic malignancy, specific metabolism-related malignancies and all-cause mortality. These results suggest that the fatty liver index should be considered as a non-invasive steatosis score that may help guide primary prevention strategies for NAFLD and related outcomes.

UNCORRECTED PROOF

https://doi.org/10.1016/j.jhepr.2023.100896

Role of fatty liver index in risk-stratifying comorbid disease outcomes in non-alcoholic fatty liver disease

Brian Ho,[1,2,*] Andrew Thompson,[1,2,3] Andrea L Jorgensen,[4] Munir Pirmohamed[1,2]

[1]Wolfson Centre for Personalised Medicine, Institute of Translational Medicine, University of Liverpool, Liverpool, UK; [2]Department of Pharmacology and Therapeutics, Institute of Translational Medicine, University of Liverpool, Liverpool, UK; [3]Health Analytics, Lane Clark & Peacock LLP, London, UK; [4]Biostatistics, Institute of Translational Medicine, University of Liverpool, Liverpool, UK

JHEP Reports **2023.** https://doi.org/10.1016/j.jhepr.2023.100896

Background & Aims: Population screening for non-alcoholic fatty liver disease (NAFLD) and associated comorbidities remains an unaddressed clinical need. We aimed to assess the utility of the fatty liver index (FLI) for risk stratification of NAFLD and related comorbidities using the UK Biobank.
Methods: Electronic health records and liver MRI-proton density fat fraction (PDFF) were used to define NAFLD cases. FLI was calculated and individuals with high alcohol intake and other liver diseases were excluded. Using listwise deletion analysis, the area under receiver-operating characteristic curve (AUROC) of FLI for NAFLD risk was determined. Thereafter, time-dependent covariate-adjusted Cox regression models were used to estimate FLI's risk stratification potential for comorbidities of interest.
Results: FLI was derived for 327,800 individuals with a median age of 58 (IQR 51.5-64.5), of whom 59.8% were females. Using Perspectum Diagnostics and AMRA protocols as references, FLI identified the risk of NAFLD with AUROCs (95% CI, n) of 0.858 (0.848-0.867, n = 7,566) and 0.851 (0.844-0.856, n = 10,777), respectively. Intermediate and high-risk FLI was associated with increased cardiometabolic and malignant disease. In the first 3 years, high-risk FLI conferred an increased risk (adjusted hazard ratio, 95% CI) of ischaemic heart disease (2.14, 1.94-2.36), hypertension (2.84, 2.70-2.98), type 2 diabetes mellitus (4.55, 4.04-5.12), dyslipidaemia (2.48, 2.32-2.64), ischaemic stroke (1.31, 1.20-1.42) and hepatic malignancy (1.69, 1.23-2.30). FLI was not associated with risk of extrahepatic malignancy but was associated with a higher risk of specific cancers (colon, upper gastrointestinal and breast). All-cause mortality was similarly stratified by FLI, independently of non-invasive fibrosis scores.
Conclusions: FLI identifies NAFLD and holds potential for the risk stratification of cardiometabolic and malignant disease outcomes (including some extrahepatic malignancies), as well as all-cause mortality. Its use in population screening for primary and secondary prevention of NAFLD should be considered.
Impact and implications: Our analysis using the UK Biobank study shows the potential of the fatty liver index as a risk stratification tool for identifying the risk of developing NAFLD, ischaemic heart disease, ischaemic stroke, type 2 diabetes mellitus, hypertension, hyperlipidaemia, hepatic malignancy, specific metabolism-related malignancies and all-cause mortality. These results suggest that the fatty liver index should be considered as a non-invasive steatosis score that may help guide primary prevention strategies for NAFLD and related outcomes.
© 2023 The Authors. Published by Elsevier B.V. on behalf of European Association for the Study of the Liver (EASL). This is an open access article under the CC BY license (http://creativecommons.org/licenses/by/4.0/).

Introduction

Non-alcoholic fatty liver disease (NAFLD) is estimated to affect 25% of the world's population and is predicted to become the most prevalent liver disease globally, contributing to healthcare cost and burden.[1,2] NAFLD is independently associated with cardiovascular and malignant outcomes, in addition to increasing the risk of progressive liver disease, contributing to morbidity and mortality.[3–6] As such, NAFLD can be considered as part of a disease syndrome encompassing multiple disease-related comorbidities. This syndrome or "comorbidome" may largely be preventable by addressing metabolic risk factors, but NAFLD-specific primary prevention measures are lacking. In the community, NAFLD is often detected through abnormal liver function tests, such as aminotransferase levels, which are not sensitive and can be normal in advanced disease.[7,8] Abdominal ultrasound, often conducted for other reasons, is another modality which NAFLD is identified, but it performs poorly at detecting mild steatosis and is operator-dependent.[9] As such, identification and clinical stratification of NAFLD risk in the community remains suboptimal, with most individuals with disease being undiagnosed and patient risk factors unaddressed.[10] This is in contrast to cardiovascular diseases, where primary prevention is widely practiced by addressing risk factors, such as hypertension and dyslipidaemia.

There is interest in identifying chronic liver disease, including NAFLD, at the population level.[11] To achieve this, automated

Keywords: NAFLD; Fatty Liver Index; Comorbidities; Risk Stratification; non-invasive tests.
Received 4 January 2023; received in revised form 3 July 2023; accepted 3 August 2023; available online 24 August 2023
* Corresponding author. Address: The University of Liverpool, The Wolfson Centre for Personalised Medicine, Block A: Waterhouse Buildings, 1-5 Brownlow Street, Liverpool, L69 3GL, United Kingdom.
E-mail address: b.ho@liverpool.ac.uk (B. Ho).

integrated reflex testing systems have been proposed in the primary care setting – these have been shown to be effective, practical and cost effective.[12–14] For the identification of NAFLD specifically, a variety of non-invasive clinical tests have also been devised to assess the risk of the presence of steatosis – one such test is the fatty liver index (FLI).[15] FLI uses BMI, waist circumference, gamma-glutamyltransferase (GGT) and total triglyceride levels for its calculation. The components are simple measures commonly available in primary care and not dependent on co-existent disease.[16] Since comorbidities of NAFLD can develop before or after the development of liver disease, FLI has been suggested as a promising tool to identify NAFLD-prone individuals and their risk of associated comorbid conditions in primary care, enabling stratification for primary or secondary prevention. However, FLI has not been widely implemented in clinical practice, and indeed there are no national screening programmes to identify individuals with NAFLD, with significant variation in practice between different localities.[17]

In this study, we have utilised the UK Biobank (UKB) to explore the utility of FLI to identify individuals at risk of NAFLD, and its ability to risk stratify incident comorbidities across multiple cardiovascular, metabolic, and malignant outcomes. Further, we mimicked current NAFLD risk stratification strategies by examining the risk of mortality predicted by FLI alone and in combination with two non-invasive fibrosis scores, fibrosis-4 index (FIB4) and NAFLD fibrosis score (NFS).

Materials and methods

UK biobank study

The UKB is a prospective study that recruited ~500,000 individuals aged between 40-69 years in the UK between 2006 and 2010.[18] The study collected demographic, behavioural, physical, biological sampling, clinical bedside measurement and imaging data from its participants. There is also linked data to national inpatient and primary care records.[19] Ethics approval was obtained from the UK North-West Multi-Centre Ethics Committee (ref: 16/NW/0274) and informed consent was obtained from each participant for data usage in research purposes. The following data analysis stems from UKB research application ID 54764.

Study population

FLI was calculated for every individual in the UKB from measurements collected at the initial visit as published by Bedogni *et al.*[16] All participants with available FLI were included in the study. Individuals with FLI scores of <30, 30-59 and ≥60 were classified as having low, intermediate and high risk of steatosis, respectively. We calculated the weekly alcohol intake of participants as described previously by our group and excluded males and females who drank >21 and 14 weekly standard UK alcohol units, respectively.[20] Further exclusion criteria included other causes of liver disease, including inherited, viral and alcohol, defined by ICD-9 and ICD-10 codes from linked hospital inpatient data (Tables S1 and S2) or positive serum virology results. NFS and FIB4, two non-invasive fibrosis scores, were also calculated and used to stratify patients into low, intermediate and high-risk groups as previously reported.[21,22]

NAFLD case/control definitions

MRI imaging was performed on a Siemens 1.5 Tesla MAGNETOM Aera scanner (Siemens Healthineers, Erlangen, Germany). The analysis protocol for liver MRI-derived proton density fat fraction (PDFF) was developed by two separate companies, Perspectum Diagnostics and AMRA.[23,24] At time of data extraction, there were 4,614 and 9,892 participants with available MRI-PDFF data from each company's protocol, respectively. We arbitrarily derived two case-control cohorts with overlap using each MRI protocol, whereby participants with ≥5% PDFF were defined as having NAFLD. To increase our sample size, ICD9/ICD10 codes and primary care data were used to identify additional NAFLD cases (Fig. S1, Table S3). Healthy individuals were defined by MRI-PDFF <5%.

Exposure, covariate and outcome variables

Covariates of interest for time-to-event modelling were selected based on previous literature for known associations with NAFLD: age, gender, Townsend deprivation index, smoking status, alcohol intake and type 2 diabetes mellitus (T2DM). We investigated ischaemic heart disease, ischaemic stroke, T2DM, hypertension, hyperlipidaemia, hepatic malignancy, extrahepatic malignancies, and all-cause mortality as events of interest. For extrahepatic malignancies, an additional sub-analysis specific to metabolism-associated cancers, namely colon (including rectal), upper gastrointestinal (GI: oesophageal and stomach) and breast cancer, was performed.Waist-hip ratio and BMI were not used as covariates due to inclusion of BMI and waist circumference in the calculation of FLI. All disease covariates and events of interest were identified through available linked clinical data registries (supplementary methods). The earliest date of recorded disease was considered the time of diagnosis. Exposures of interest are FLI classification for all outcomes and additionally non-invasive fibrosis scores when examining all-cause mortality. Participants who withdrew from the study or died were censored. The analysis was right censored using an arbitrary study end date of 31 December 2019, and the start date for each participant was defined at the UKB's initial visit date (time of FLI calculation).

Statistical analysis

The methodology used for statistical analysis is detailed in the supplementary methods. Briefly, descriptive statistics are presented as median and interquartile ranges and proportions for continuous and categorical descriptors, respectively. Performance of FLI in identifying combined incident and prevalent NAFLD was first assessed using receiver-operating characteristic (ROC) analyses, while calculated sensitivities, specificities, positive predictive and negative predictive values are presented for previously published cut-offs of FLI, against defined NAFLD cases and controls. ROC analysis was further performed for two other non-invasive steatosis tests, lipid accumulation product and hepatic steatosis index, calculated as previously published.[25,26] Incidence disease rates were calculated for each FLI risk category and for misclassified individuals by FLI for NAFLD. Subsequently, Cox proportional hazard models were fitted to assess incidental disease risk stratification by FLI classification. Univariate models were first fitted to select variables to be included in subsequent multivariate models, with p <0.10 used as a loose selection threshold. Model assumptions were tested, and our models were modified to account for any violations of proportional hazards and non-linearity. Two multivariate models were fitted for each outcome. The first model included age and sex, while the second one additionally included all covariates selected from the univariate analysis. Incidental all-cause mortality was investigated in a similar manner through Cox regression modelling. In addition, modelling of FLI and one of the non-invasive fibrosis scores (NFS or FIB4) were investigated with and

without adjustment for covariates. In all time-to-event analyses, a listwise deletion dataset was used. *p* values were adjusted by Bonferroni-correction for multiplicity of tests and usefulness of model fitting with FLI and/or fibrosis scores were tested with likelihood ratio tests where appropriate. Additional sensitivity analyses were performed for significance of components of FLI (BMI, waist circumference, triglyceride and GGT levels), and missing covariate data. Analyses was performed on R version 4.0.2 using package pROC, ggplot2, forestplot, survival and survminer.

Results

FLI analysis cohort

From the initial UKB cohort of 502,460 participants, 327,800 had all data available for the calculation of FLI after implementing our exclusion criteria. Participant characteristics are described in Table 1. In contrast to individuals in the low-risk FLI, those with higher FLI risk tended to be older, male, smokers, and had higher BMI, waist-hip ratios, as well as greater levels of socioeconomic deprivation. Biochemistry results demonstrated that higher FLI risk was reflective of individuals with increased risk of metabolic and hepatic disease. The median hepatic MRI-PDFF values increased from low- to high-risk FLI.

Identification of NAFLD risk by FLI

The performance of FLI to identify combined incident and prevalent NAFLD in the UKB was first assessed against two NAFLD case-control definitions, which differ by the proprietary protocol used to measure MRI liver fat fraction. Using the Perspectum Diagnostics' protocol and clinical health records coding to define NAFLD in the UKB enabled an analysis of 7,656 individuals with available FLI data. Partitioning at a lower FLI cut-off (>30) resulted in a sensitivity, specificity, positive predictive value (PPV) and negative predictive value (NPV) of 93.04%, 53.46%, 75.03% and 83.63%, respectively. Partitioning at the higher cut-off (>60) resulted in a sensitivity, specificity, PPV and NPV of 75.28%, 80.55%, 56.04% and 90.82% respectively (Fig. S2). Similarly, using the alternative MRI protocol derived by AMRA and clinical coding enabled an analysis of 10,777 participants with FLI data. Partitioning at the lower FLI cut-off resulted in a sensitivity, specificity, PPV and NPV of 92.49%, 53.38%, 84.15% and 72.65%, respectively. At the higher cut-off, the values were 73.39%, 80.59%, 69.34% and 83.51%, respectively. On ROC analyses, the AUROC was 0.858 (95% CI 0.848-0.867) and 0.851 (95% CI 0.844-0.856) for these two definitions of NAFLD (Fig. 1). With FLI, 24.3% and 15.6% would be classified as low risk despite having NAFLD, whereas 9.4% and 16.8% would be classified as high risk despite having a normal liver, against our two respective NAFLD definitions. This corresponds to a misclassification rate of 13.3% and 16.4%, when excluding the intermediate-risk group from the calculation. Further comparison was made with two other non-invasive steatosis scores, hepatic steatosis index and lipid accumulation product, which showed inferior ROC results compared to FLI in identifying the risk of NAFLD against these two case-control definitions (Fig. 1).

Univariate analysis of incident outcomes

We next assessed whether FLI and potential covariates may predict the selected comorbid outcomes of NAFLD. The incident rates for low- to high-risk FLI (per 100 person-years) increased from 0.31 to 0.93 for ischaemic heart disease, 0.11 to 0.20 for ischaemic stroke, 1.07 to 3.25 for hypertension, 0.56 to 1.65 for dyslipidaemia, 0.25 to 1.03 for T2DM, 0.01 to 0.02 for hepatic malignancy and 1.22 to 1.56 for extrahepatic malignancy (Figs 2, 3, Table S4). Having determined the misclassification rates of FLI, we also calculated the subgroup absolute incidence rates of individuals with NAFLD classified as low-risk FLI and healthy persons with high-risk FLI (Table S5).

Using a listwise deletion approach, over 190,000 individuals were available for univariate and downstream multivariable time-to-event analyses across disease outcomes of interest, with a median follow-up of >10 years (Fig. S3). Univariate analysis showed FLI was associated with all disease outcomes. All covariates selected on univariate analysis also passed the threshold for selection (p <0.10) in multivariate models, with some exceptions (Table S6). These were Townsend index (p = 0.69) and alcohol intake (p = 0.55) for hepatic malignancy and T2DM (p = 0.16) for extrahepatic malignancy. Similarly, for all-cause mortality, all covariates passed the selection threshold for multivariable analysis. For FIB-4 and NFS, individuals with intermediate-risk (HR [95% CI] 1.81 [1.74-1.89], $p = 2.5\times10^{-176}$ and 1.98 [1.90-2.06], $p = 2\times10^{-220}$) and high-risk (HR [95% CI] 3.51 [3.19-3.86], $p = 7.10\times10^{-148}$ and 3.77 [3.38-4.19], $p = 1.4\times10^{-131}$) were at significantly increased risk of death, respectively. Furthermore, intermediate and high FLI risk (HR [95% CI] 1.42 [1.35-1.50], $p = 1.13\times10^{-38}$ and 1.96 [1.87-2.06], $p = 5.3\times10^{-176}$) had increased risk of all-cause mortality compared to low-risk counterparts (Fig. 3 and Fig. S4, Table S7).

Multivariate analysis of FLI for incident disease outcomes

To assess the ability of FLI to stratify the incidence of selected NAFLD comorbidities, Cox proportional hazards models were fitted with covariates selected from univariate analysis. Of the disease outcomes investigated, FLI met the proportional hazards assumption required for Cox regression on examination of scaled Schoenfeld residuals for ischaemic stroke and hepatic malignancy. Given this, time-dependent coefficient modelling was employed for FLI for all other disease outcomes investigated. For the two outcomes where time-dependent models were not applied, individuals with intermediate-risk FLI had an increased incident risk of ischaemic stroke (HR [95% CI] 1.21 [1.11-1.33], $p = 2.55\times10^{-5}$), but similar risk of hepatic malignancy (HR [95% CI] 1.01 [0.70-1.45], p = 0.965) when compared to those with low-risk FLI. However, high-risk FLI risk-stratified both diseases (HR [95% CI] 1.31 [1.20-1.42], $p = 1.26\times10^{-9}$ and 1.69 [1.23-2.32], p = 0.001, respectively) (Fig. 4).

For time-dependent modelling of FLI, we arbitrarily split the follow-up period into 3-yearly intervals to allow for more refined estimation of risk by FLI during follow-up. Our analysis shows that the incident risk of ischaemic heart disease, hypertension, dyslipidaemia, and T2DM was informed by the non-invasive steatosis score, for both the intermediate- and high-risk category at all intervals of the follow-up period, but not for extrahepatic malignancy (Fig. 4, Table S8). To illustrate, an intermediate-risk FLI was associated with a 1.52-fold [95% CI 1.37-1.70] increased risk of ischaemic heart disease in the first 3 years of follow-up, which reduced to 1.31-fold [95% CI 1.17-1.47] at >9 years of follow-up, when compared to individuals with low-risk FLI. Similarly, high-risk FLI was associated with a 2.14-fold [95% CI 1.94-2.36] and 1.66-fold [95% CI 1.49-1.85] increase in risk when compared to low-risk FLI for these two periods of follow-up, respectively. There was a general trend in the reduction of risk with both intermediate- and high-risk FLI with longer periods of follow-up, except for T2DM. The reduction in estimated risk was most prominent when

Table 1. Characteristics of patient cohort.

	FLI risk						Entire cohort	
**Characteristic**	**Low (n = 124,126)**	**n**	**Intermediate (n = 86,062)**	**n**	**High (n = 177,612)**	**n**	**Total (n = 327,800)**	**n**
Female	78.62%	97,592	54.03%	46,501	44.17%	51,946	59.80%	196,039
Type 2 diabetes	6.58%	8,162	8.71%	7,495	15.96%	18,765	10.50%	34,422
Smoking status								
Never	66.44%	82,139	61.31%	52,462	54.28%	63,370	60.48%	197,971
Previous	25.72%	31,798	30.14%	25,792	35.94%	41,957	30.41%	99,547
Current	7.84%	9,696	8.55%	7,314	9.79%	11,425	8.69%	28,435
Age (years)	56 (48–62)	124,126	59 (51–64)	86,062	59 (52–64)	117,612	58 (51.5–64.5)	327,800
BMI (kg/h^2)	23.52 (21.94–25.1)	124,126	26.75 (25.27–28.4)	86,062	31.05 (28.67–34.22)	117,612	26.66 (23.7–29.63)	327,800
Waist-hip ratio	0.47 (0.44–0.49)	124,126	0.53 (0.51–0.55)	86,062	0.6 (0.56–0.64)	117,612	0.53 (0.48–0.58)	327,800
Systolic blood pressure (mmHg)	125 (115–139)	124,075	133 (122–145)	86,016	136 (125–148)	117,372	131 (119–143)	327,463
Diastolic blood pressure (mmHg)	76 (69–82)	124,075	80 (74–86)	86,017	83 (76–89)	117,373	79 (72.5–85.5)	327,465
Alcohol intake (units/week)	7.5 (4–10.5)	88,048	8.2 (4.1–12.1)	60,040	8.6 (4–13.1)	75,490	7.8 (3.8–11.8)	223,578
Townsend deprivation index	−2.33 (−3.75–0.13)	123,980	−2.26 (−3.7–0.33)	85,950	−1.84 (−3.47–1.14)	117,455	−2.15 (−4.24–−0.05)	327,385
**Blood parameters**								
Hb (g/dl)	13.6 (12.92–14.3)	120,921	14.2 (13.4–15)	83,839	14.5 (13.62–15.36)	114,417	14.03 (13.2–14.86)	319,177
WBC (x10^9 cells/L)	6.26 (5.3–7.39)	120,920	6.61 (5.66–7.77)	83,839	7.14 (6.1–8.39)	114,413	6.67 (5.56–7.78)	319,172
Platelet (x10^9 cells/L)	248.1 (214–286.6)	120,919	249.5 (214.2–288.92)	83,840	250.2 (214.3–291)	114,417	249.2 (211.85–286.55)	319,176
Creatinine (μmol/L)	65.3 (58.4–73.9)	124,109	71.4 (62.1–81.8)	86,046	73.9 (63.9–84.6)	117,602	69.6 (59.9–79.3)	327,757
Total bilirubin	7.92 (6.36–10.21)	123,690	7.91 (6.28–10.28)	85,800	7.8 (6.18–10.12)	117,110	7.87 (5.91–9.83)	326,600
ALT (U/L)	16.01 (13.01–19.98)	124,089	19.86 (15.85–25.29)	86,048	25.2 (19.22–33.99)	117,483	19.59 (13.92–25.26)	327,620
AST (U/L)	22.8 (19.8–26.3)	123,719	24 (20.9–27.9)	85,822	25.7 (22–30.7)	117,068	24.1 (20.35–27.85)	326,609
ALP (U/L)	75.9 (63.1–90.7)	124,092	82.6 (69.8–98)	86,052	86.5 (72.9–102.8)	117,580	81.4 (66.85–95.95)	327,724
GGT (U/L)	17.8 (14.4–23.2)	124,126	24.7 (19.2–33.7)	86,062	35.7 (25.9–53.3)	117,612	24.4 (14.8–34)	327,800
Alb (g/dl)	45.2 (43.52–46.91)	112,878	45.07 (43.4–46.79)	78,753	44.96 (43.22–46.71)	108,369	45.09 (43.38–46.8)	300,000
Urate (μmol/L)	250.9 (214.5–292.7)	123,936	299.3 (256.6–346)	85,923	339.8 (292.4–390.5)	117,467	293.7 (241.2–346.2)	327,326
CRP (mg/L)	0.78 (0.42–1.55)	123,851	1.37 (0.74–2.59)	85,900	2.27 (1.21–4.39)	117,301	1.34 (0.27–2.42)	327,052
HDL-C (mmol/L)	1.6 (1.37–1.85)	112,855	1.35 (1.17–1.57)	78,746	1.18 (1.02–1.38)	108,345	1.37 (1.13–1.62)	299,946
LDL-C (mmol/L)	3.39 (2.88–3.94)	123,936	3.62 (3.05–4.22)	85,931	3.6 (2.95–4.24)	117,415	3.52 (2.93–4.11)	327,282
Triglyceride (mmol/L)	1.05 (0.82–1.36)	124,126	1.54 (1.19–2.02)	86,062	2.13 (1.57–2.9)	117,612	1.48 (0.94–2.02)	327,800
Cholesterol (mmol/L)	5.56 (4.88–6.28)	124,098	5.7 (4.94–6.47)	86,041	5.63 (4.79–6.47)	117,602	5.62 (4.85–6.38)	327,741
**MRI-PDFF quantification (% steatosis)**								
Perspectum Diagnostics algorithm	1.3 (0.97–2)	1,246	2.3 (1.49–4.22)	755	4.21 (2.18–9.32)	790	1.96 (0.51–3.41)	2,791
AMRA Algorithm	1.52 (1.16–2.22)	2,636	2.52 (1.67–4.49)	1,632	4.47 (2.44–9.24)	1,682	2.2 (0.74–3.65)	5,950

Table showing characteristics of UKB participants with data available to calculate FLI and also by the index risk categories. Summary statistics are shown in median and interquartile ranges for continuous values and percentages for categorical values, with the number of available for analysis.
ALP, alkaline phosphatase; ALT, alanine aminotransferase; AST, aspartate aminotransferase; CRP, C-reactive protein; FLI, fatty liver index; GGT, gamma-glutamyltransferase; HDL-C, high-density lipoprotein-cholesterol; LDL-C, low-density lipoprotein-cholesterol; UKB, UK biobank; WBC, white blood cell count.

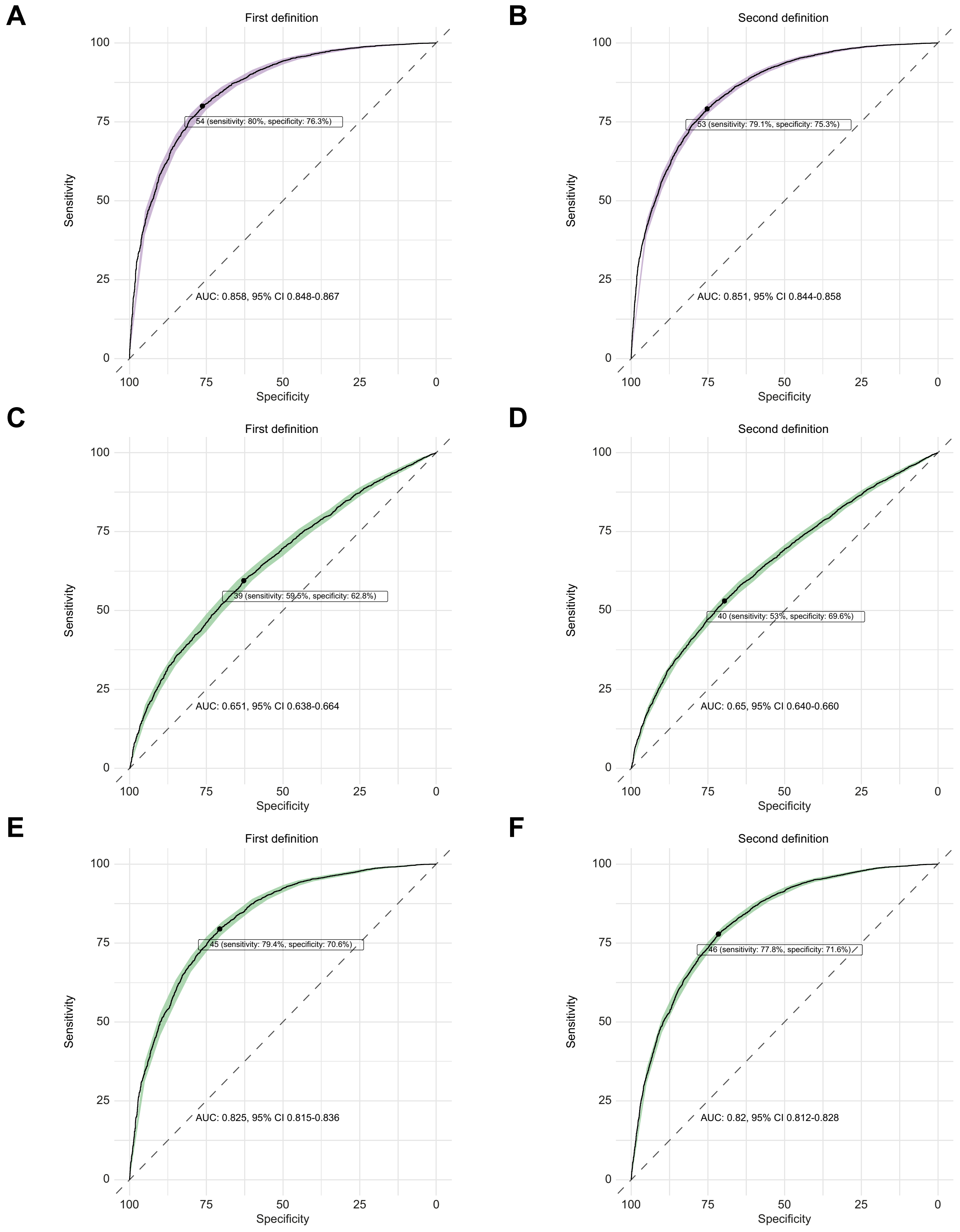

Fig. 1. Performance of non-invasive steatosis scores estimating combined prevalent and incident NAFLD risk. Receiver-operating characteristic curves of FLI (A, B), hepatic steatosis index (C, D) and lipid accumulation product (E, F) against two case-control definitions of NAFLD. First definition (left column) and second definition (right column) based on MRI results derived from Perspectum Diagnostics and AMRA, respectively. AUC and best binary cut-off shown, with sensitivity and specificity values. 95% CI were derived from bootstrapping (n = 1,000) and shown in shaded areas. FLI, fatty liver index; NAFLD, non-alcoholic fatty liver disease.

A

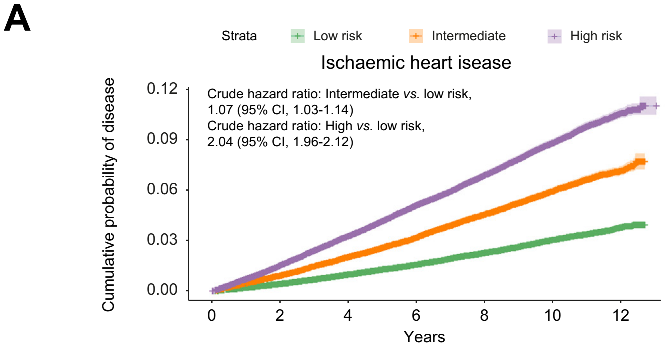

N° at risk							
Low risk	86,112	85,500	84,616	83,561	82,463	63,260	7,096
Intermediate	57,349	56,626	55,676	54,581	53,378	40,520	4,353
High risk	69,907	68,546	66,807	64,913	63,044	46,754	4,636

Cumulative n° of events							
Low risk	0	375	853	1,362	1,949	2,576	2,821
Intermediate	0	528	1,160	1,818	2,586	3,333	3,633
High risk	0	1,067	2,278	3,541	4,763	6,013	6,505

B

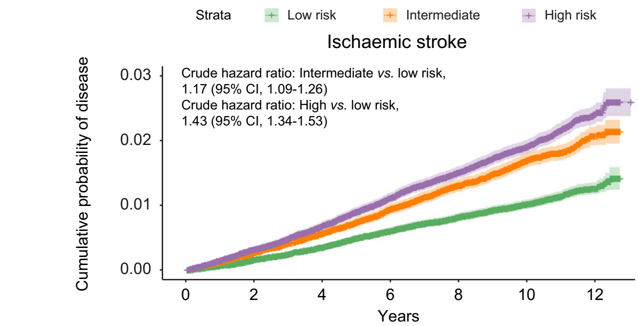

N° at risk							
Low risk	87,310	86,904	86,287	85,477	84,694	65,226	7,314
Intermediate	59,311	58,900	58,326	57,583	56,818	43,426	4,704
High risk	74,237	73,589	72,619	71,446	70,228	52,766	5,255

Cumulative n° of events							
Low risk	0	141	308	526	711	875	965
Intermediate	0	158	338	555	767	981	1,058
High risk	0	239	508	820	1,104	1,376	1,521

C

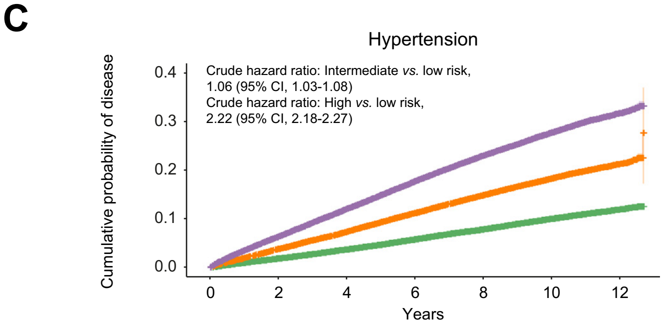

N° at risk							
Low risk	82,193	80,377	78,492	76,343	74,255	56,100	63,45
Intermediate	52,403	50,190	48,085	45,741	43,543	32,274	3,503
High risk	59,493	55,435	51,673	47,929	44,424	31,849	3,269

Cumulative n° of events							
Low risk	0	1,592	3,113	4,804	6,481	8,170	8,823
Intermediate	0	2,032	3,875	5,880	7,773	9,472	10,131
High risk	0	3,813	7,196	10,518	13,606	16,251	17,235

D

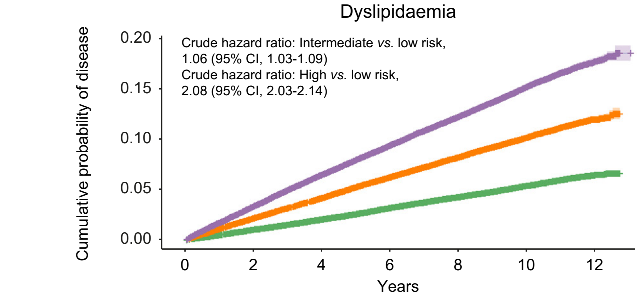

N° at risk							
Low risk	85,227	84,097	82,826	81,322	79,898	61,186	6,961
Intermediate	56,262	54,826	53,368	51,787	50,262	38,028	4,201
High risk	67,913	65,321	62,666	60,099	57,578	42,411	4,361

Cumulative n° of events							
Low risk	0	882	1,742	2,706	3,611	4,515	4,899
Intermediate	0	1,224	2,340	3,499	4,583	5,624	6,051
High risk	0	2,274	4,398	6,351	8,227	10,107	10,801

E

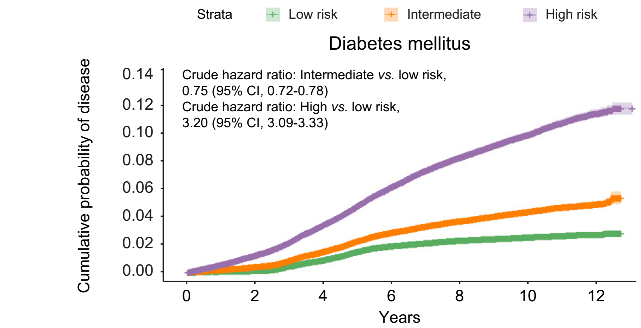

N° at risk							
Low risk	82,016	81,672	80,626	79,265	78,391	60,962	6,865
Intermediate	54,924	54,478	53,533	52,317	51,379	39,672	4,350
High risk	64,469	63,378	61,421	59,052	57,086	43,065	4,376

Cumulative n° of events							
Low risk	0	103	730	1,546	1,867	2,047	2,121
Intermediate	0	221	818	1,561	2,002	2,353	2,480
High risk	0	776	2,191	3,924	5,254	6,235	6,653

Fig. 2. FLI category and cumulative incidence of NAFLD comorbid outcomes. Shown are Kaplan-Meier curves of disease outcomes of interest for low-, intermediate- and high-risk FLI, as defined by scores <30, 30-59 and ≥60, respectively. Incident (A) ischaemic heart disease, (B) ischaemic stroke, (C) hypertension, (D) dyslipidaemia and (E) diabetes mellitus. 95% CIs are plotted (shaded area) with + signs indicating data censoring. Crude hazard ratios are unadjusted derived from univariate Cox regression. FLI, fatty liver index; NAFLD, non-alcoholic fatty liver disease.

A

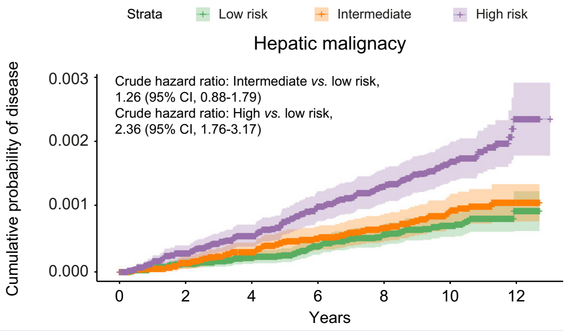

N° at risk							
Low risk	87,716	87,437	86,977	86,350	85,710	66,122	7,403
Intermediate	59,762	59,501	59,085	58,533	57,930	44,420	4,813
High risk	75,031	74,594	73,849	72,923	71,929	54,210	5,399

Cumulative n° of events							
Low risk	0	11	19	34	49	61	67
Intermediate	0	8	18	30	39	53	57
High risk	0	21	41	74	96	121	133

B

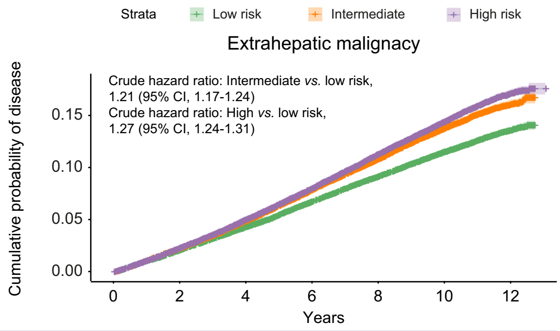

N° at risk							
Low risk	79,256	77,471	75,537	73,354	71,251	53,667	5,828
Intermediate	53,902	52,574	50,998	49,179	47,368	35,291	3,745
High risk	68,153	66,390	64,250	61,830	59,224	43,402	4,281

Cumulative n° of events							
Low risk	0	1,671	3,433	5,362	7,251	9,043	9,722
Intermediate	0	1,219	2,638	4,236	5,811	7,325	7,864
High risk	0	1,575	3,424	5,446	7,649	9,650	10,412

C

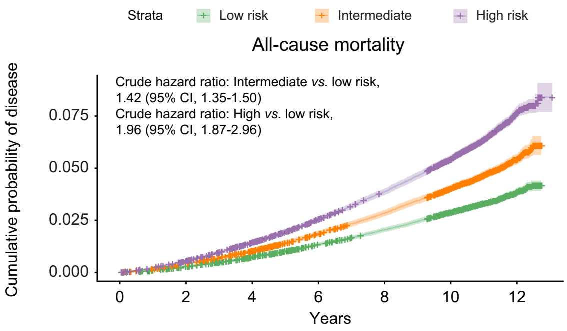

N° at risk							
Low risk	87,720	87,446	86,986	86,364	85,730	66,137	7,406
Intermediate	59,766	59,509	59,095	58,542	57,940	44,436	4,814
High risk	75,046	74,615	73,880	72,962	71,961	54,237	5,403

Cumulative n° of events							
Low risk	0	244	635	1,162	1,778	2,490	2,813
Intermediate	0	241	608	1,099	1,686	2,367	2,694
High risk	0	399	1,070	1,900	2,882	4,021	4,635

Fig. 3. FLI category and cumulative incidence of hepatic and extrahepatic malignancy. Shown are Kaplan-Meier curves for incident (A) hepatic malignancy (B) extrahepatic malignancy and (C) All-cause mortality for low-, intermediate- and high-risk FLI, as defined by scores <30, 30-59 and ≥60, respectively. 95% CIs are plotted for general interpretation (shaded area) with + signs indicating data censoring. Crude hazard ratios are derived from univariate Cox. FLI, fatty liver index.

comparing incident risk of hypertension and dyslipidaemia at up to 3 years with >9 years of follow-up by FLI risk: for intermediate-risk FLI (HR [95% CI] 1.74 [1.64-1.83] *vs.* 1.48 [1.38-1.59] and 1.70 [1.58-1.83 *vs.* 1.40 [1.28-1.54], respectively); and for high-risk FLI (HR [95% CI] 2.84 [2.7-2.98] *vs.* 2.36 [2.21-2.53] and 2.48 [2.32-2.64] *vs.* 2.05 [1.89-2.23], respectively).

In risk-stratifying T2DM, our analysis showed a bimodal pattern of fold-change in the incident risk given by FLI, with higher hazard ratios during the first 3 years and after 6 years of follow-up. When compared to individuals with low-risk FLI (HR, [95% CI]), the intermediate-risk FLI group had 1.83-fold [1.59-2.10], 1.36-fold [1.25-1.48], 2.23-fold [1.97-2.52] and 2.76-fold [2.78-3.34] and the high-risk FLI group had 4.55-fold [4.04-5.12], 2.69-fold [2.50-2.88], 5.69-fold [5.11-6.34] and 7.05-fold [5.95-8.34] higher risk of developing diabetes at 0-3, 4-6, 7-9 and >9 years of follow-up, respectively.

FLI risk stratification of metabolism-related cancer

As the value of FLI risk stratification was minimal for overall extrahepatic malignancy, we surmised that non-metabolism-related cancer risk is poorly captured by the components of FLI, which correlate strongly with metabolic disease states, and may therefore dilute estimates of metabolism-related cancers. Additional analysis was therefore performed for selected cancers, which have a known association to metabolic syndrome: colorectal, upper GI (oesophageal and stomach) and breast cancer (Fig. 5). High-risk FLI conferred an increased risk of developing colorectal, upper GI and breast cancer (HR [95% CI] 1.2 [1.08-1.34], 1.52 [1.25-1.86] and 1.17 [1.09-1.26], respectively), when compared to low-risk FLI in the covariate-adjusted model. An intermediate-risk FLI was associated with a 1.09-fold [1.01-1.17] higher risk of breast cancer but not with an increased risk of the luminal GI cancers tested.

Event	Model	Term	Follow-up period	HR (95% CI)	*p* value
Ischaemic heart disease	Age-sex adjusted	FLI intermediate *vs.* low risk	0-3 years	1.52 (1.37-1.7)	3.42e-218
			4-6 years	1.4 (1.27-1.55)	
			7-9 years	1.47 (1.35-1.61)	
			>9 years	1.33 (1.19-1.49)	
		FLI high *vs.* low risk	0-3 years	2.36 (2.14-2.6)	
			4-6 years	2.13 (1.95-2.33)	
			7-9 years	1.94 (1.78-2.11)	
			>9 years	1.79 (1.61-1.99)	
	Covariates adjusted	FLI intermediate *vs.* low risk	0-3 years	1.47 (1.32-1.63)	1.59e-163
			4-6 years	1.38 (1.25-1.52)	
			7-9 years	1.45 (1.33-1.59)	
			>9 years	1.31 (1.17-1.47)	
		FLI high *vs.* low risk	0-3 years	2.14 (1.94-2.36)	
			4-6 years	1.97 (1.8-2.15)	
			7-9 years	1.8 (1.65-1.96)	
			>9 years	1.66 (1.49-1.85)	
Stroke	Age-sex adjusted	FLI intermediate *vs.* low risk	Full	1.23 (1.13-1.35)	1.75e-14
		FLI high *vs.* low risk	Full	1.41 (1.29-1.53)	
	Covariates adjusted	FLI intermediate *vs.* low risk	Full	1.21 (1.11-1.33)	4.92e-09
		FLI high *vs.* low risk	Full	1.31 (1.2-1.42)	
Hypertension	Age-sex adjusted	FLI intermediate *vs.* low risk	0-3 years	1.76 (1.67-1.86)	<2.20e-16
			4-6 years	1.66 (1.57-1.75)	
			7-9 years	1.61 (1.53-1.7)	
			>9 years	1.5 (1.39-1.61)	
		FLI high *vs.* low risk	0-3 years	3.01 (2.86-3.16)	
			4-6 years	2.76 (2.63-2.9)	
			7-9 years	2.55 (2.43-2.68)	
			>9 years	2.5 (2.34-2.67)	
	Covariates adjusted	FLI intermediate *vs.* low risk	0-3 years	1.74 (1.64-1.83)	<2.20e-16
			4-6 years	1.63 (1.55-1.72)	
			7-9 years	1.59 (1.51-1.68)	
			>9 years	1.48 (1.38-1.59)	
		FLI high *vs.* low risk	0-3 years	2.84 (2.7-2.98)	
			4-6 years	2.61 (2.48-2.74)	
			7-9 years	2.42 (2.3-2.55)	
			>9 years	2.36 (2.21-2.53)	
Dyslipidaemia	Age-sex adjusted	FLI intermediate *vs.* low risk	0-3 years	1.74 (1.62-1.87)	<2.20e-16
			4-6 years	1.55 (1.45-1.67)	
			7-9 years	1.59 (1.48-1.71)	
			>9 years	1.44 (1.31-1.58)	
		FLI high *vs.* low risk	0-3 years	2.72 (2.55-2.9)	
			4-6 years	2.3 (2.16-2.46)	
			7-9 years	2.38 (2.23-2.55)	
			>9 years	2.29 (2.11-2.49)	
	Covariates adjusted	FLI intermediate *vs.* low risk	0-3 years	1.7 (1.58-1.83)	<2.20e-16
			4-6 years	1.52 (1.41-1.63)	
			7-9 years	1.55 (1.44-1.67)	
			>9 years	1.4 (1.28-1.54)	
		FLI high *vs.* low risk	0-3 years	2.48 (2.32-2.64)	
			4-6 years	2.09 (1.96-2.23)	
			7-9 years	2.15 (2.01-2.3)	
			>9 years	2.05 (1.89-2.23)	
Diabetes mellitus	Age-sex adjusted	FLI intermediate *vs.* low risk	0-3 years	1.68 (1.46-1.93)	<2.20e-16
			4-6 years	1.3 (1.19-1.41)	
			7-9 years	2.03 (1.79-2.3)	
			>9 years	2.49 (2.06-3.02)	
		FLI high *vs.* low risk	0-3 years	4.37 (3.88-4.91)	
			4-6 years	2.6 (2.42-2.79)	
			7-9 years	5.44 (4.89-6.06)	
			>9 years	6.76 (5.72-8)	
	Covariates adjusted	FLI intermediate *vs.* low risk	0-3 years	1.65 (1.43-1.89)	<2.20e-16
			4-6 years	1.28 (1.18-1.39)	
			7-9 years	2.01 (1.77-2.27)	
			>9 years	2.45 (2.02-2.97)	
		FLI high *vs.* low risk	0-3 years	4.14 (3.68-4.66)	
			4-6 years	2.53 (2.35-2.71)	
			7-9 years	5.2 (4.66-5.79)	
			>9 years	6.36 (5.37-7.53)	
Hepatic malignancy	Age-sex adjusted	FLI intermediate *vs.* low risk	Full	1.04 (0.723-1.49)	3.2e-06
		FLI high *vs.* low risk	Full	1.96 (1.43-2.67)	
	Covariates adjusted	FLI intermediate *vs.* low risk	Full	1.01 (0.703-1.45)	0.000451
		FLI high *vs.* low risk	Full	1.69 (1.23-2.32)	
Extrahepatic malignancy	Age-sex adjusted	FLI intermediate *vs.* low risk	0-3 years	0.912 (0.858-0.97)	0.0001325
			4-6 years	1.05 (0.988-1.11)	
			7-9 years	1.04 (0.984-1.1)	
			>9 years	0.951 (0.881-1.03)	
		FLI high *vs.* low risk	0-3 years	0.935 (0.881-0.991)	
			4-6 years	1.05 (0.99-1.1)	
			7-9 years	1.1 (1.04-1.16)	
			>9 years	1.04 (0.964-1.12)	
	Covariates adjusted	FLI intermediate *vs.* low risk	0-3 years	0.91 (0.856-0.967)	0.000277
			4-6 years	1.04 (0.986-1.1)	
			7-9 years	1.04 (0.981-1.1)	
			>9 years	0.948 (0.878-1.02)	
		FLI high *vs.* low risk	0-3 years	0.929 (0.876-0.985)	
			4-6 years	1.04 (0.984-1.1)	
			7-9 years	1.1 (1.04-1.16)	
			>9 years	1.03 (0.956-1.11)	
All-cause mortality	Age-sex adjusted	FLI intermediate *vs.* low risk	Full	1.04 (0.986-1.1)	7.55e-51
		FLI high *vs.* low risk	Full	1.4 (1.33-1.47)	
	Covariates adjusted	FLI intermediate *vs.* low risk	Full	1 (0.951-1.06)	3.84e-17
		FLI high *vs.* low risk	Full	1.2 (1.14-1.27)	

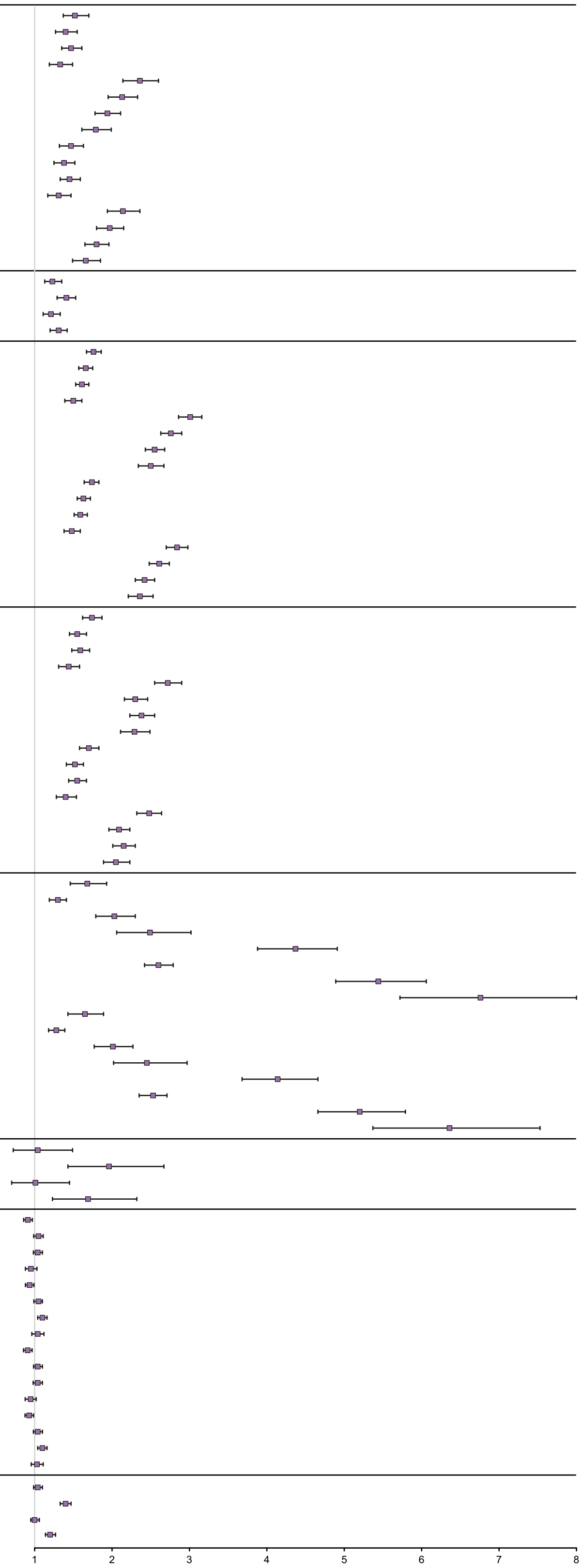

Fig. 4. FLI risk stratification of incident comorbidities. Forest plot shows the HR estimates and 95% CIs comparing intermediate- and high-risk FLI with low-risk individuals in multivariable Cox regression analysis, adjusted for covariates selected from univariate regression. The *p* value in the diagram shows log-likelihood ratio test between models with and without FLI as a variable in the regression, assessing the value of FLI in risk prediction. FLI, fatty liver index; HR, hazard ratio.

A

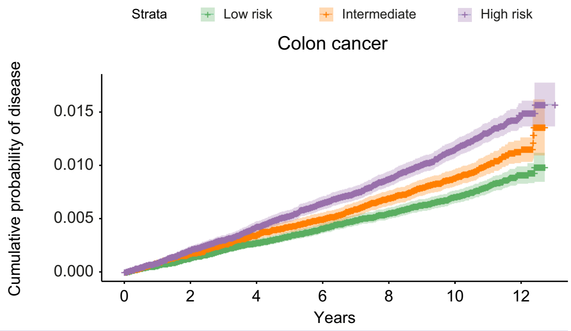

N° at risk							
Low risk	87,339	86,964	86,408	85,711	85,013	65,534	7,321
Intermediate	59,364	59,025	58,547	57,955	57,286	43,869	4,729
High risk	74,446	73,901	73,074	72,059	70,980	53,425	5,318

Cumulative n° of events							
Low risk	0	113	240	356	477	603	672
Intermediate	0	104	205	292	407	511	571
High risk	0	154	313	475	638	828	917

B

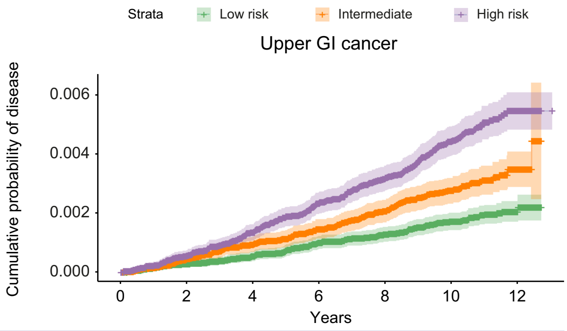

N° at risk							
Low risk	87,654	87,371	86,902	86,266	85,635	66,054	7,396
Intermediate	59,719	59,451	59,030	58,463	57,851	44,365	4,801
High risk	75,007	74,549	73,796	72,854	71,837	54,112	5,385

Cumulative n° of events							
Low risk	0	26	48	88	111	148	161
Intermediate	0	26	56	87	122	161	178
High risk	0	42	101	174	234	322	353

C

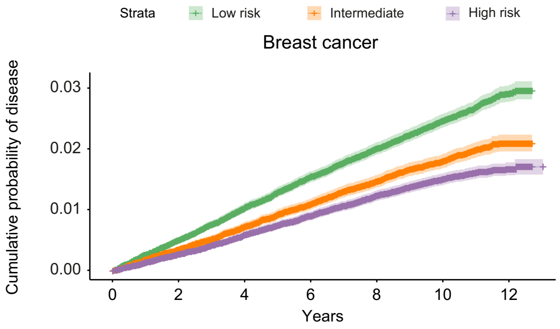

N° at risk							
Low risk	85,354	84,693	83,848	82,865	81,920	62,987	7,041
Intermediate	58,406	57,967	57,379	56,650	55,892	42,768	4,636
High risk	73,826	73,223	72,315	71,232	70,044	52,692	5,270

Cumulative n° of events							
Low risk	0	425	873	1,306	1,694	2,062	2,217
Intermediate	0	202	416	632	847	1,027	1,105
High risk	0	201	431	656	894	1,075	1,131

D

Event	Model	Term	Follow-up period	HR (95% CI)	*p* value
Colon cancer	Age-sex adjusted	FLI intermediate *vs.* low risk	Full	0.99 (0.883-1.11)	3.84e-06
		FLI high *vs.* low risk	Full	1.25 (1.12-1.39)	
	Covariates adjusted	FLI intermediate *vs.* low risk	Full	0.98 (0.874-1.1)	0.000124
		FLI high *vs.* low risk	Full	1.2 (1.08-1.34)	
Breast cancer	Age-sex adjusted	FLI intermediate *vs.* low risk	Full	1.08 (1.01-1.16)	0.000294
		FLI high *vs.* low risk	Full	1.16 (1.08-1.25)	
	Covariates adjusted	FLI intermediate *vs.* low risk	Full	1.09 (1.01-1.17)	9.68e-05
		FLI high *vs.* low risk	Full	1.17 (1.09-1.26)	
Upper GI cancer	Age-sex adjusted	FLI intermediate *vs.* low risk	Full	1.1 (0.887-1.37)	2.36e-07
		FLI high *vs.* low risk	Full	1.62 (1.33-1.97)	
	Covariates adjusted	FLI intermediate *vs.* low risk	Full	1.09 (0.879-1.36)	1.22e-05
		FLI high *vs.* low risk	Full	1.52 (1.25-1.86)	

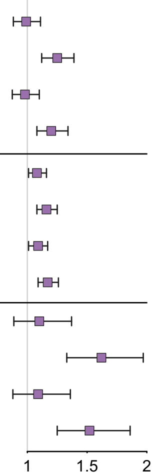

Fig. 5. FLI risk stratification of selected incident metabolism-related cancers. Shown are Kaplan-Meier curves for (A) colon, (B) breast and (C) upper GI cancer (oesophageal and gastric) for low-, intermediate- and high-risk FLI, as defined by scores of <30, 30-59 and ≥60. 95% CIs are plotted for general interpretation (shaded area) with + signs indicating data censoring. (D) The forest plot shows the HRs derived from Cox proportional hazards regression adjusted for selected covariates for each of these cancers. FLI, fatty liver index; HR, hazard ratio.

FLI and non-invasive fibrosis scores stratify all-cause mortality

Finally, all-cause mortality was examined in a similar manner. In the covariate-adjusted model, high-risk FLI was associated with a 1.2-fold [95% CI 1.14-1.27] increased risk of death from any cause, whereas individuals with intermediate-risk scores had a similar risk of death as those with low-risk FLI (Fig. 4). As current advanced liver disease risk assessment involves confirming the presence of fibrosis, which is independently associated with mortality, we examined whether FLI provides any independent information over non-invasive fibrosis scores (NFS or FIB4), a surrogate to the presence of fibrotic liver disease. To do this, we first fitted separate stratified Cox models by FLI risk category, which showed that high-risk NFS and FIB4 predicted an increased risk of all-cause mortality in individuals with intermediate- or high-risk, but not low-risk, FLI (Fig. 6 and Fig. S7). Incorporation of FLI with each non-invasive fibrosis score into a unified covariate-adjusted model showed that only the high-risk category of each non-invasive fibrosis score was significantly associated with a higher risk of death from all-causes. In each of these models, individuals with a high-risk FLI had a 1.2-fold [95% CI: 1.13 - 1.26] and 1.22-fold [1.16-1.28] higher risk of all-cause

A

FLI risk category	Term	Follow-up period	HR (95% CI)	*p* value
Low risk	Fibrosis score intermediate *vs.* low risk	Full	1.04 (0.95-1.14)	0.659
	Fibrosis score high *vs.* low risk	Full	1.04 (0.71-1.52)	
Intermediate risk	Fibrosis score intermediate *vs.* low risk	Full	1.01 (0.93-1.1)	0.00622
	Fibrosis score high *vs.* low risk	Full	1.56 (1.2-2.02)	
High risk	Fibrosis score intermediate *vs.* low risk	Full	1.05 (0.98-1.13)	0.0023
	Fibrosis score high *vs.* low risk	Full	1.28 (1.12-1.46)	

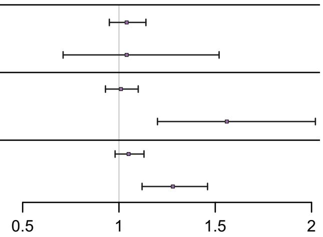

B

FLI risk category	Term	Follow-up period	HR (95% CI)	*p* value
Low risk	Fibrosis score intermediate *vs.* low risk	0-3 years	0.81 (0.65-1.01)	0.0309
		4-6 years	0.87 (0.73-1.02)	
		7-9 years	0.97 (0.84-1.12)	
		>9 years	1.05 (0.88-1.25)	
	Fibrosis score high *vs.* low risk	0-3 years	1.03 (0.61-1.75)	
		4-6 years	1.49 (1.06-2.08)	
		7-9 years	0.81 (0.56-1.19)	
		>9 years	1.12 (0.73-1.7)	
Intermediate risk	Fibrosis score intermediate *vs.* low risk	0-3 years	1.15 (0.92-1.44)	3.42e-06
		4-6 years	1.01 (0.85-1.2)	
		7-9 years	1.03 (0.89-1.19)	
		>9 years	1.05 (0.88-1.24)	
	Fibrosis score high *vs.* low risk	0-3 years	2.79 (1.85-4.21)	
		4-6 years	1.88 (1.33-2.67)	
		7-9 years	1.59 (1.15-2.18)	
		>9 years	1.28 (0.83-1.98)	
High risk	Fibrosis score intermediate *vs.* low risk	0-3 years	1.16 (0.98-1.38)	<2.2e-16
		4-6 years	0.94 (0.82-1.06)	
		7-9 years	1.03 (0.92-1.16)	
		>9 years	1.07 (0.94-1.22)	
	Fibrosis score high *vs.* low risk	0-3 years	1.91 (1.37-2.66)	
		4-6 years	1.53 (1.16-2)	
		7-9 years	1.42 (1.11-1.83)	
		>9 years	1.4 (1.03-1.89)	

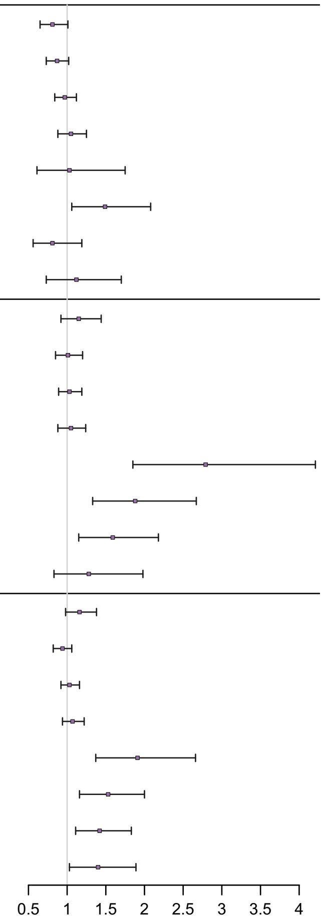

Fig. 6. Stratification of mortality with non-invasive fibrosis scores by FLI category. Forest plot shows (A) Fibrosis-4 index and (B) NAFLD fibrosis score category and risk of incident all-cause mortality by FLI risk category. Dot and whiskers show HRs and 95% CIs for adjusted Cox regression adjusted for covariates: age, sex, Townsend index, type 2 diabetes mellitus, smoking status, and alcohol intake. *p* value indicates likelihood ratio test comparing models with and without non-invasive fibrosis score as a regressor, assessing their value in predicting mortality in addition to FLI. FLI, fatty liver index; HR, hazard ratio.

mortality compared to individuals with low-risk FLI when adjusted for NFS and FIB4, respectively (Table 2 and Table S9).

Sensitivity analysis for components of FLI and missing data

Given the strong risk differences in FLI risk groups for the disease outcomes investigated, sensitivity analyses were performed to investigate whether components of FLI vary in their risk predictive capacity for these different outcomes. Using multivariate analysis to determine the role of the individual FLI components showed that ischaemic heart disease, T2DM, hypertension, dyslipidaemia and extrahepatic malignancy were informed by all components of FLI. The only outcome associated with three FLI components was all-cause mortality (waist circumference, BMI and GGT levels). Hepatic malignancy and ischaemic stroke were only significantly associated with two components of FLI (Table S10). Of note, waist circumference and BMI exhibited opposite effects towards the risk of extrahepatic malignancy. A further sensitivity analysis was performed for the main outcome analysis to investigate effects of missing data when using listwise deletion. To crudely investigate this, we ran the same time-to-event regression analysis for disease outcomes of interest with a listwise deletion cohort excluding alcohol intake as a variable, which had the largest proportion of missingness (of up to 20%). Comparing it with the main analysis cohort, little difference in risk estimates was shown for FLI, whereby estimates of risk were consistently more conservative in the final models used (Table S11).

Discussion

In one of the largest studies undertaken to date, we have demonstrated that a higher FLI from a single measurement performed in the UKB identified individuals at risk of existent and incident NAFLD and its comorbidities. A previous meta-analysis of 27,221 individuals showed that the sensitivity, specificity, PPV, and NPV for FLI were 81%, 65%, 53%, and 84%, respectively, for the lower cut-off, while the corresponding values at the higher cut-off were 44%, 90%, 67%, and 76%, respectively.[27] Our data compare favourably with these estimates, but with slightly higher sensitivity values and slightly lower specificity at both cut-off thresholds. A previous study comparing FLI with ^{1}H-magnetic resonance spectroscopy showed an AUROC of 0.79 compared with our value of 0.85, suggesting FLI has good diagnostic value.[28] However, in contrast to its use for point of care diagnosis in the previous studies, our results suggest that FLI may be capable of identifying NAFLD and associated comorbidities over a lifetime period up to middle and early old-age. This is reflected by the age group recruited in the UKB and reflects risk stratification of a one-off score between the ages of 40-69.

A misclassification rate of FLI for NAFLD is also observed from our analysis. This is expected given only a small proportion of risk is captured by the index for the complex trait disorder. As such, it is important to distinguish that our time-to-event analysis results do not reflect a direct assessment of NAFLD towards the risk of developing the disease, but rather the risk from metabolic factors associated with NAFLD development, which it shares with many other metabolic conditions. FLI thus provides a validated score to link NAFLD with incident disease outcome assessment, which has not been considered in the development of many established risk stratification tools in use, such as QRISK3 for cardiovascular disease.[29] Misclassification may

Table 2. Risk stratification of all-cause mortality when combining FLI and non-invasive fibrosis scores.

Cox regression model	Follow-up period	Fibrosis score (intermediate *vs.* low risk)		Fibrosis score (high *vs.* low risk)		FLI (intermediate *vs.* low risk)		FLI (high *vs.* low risk)	
		HR (95% CI)	*p* value	HR (95% CI)	*p* value	HR (95% CI)	*p* value	HR (95% CI)	*p* value
FLI + FIB4	0-3 years	1.80 (1.61–2.00)	3.83E^{-27}	4.03 (3.23–5.04)	1.80E^{-34}	1.42 (1.35–1.50)	1.21E^{-37}	2.03 (1.93–2.13)	4.44E^{-187}
	4-6 years	1.68 (1.55–1.83)	1.95E^{-36}	3.78 (3.18–4.50)	8.32E^{-51}	–	–	–	–
	7-9 years	1.89 (1.76–2.03)	8.13E^{-71}	3.13 (2.64–3.71)	8.23E^{-40}	–	–	–	–
	>9 years	2.06 (1.90–2.23)	1.25E^{-66}	3.33 (2.70–4.11)	2.37E^{-29}	–	–	–	–
FLI + NFS	0-3 years	1.68 (1.51–1.88)	9.74E^{-21}	3.39 (2.63–4.37)	3.39E^{-21}	1.33 (1.26–1.41)	1.72E^{-23}	1.68 (1.60–1.77)	8.46E^{-90}
	4-6 years	1.71 (1.57–1.86)	2.52E^{-36}	2.89 (2.34–3.57)	5.40E^{-23}	–	–	–	–
	7-9 years	1.83 (1.70–1.97)	4.92E^{-60}	3.21 (2.69–3.84)	1.68E^{-37}	–	–	–	–
	>9 years	2.00 (1.84–2.18)	3.25E^{-58}	3.23 (2.55–4.08)	1.17E^{-22}	–	–	–	–
FLI + FIB4 + Cov	0-3 years	1.06 (0.94–1.18)	0.36	1.85 (1.46–2.33)	2.08E^{-07}	1.01 (0.95–1.07)	0.76	1.22 (1.16–1.28)	6.71E^{-14}
	4-6 years	0.94 (0.86–1.03)	0.16	1.61 (1.34–1.93)	2.37E^{-07}	–	–	–	–
	7-9 years	1.01 (0.94–1.09)	0.74	1.26 (1.06–1.50)	0.010	–	–	–	–
	>9 years	1.05 (0.96–1.15)	0.27	1.27 (1.03–1.58)	0.027	–	–	–	–
FLI + NFS + Cov	0-3 years	1.04 (0.93–1.16)	0.52	1.60 (1.23–2.07)	0.0004	1.00 (0.94–1.06)	0.97	1.20 (1.13–1.26)	8.3E^{-11}
	4-6 years	1.01 (0.92–1.10)	0.85	1.24 (1.00–1.53)	0.052	–	–	–	–
	7-9 years	1.03 (0.95–1.11)	0.47	1.27 (1.05–1.52)	0.012	–	–	–	–
	>9 years	1.05 (0.97–1.15)	0.24	1.15 (0.90–1.46)	0.263	–	–	–	–

Table shows time-dependent Cox regression results of FLI and one non-invasive fibrosis scores (FIB4 or NAFLD fibrosis score), adjusted and not adjusted for covariates, which include age, sex, Townsend index, type 2 diabetes mellitus, smoking status, and alcohol intake.
Cov, covariates; FIB4, fibrosis-4 index; FLI, fatty liver index; HR, hazard ratio; NFS, NAFLD fibrosis score.

hamper the prediction capacity of disease outcomes, but from our results, there were generally comparable comorbid event rates in NAFLD cases with a low-risk FLI and those with healthy livers and a high-risk FLI.

Bearing this in mind, our results show that the risk of ischaemic heart disease, stroke, diabetes, hypertension, hyperlipidaemia, hepatic malignancy, and all-cause mortality was stratified by FLI longitudinally over a median follow-up >10 years, after adjustment of known covariates associated with NAFLD development. Several studies have shown that FLI can predict the risk of cardiometabolic disease and overall mortality, which is consistent with our finding.[30–34] However, we have also shown that FLI predicts of the risk of hepatic and certain extrahepatic malignancies as well, which is a novel finding. A previous study has examined FLI stratification of cardiac events and stroke within the UKB, in which the authors used FLI deciles for modelling.[35] This may hamper ease of clinical interpretation given the index was originally designed as a tripartite classification system (low, intermediate and high risk for NAFLD). The presented results show a similar direction of the estimated effect, with our use of time-dependent models potentially providing more precise risk estimates in an updated analysis with a longer follow-up period. Separately, FLI has also been shown to risk stratify incident hypertension and dyslipidaemia in comparatively smaller Asian cohorts.[31,36] Our results therefore provide further information on FLI's utility for risk stratification, specifically in a UK cohort composed mainly of Caucasians.

We show that a high-risk FLI (>60) was predictive of a higher risk of hepatic malignancy when compared to an index score of <30. This conforms well with the current understanding that NAFLD-related metabolic risk factors increase the risk of disease progression. However, there was no overall risk prediction of incident extrahepatic malignancy, which is contrary to the association of NAFLD with the development of and mortality from extrahepatic malignancy.[37,38] The discrepancy is likely related to differences in the outcome investigated, whereby cancer-related mortality is more strongly predicted by metabolic comorbidities reflected by FLI but potentially less so with incidence of all types of cancer. Supporting this, in a recent study investigating incident cancer and cancer-related mortality, using raised alanine aminotransferase levels as a surrogate marker for NAFLD in three independent Scottish cohorts, it was shown that FLI predicted risk of mortality from cancer.[39] However, the differences in modelling and covariates with the present study could also influence these results. In other studies, FLI was shown to be associated with risk of colorectal, pancreatic and breast cancer development in a Korean population.[40–42] We also examined specific incidences of metabolism-related malignancies, namely colorectal, breast and upper GI cancers (oesophageal and gastric), and show that high-risk FLI was able to identify increased risk relative to low-risk scoring individuals, independent of common risk factors for NAFLD.

Lastly, we demonstrate that high-risk FLI was also associated with increased all-cause mortality independent of age, sex, diabetes, alcohol intake and socioeconomic deprivation, consistent with NAFLD and metabolic risk factors being predictive factors. To mimic the current two-step strategy of clinical NAFLD assessment which first stratifies risk of disease and then subsequently risk of advance disease, we explored whether FLI combined with non-invasive fibrosis scores can be used to assess mortality risk. Results show that non-invasive fibrosis scores and FLI independently stratified risk of all-cause mortality. Furthermore, in individuals with intermediate- and high-risk FLI, both fibrosis scores predicted all-cause mortality in their high-risk category in our adjusted model. This suggests FLI adds information to risk assessment of all-cause mortality and can potentially be used in tandem with fibrosis scores for this purpose.

Our study has certain limitations. The use of a listwise deletion cohort may have created bias, but our sensitivity analysis suggests our estimates were relatively precise when discounting for the largest missing variable (alcohol intake). Given the observational nature of the UKB, FLI's use in the general population and in individuals with NAFLD as a means of follow-up should be directly assessed in future intervention studies. Lastly, cost-effectiveness analysis will be required to build a case for implementation, which is beyond the scope of our study. With different components of FLI showing different directions of risk in certain disease outcomes assessed, further refinement of the index for specific outcomes may be attractive, but this must be balanced against the fact that diversification of risk stratification tools may increase complexity and thereby difficulty in clinical implementation.

Overall our results suggest that the FLI is an attractive potential option to identify individuals at risk of NAFLD and related comorbidities within the community, particularly during routine follow-up for other metabolic diseases in primary care. Whether the degree of misclassification we have identified is clinically acceptable warrants further debate, but it is probably better than the current standard of care which varies with geography and mostly amounts to doing nothing.[17] Clearly, as we have observed, the risk effect estimates observed from a single FLI may change over time, and thus repeated FLI measurements may provide better indication of comorbid disease and mortality risk, as shown by a previous study.[43]

In summary, the FLI identified prevalent and incident NAFLD and stratified the risk of incident cardiovascular and metabolic diseases, hepatic malignancy, and some extrahepatic cancers. FLI alone or in combination with NFS or FIB-4 independently enabled the risk assessment of all-cause mortality. FLI shows potential as a tool to stratify individuals for further assessment and to guide the development of prevention strategies for NAFLD and its related comorbidities in the population.

Abbreviations

ALT, alanine aminotransferase; AST, aspartate aminotransferase; AUROC, area under the ROC curve; FIB4, fibrosis-4 index; FLI, fatty liver index; GI, gastrointestinal; HR, hazard ratio; NAFLD, non-alcoholic fatty liver disease; NFS, NAFLD fibrosis score; PDFF, proton density fat fraction; ROC, receiver-operating characteristic; T2DM, type 2 diabetes mellitus; UKB, United Kingdom Biobank.

Financial support

BH is an MRC Clinical Training Fellow based at the University of Liverpool supported by the North West England Medical Research Council Fellowship Scheme in Clinical Pharmacology and Therapeutics, which is funded by the Medical Research Council (Award Ref. MR/N025989/1), Roche Pharma, Eli Lilly and Company Limited, UCB Pharma, Novartis, the University of Liverpool and the University of Manchester.

Conflicts of interest

M.P. has received partnership funding (to the University of Liverpool) for the following: MRC Clinical Pharmacology Training Scheme (co-funded by MRC and Roche, UCB, Eli Lilly and Novartis; this scheme was used to fund BH for this work); a PhD studentship jointly funded by EPSRC and Astra Zeneca; and grant funding from Vistagen Therapeutics. He has also unrestricted educational grant support for the UK Pharmacogenetics and Stratified Medicine Network from Bristol-Myers Squibb. He has developed an HLA genotyping panel with MC Diagnostics, but does not benefit financially from this. He is part of the IMI Consortium ARDAT (www.ardat.org). Other investigators have no conflicts of interest to declare.

Please refer to the accompanying ICMJE disclosure forms for further details.

Authors' contributions

Funding: MP, Manuscript concept: BH, AT and MP. Manuscript design and writing: BH. Statistical analysis: BH and AJ. Revision, editing and acceptance of final version: all authors.

Data availability statement

The data used for analysis is obtained from the UK Biobank and processes of obtaining this is stated in http://www.ukbiobank.ac.uk/using-the-resource/.

Supplementary data

Supplementary data to this article can be found online at https://doi.org/10.1016/j.jhepr.2023.100896.

References

Author names in bold designate shared co-first authorship

[1] Younossi ZM, Koenig AB, Abdelatif D, Fazel Y, Henry L, Wymer M. Global epidemiology of nonalcoholic fatty liver disease—meta-analytic assessment of prevalence, incidence, and outcomes. Hepatology 2016;64:73–84.

[2] Adejumo AC, Samuel GO, Adegbala OM, Adejumo KL, Ojelabi O, Akanbi O, et al. Prevalence, trends, outcomes, and disparities in hospitalizations for nonalcoholic fatty liver disease in the United States. Ann Gastroenterol 2019;32:504–513.

[3] Ekstedt M, Franzén LE, Mathiesen UL, Thorelius L, Holmqvist M, Bodemar G, et al. Long-term follow-up of patients with NAFLD and elevated liver enzymes. Hepatology 2006;44:865–873.

[4] Alexander M, Loomis AK, Van DerLei J, Duarte-Salles T, Prieto-Alhambra D, Ansell D, et al. Non-alcoholic fatty liver disease and risk of incident acute myocardial infarction and stroke: findings from matched cohort study of 18 million European adults. BMJ 2019;367:1–9.

[5] Alexander M, Loomis AK, Van DerLei J, Duarte-Salles T, Prieto-Alhambra D, Ansell D, et al. Risks and clinical predictors of cirrhosis and hepatocellular carcinoma diagnoses in adults with diagnosed NAFLD: real-world study of 18 million patients in four European cohorts. BMC Med 2019;17:1–9.

[6] Huang DQ, El-Serag HB, Loomba R. Global epidemiology of NAFLD-related HCC: trends, predictions, risk factors and prevention. Nat Rev Gastroenterol Hepatol 2020;18(4):223–238. 2020.

[7] Mofrad P, Contos MJ, Haque M, Sargeant C, Fisher RA, Luketic VA, et al. Clinical and histologic spectrum of nonalcoholic fatty liver disease associated with normal ALT values. Hepatology 2003;37:1286–1292.

[8] Browning JD, Szczepaniak LS, Dobbins R, Nuremberg P, Horton JD, Cohen JC, et al. Prevalence of hepatic steatosis in an urban population in the United States: impact of ethnicity. Hepatology 2004;40:1387–1395.

[9] **Hernaez R**, **Lazo M**, Bonekamp S, Kamel I, Brancati FL, Guallar E, et al. Diagnostic accuracy and reliability of ultrasonography for the detection of fatty liver: a meta-analysis. Hepatology 2011;54:1082.

[10] Alexander M, Loomis AK, Fairburn-Beech J, van derLei J, Duarte-Salles T, Prieto-Alhambra D, et al. Real-world data reveal a diagnostic gap in non-alcoholic fatty liver disease. BMC Med 2018;16(1):1–11. 2018.

[11] Yeoman AD. Novel approaches to detect significant liver disease in the general population. Clin Liver Dis 2021;18:99.

[12] **Chalmers J**, **Wilkes E**, Harris R, Kent L, Kinra S, Aithal G, et al. Original research: development and implementation of a commissioned pathway for the identification and stratification of liver disease in the community. Frontline Gastroenterol 2020;11:86.

[13] Dillon JF, Miller MH, Robinson EM, Hapca A, Rezaeihemami M, Weatherburn C, et al. Intelligent liver function testing (iLFT): a trial of automated diagnosis and staging of liver disease in primary care. J Hepatol 2019;71:699–706.

[14] Srivastava A, Gailer R, Tanwar S, Trembling P, Parkes J, Rodger A, et al. Prospective evaluation of a primary care referral pathway for patients with non-alcoholic fatty liver disease. J Hepatol 2019;71:371–378.

[15] Wong VWS, Adams LA, deLédinghen V, Wong GLH, Sookoian S. Noninvasive biomarkers in NAFLD and NASH — current progress and future promise. Nat Rev Gastroenterol Hepatol 2018;15:461–478.

[16] Bedogni G, Bellentani S, Miglioli L, Masutti F, Passalacqua M, Castiglione A, et al. The fatty liver index: a simple and accurate predictor of hepatic steatosis in the general population. BMC Gastroenterol 2006;6:1–7.

[17] Neilson LJ, Macdougall L, Lee PS, Hardy T, Beaton D, Chandrapalan S, et al. Implementation of a care bundle improves the management of patients with non-alcoholic fatty liver disease. Frontline Gastroenterol 2021;12:578–585.

[18] Sudlow C, Gallacher J, Allen N, Beral V, Burton P, Danesh J, et al. UK biobank: an open access resource for identifying the causes of a wide range of complex diseases of middle and old age. PLOS Med 2015;12:e1001779.

[19] Littlejohns, T. J., Holliday, J., Gibson, L. M., Garratt, S., Oesingmann, N., Alfaro-Almagro, F., et al. The UK Biobank imaging enhancement of 100, 000 participants: rationale, data collection, management and future directions. doi:10.1038/s41467-020-15948-9.

[20] Thompson A, Cook J, Choquet H, Jorgenson E, Yin J, Kinnunen T, et al. Functional validity, role, and implications of heavy alcohol consumption genetic loci. Sci Adv 2020;6.

[21] Angulo P, Hui JM, Marchesini G, Bugianesi E, George J, Farrell GC, et al. The NAFLD fibrosis score: a noninvasive system that identifies liver fibrosis in patients with NAFLD. Hepatology 2007;45:846–854.

[22] McPherson S, Stewart SF, Henderson E, Burt AD, Day CP. Simple non-invasive fibrosis scoring systems can reliably exclude advanced fibrosis in patients with non-alcoholic fatty liver disease. Gut 2010;59:1265–1269.

[23] Linge J, Borga M, West J, Tuthill T, Miller MR, Dumitriu A, et al. Body composition profiling in the UK biobank imaging study. Obesity 2018;26:1785–1795.

[24] Wilman HR, Kelly M, Garratt S, Matthews PM, Milanesi M, Herlihy A, et al. Characterisation of liver fat in the UK Biobank cohort. PLoS One 2017;12:1–14.

[25] Cuthbertson DJ, Weickert MO, Lythgoe D, Sprung VS, Dobson R, Shoajee-Moradie F, et al. External validation of the fatty liver index and lipid accumulation product indices, using 1H-magnetic resonance spectroscopy, to identify hepatic steatosis in healthy controls and obese, insulin-resistant individuals. Eur J Endocrinol 2014;171:561–569.

[26] Lee JH, Kim D, Kim HJ, Lee CH, Yang JI, Kim W, et al. Hepatic steatosis index: a simple screening tool reflecting nonalcoholic fatty liver disease. Dig Liver Dis 2010;42:503–508.

[27] Castellana M, Donghia R, Guerra V, Procino F, Lampignano L, Castellana F, et al. Performance of fatty liver index in identifying non-alcoholic fatty liver disease in population studies. A meta-analysis. J Clin Med 2021;10:10.

[28] Cuthbertson DJ, Koskinen J, Brown E, Magnussen CG, Hutri-Kähönen N, Sabin M, et al. Fatty liver index predicts incident risk of prediabetes, type 2 diabetes and non-alcoholic fatty liver disease (NAFLD). Ann Med 2021;53:1256–1264. https://doi.org/10.1080/07853890.2021.1956685.

[29] Hippisley-Cox J, Coupland C, Brindle P. Development and validation of QRISK3 risk prediction algorithms to estimate future risk of cardiovascular disease: prospective cohort study. BMJ 2017;357.

[30] Franch-Nadal J, Caballeria L, Mata-Cases M, Mauricio D, Giraldez-García C, Mancera J, et al. Fatty liver index is a predictor of incident diabetes in patients with prediabetes: the PREDAPS study. PLoS One 2018;13:e0198327.

[31] Huh JH, Ahn SV, Koh SB, Choi E, Kim JY, Sung K-C, et al. A prospective study of fatty liver index and incident hypertension: the KoGES-ARIRANG study. PLoS One 2015;10:e0143560.

[32] Khang AR, Lee HW, Yi D, Kang YH, Son SM. The fatty liver index, a simple and useful predictor of metabolic syndrome: analysis of the Korea National Health and Nutrition Examination Survey 2010–2011. Diabetes Metab Syndr Obes Targets Ther 2019;12:181.

[33] Kim JYJH, Moon JS, Byun SJ, Lee JH, Kang DR, Sung KC, et al. Fatty liver index and development of cardiovascular disease in Koreans without pre-existing myocardial infarction and ischemic stroke: a large population-based study. Cardiovasc Diabetol 2020;19:1–9.

[34] Yadav D, Choi E, Ahn SV, Koh SB, Sung KC, Kim JY, et al. Fatty liver index as a simple predictor of incident diabetes from the KoGES-ARIRANG study. Med (United States) 2016:95.

[35] Zou B, Yeo YH, Cheung R, Ingelsson E, Nguyen MH. Fatty liver index and development of cardiovascular disease: findings from the UK biobank. Dig Dis Sci 2021;66(6):2092–2100. 2021.

[36] **Higashiura Y**, **Furuhashi M**, **Tanaka M**, Takahashi S, Mori K, Miyamori D, et al. Elevated fatty liver index is independently associated with new

onset of hypertension during a 10-year period in both male and female subjects. J Am Hear Assoc Cardiovasc Cerebrovasc Dis 2021;10:21430.
[37] Musso G, Gambino R, Cassader M, Pagano G. Meta-analysis: natural history of non-alcoholic fatty liver disease (NAFLD) and diagnostic accuracy of non-invasive tests for liver disease severity. Ann Med 2011;43: 617–649.
[38] Liu Y, Zhong GC, Tan HY, Hao FB, Hu JJ. Nonalcoholic fatty liver disease and mortality from all causes, cardiovascular disease, and cancer: a meta-analysis. Sci Rep 2019;9:11124.
[39] Taylor A, Siddiqui MK, Ambery P, Armisen J, Challis BG, Haefliger C, et al. Metabolic dysfunction-related liver disease as a risk factor for cancer. BMJ Open Gastroenterol 2022;9:e000817.
[40] Park JH, Hong JY, Han K, Kang W, Park JK. Increased risk of pancreatic cancer in individuals with non-alcoholic fatty liver disease. Sci Rep 2022;12(1):1–8. 2022.
[41] Park JH, Choi IS, Han KDo, Park H, Kim KH, Kim JS. Association between fatty liver index and risk of breast cancer: a nationwide population-based study. Clin Breast Cancer 2020;20:e450–e457.
[42] Choi YJ, Lee DH, Han KDo. Association between high fatty liver index and development of colorectal cancer: a nationwide cohort study with 21,592, 374 Korean. Korean J Intern Med 2020;35:1354.
[43] Lee C-H, Han K-D, Kim DH, Kwak M-S. The repeatedly elevated fatty liver index is associated with increased mortality: a population-based cohort study. Front Endocrinol (Lausanne) 2021;0:73.

Journal of Hepatology, Volume *5*

Supplemental information

Role of fatty liver index in risk-stratifying comorbid disease outcomes in non-alcoholic fatty liver disease

Brian Ho, Andrew Thompson, Andrea L Jorgensen, and Munir Pirmohamed

Role of fatty liver index in risk-stratifying comorbid disease outcomes in non-alcoholic fatty liver disease

Brian Ho, Andrew Thompson, Andrea L Jorgensen, Munir Pirmohamed

Table of contents

Supplementary materials..2

Supplementary figures...6

Supplementary tables...14

Supplementary methods

Disease Outcomes/Events Definitions

All disease covariates and outcomes were identified using ICD-9/ICD-10, primary care and death registry information. UK cancer registry data was used for identification of additional cases of hepatic and extrahepatic malignancies. Similarly, for diabetes, HBA1c measurements performed during UKB assessment attendances, using the WHO threshold of >48 mmol/mol were used, while GP prescription data of antidiabetic drugs were used to find additional cases of T2DM. Lastly GP prescription of statin or fibrates were also used to identify individuals with dyslipidaemia. Specific metabolic syndrome/metabolism related malignancy outcomes were also extracted using diagnostic codes, which are colon, breast and upper GI (oesophageal and stomach) cancers. Incidence rate of diseases were calculated for each FLI category.

Receiver Operating Characteristic Analysis

Receiver operating characteristics were examined and area under the curve was used to assess FLI, hepatic steotosis index, and lipid accumulation product performance in identifying combined prevalent and incident NAFLD, i.e a cross-sectional analysis. Case definitions were defined by MRI-PDFF value derived by two separate companies for the UK Biobank (Perspectum Diagnostics and AMRA). Additional case finding was performed using ICD9/10 clinical codes and primary care record data, which both shared in their definition. These non-invasive scores was therefore tested against two case control definitions of NAFLD in a small subset of UK Biobank participants which have available MRI and healthcare records data. Optimum cut off for was also derived using greatest distance from line of no-effect, i.e 0.50 AUC line. AUROC was calculated and 95% CIs of ROC sensitivities were obtained through stratified

bootstrapping data 2000 times at intervals of 5% for specificities and the distribution smoothened with the loess method.

Time to Event Analysis

Cox proportional hazards model was used for time to event analyses. Study start date was defined at recruitment to UK Biobank when FLI score was derived. Listwise deletion dataset was used in Cox regression modelling and individuals who have experienced the event of interest prior to study start date were excluded from the respective time to event analysis. Univariate regression was first performed for each time to event outcome for exposures of interest. A p-value of <0.1 was used as threshold for inclusion for downstream multivariate analysis. To assess linearity of Cox regression model, penalised splines were applied to continuous covariates to assess for significant non-linear component in univariate cox regression. Exposure covariates with significant non-linear component ($p < 0.05$) were subsequently regressed by applying a natural cubic spline for better model fitting. T2DM, as an exposure of interest, was fitted as a time dependent covariate for all models, except in the model where T2DM is the event of interest. Subsequently, two models were fitted to assess risk stratification by FLI: the first model is termed the "Basic Model", which included covariates age and sex, and the second model included additional covariates brought forward from univariate regression. Finally, proportional hazards assumption was tested in these models using Schoenfeld residuals. If breaches of assumptions were found, defined again by significance level of $p<0.05$, time dependent coefficients were created using a step function, which divided follow-up from FLI into 0-3, 4-6, 7-9 and >9 years. This division in follow-up period as a step function from UK Biobank recruitment was chosen from the variation in median >10 years observed in our UK Biobank participants in all our time to event analyses. Models whereby FLI was treated

as categorical variable was additionally assessed with likelihood ratio test between models with and without FLI as a covariate. This allowed testing whether FLI provided additional predictive risk stratification for event of interest as a single covariate. Both dummy variable p-values within the model and model comparison tests p-values are reported. P-values <0.05 were considered significant after Bonferonni correction for multiple testing (this is 26 models/tests for FLI, $p < 0.0019$)

Additional and Sensitivity Analyses

An additional analysis was performed to look at incidence rates of misclassified individuals, with high-risk FLI but normal livers on MRI and low-risk FLI with evidence/diagnosis of NAFLD. Two sensitivity analyses were performed. Firstly, examining time to event analysis using components used to calculate FLI (BMI, waist circumference, serum GGT and total triglyceride levels) in multivariate Cox regression. This is to allow estimate of significance and risk estimate direction for each of these components toward the incident disease outcome. Secondly, listwise deletion cohort with/without accounting for missing data from alcohol intake for each time to event analyses was examined. Alcohol has the highest overall proportion of missing data of 31% and allows best chance to see examine whether exclusion of missing broadly altered the model risk estimates.

Incident All-cause Mortality with FLI and Fibrosis Scores

For incident all-cause mortality, a similar approach was adopted for Cox regression analysis investigating risk stratification by FLI and non-invasive fibrosis scores (NAFLD Fibrosis Score and FIB4). Univariate modelling and assumptions testing was performed similarly as detailed above for FLI on incident disease outcomes. Separate multivariate modelling for fibrosis scores was further performed after

stratifying individuals by FLI risk to examine NFS and FIB4's performance in these subgroups. Further multivariate modelling included FLI, and a single fibrosis score (NFS or FIB4) was used or with all exposure covariates brought forward by univariate analysis. This allowed examining differences in risk effect estimates given by fibrosis scores and the effect exposure covariates have. P-values <0.05 were considered significant after Bonferonni correction for multiple testing.

Supplementary figures

Fig. S1 – NAFLD Case Definition Flowchart

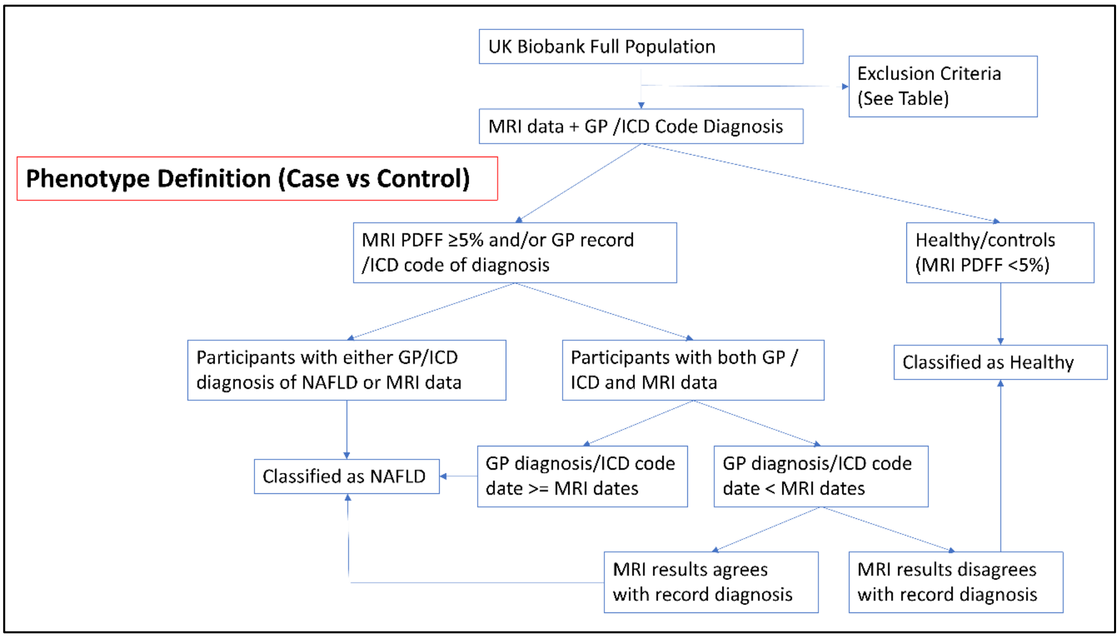

Flowchart shows the derivation of case control definition of NAFLD used based on inpatient hospital ICD diagnostic codes, primary care record data, and available MRI proton density fat fraction generated by companies Perspectum Diagnostic and AMRA. The case-control provides an assessment of ground truth for analysing FLI's capability in cross-sectional disease identification (i.e identifying both incident and prevalent NAFLD).

Fig. S2 - Diagnostic Performance at Low and High Cut-offs of FLI

Diagram here shows the equation used to derive Fatty Liver Index score as published by Bedogni et al, 2016. Cut-offs chosen here are recommended cut-offs for stratifying risk of NAFLD at point of care. Frequency tables and cross-sectional diagnostic performance (sensitivity, specificity, positive predictive value (PPV) and negative predictive value (NPV)) are shown for these cut-offs is being used as a binary classifier for NAFLD. Our NAFLD ground truth is defined by diagnostic codes and MRI measurements (See Figures S1). The 1st definition denotes case control definition derived from MRI proton density fat fraction from Perspectum Diagnostics and 2nd definition originates from AMRA; both cohorts haring clinically/primary care coded cases.

Fatty Liver Index = $e^y / (1 + e^y) \times 100$

y = 0.953 x ln(triglyceride levels (mg/dL) + 0.139 x BMI (kg/m2) + 0.718 x ln(GGT (U/L)) + 0.053 x (waist circumference - 15.745)

1st Case Control Definition

Low cut off (≥30)

Case/Control<br>----------<br>Index Classification	Healthy	NAFLD
Healthy	1182	379
NAFLD	1029	5066

Sensitivity – 93.04%
Specificity – 53.46%
PPV – 75.03%
NPV – 83.63%

High cut-off (≥60)

Case/Control<br>----------<br>Index Classification	Healthy	NAFLD
Healthy	1781	1346
NAFLD	430	4099

Sensitivity – 75.28%
Specificity – 80.55%
PPV – 56.04%
NPV – 90.82%

2nd Case Control Definition

Low cut off (≥30)

Case/Control<br>----------<br>Index Classification	Healthy	NAFLD
Healthy	2489	459
NAFLD	2174	5655

Sensitivity – 92.49%
Specificity – 53.38%
PPV – 84.15%
NPV – 72.65%

High cut-off (≥60)

Case/Control<br>----------<br>Index Classification	Healthy	NAFLD
Healthy	3758	1627
NAFLD	905	4487

Sensitivity – 73.39%
Specificity – 80.59%
PPV – 69.34%
NPV – 83.51%

Fig. S3 – Listwise Deletion Flow Chart for Outcomes of Interest

Flowchart showing derivation of the listwise deletion dataset from UK Biobank for each incident disease outcome. The final numbers were subsequently used in each individual time to event analysis.

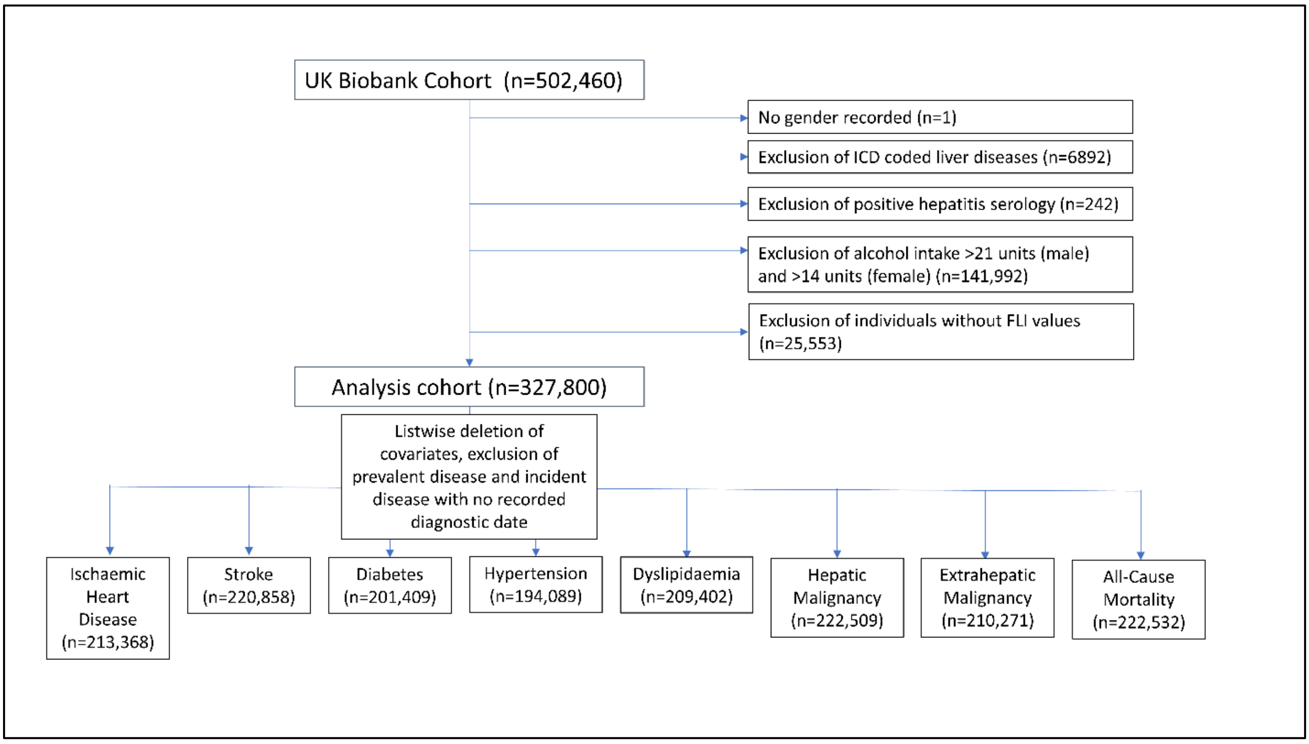

Fig. S4 – Incident All-cause Mortality By Fibrosis Scores

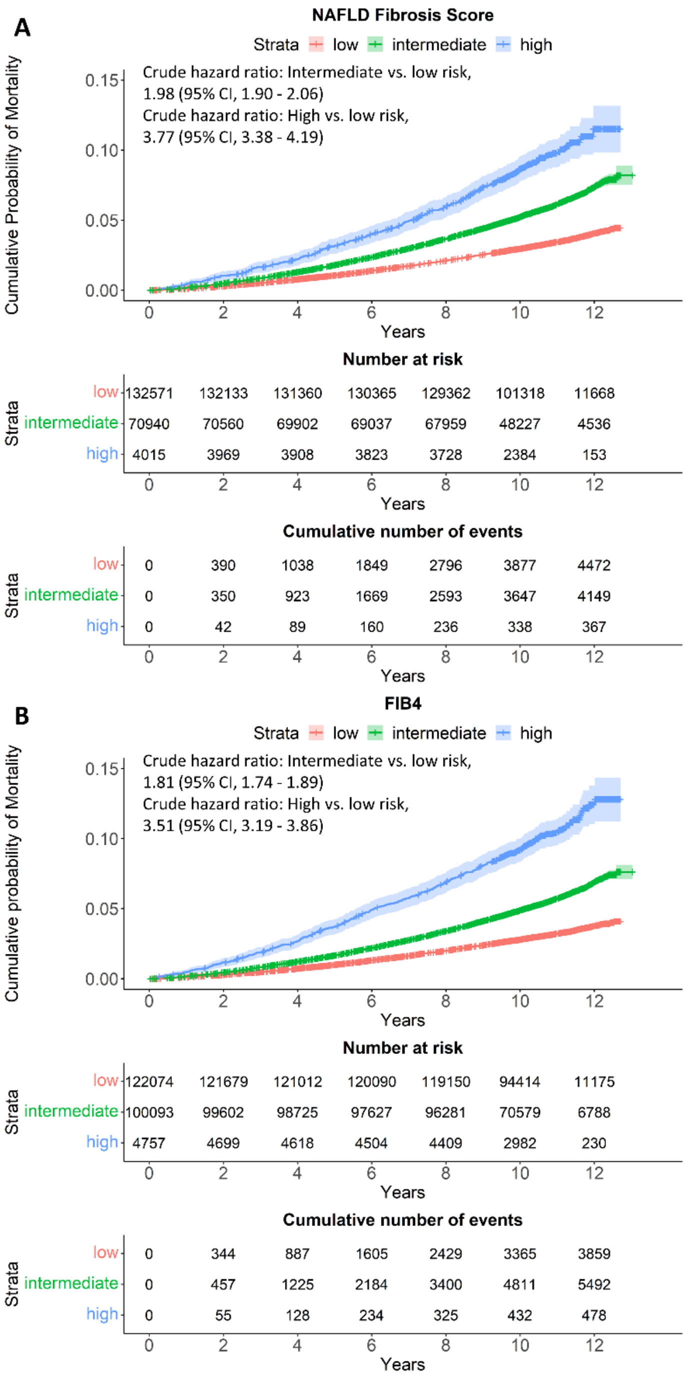

Kaplan-Meier Curves showing incident all-cause mortality comparing individuals with low, intermediate and high-risk (A) NAFLD fibrosis score and (B) FIB4. Crude hazard ratios and 95% CI (shaded area) are derived from univariate Cox regression modelling to give broad interpretation of risk. + is used to mark individuals who are censored.

Fig. S5 – Incident All-cause Mortality By Fibrosis Scores Stratified By FLI Risk

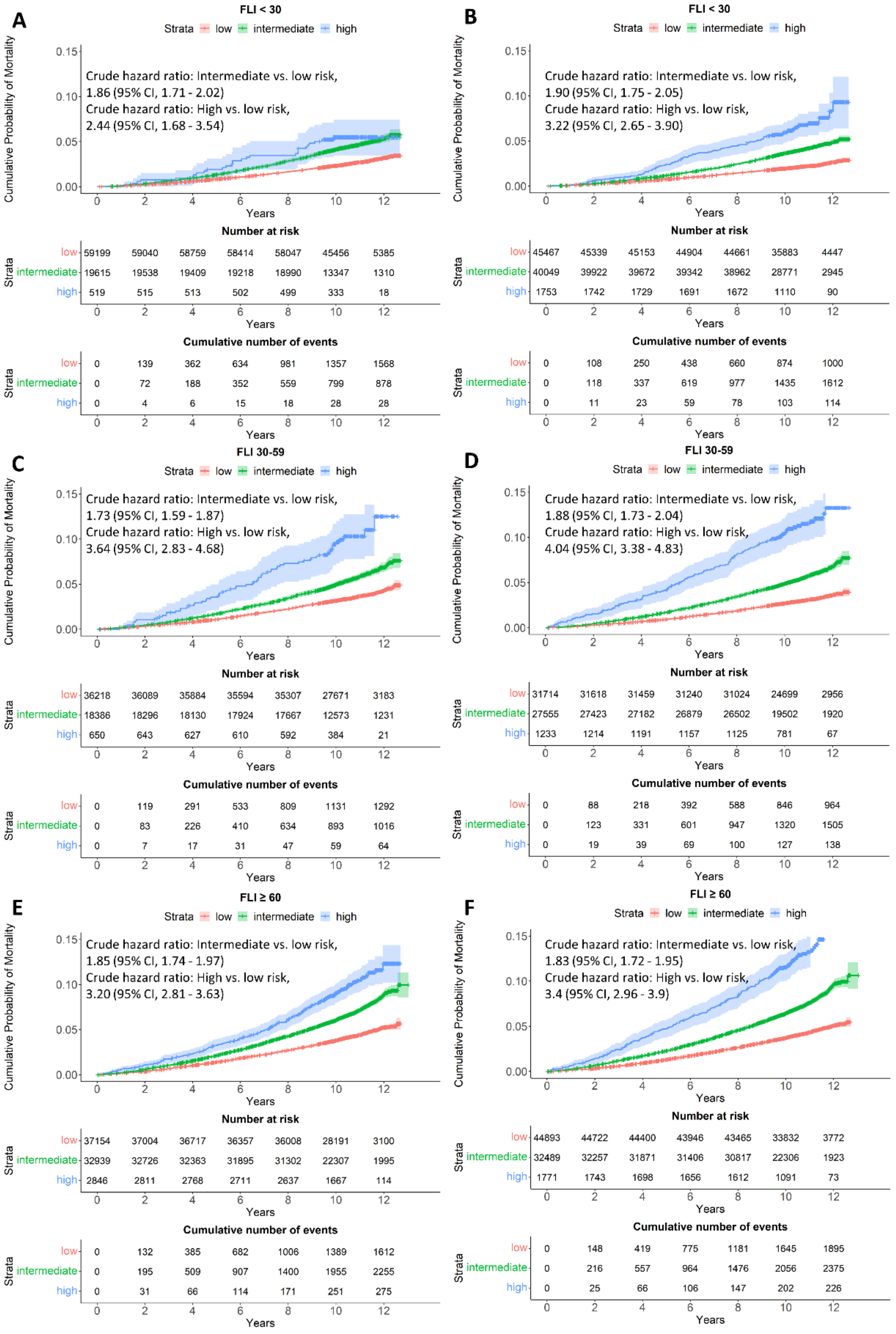

The Kaplan-Meier Curves are stratified by FLI risk categories: low – first row, intermediate – second row, high – third row. Cumulative incidence by risk categories of NAFLD fibrosis score (right column) and FIB4 (left column) are shown. Crude hazard ratios and 95% Cis (shaded area) are obtained from univariate Cox regression analysis. + signs mark censored individuals.

Fig. S6 - Unified Model FIB4 or NAFLD Fibrosis Score with FLI For All-Cause Mortality

Model	Term	Follow-up Period	HR (95% CI)	p-value
FLI + FIB4	Fibrosis score intermediate vs. low risk	0-3 years	1.8 (1.61-2)	2.28e-258
		4-6 years	1.68 (1.55-1.83)	
		7-9 years	1.89 (1.76-2.03)	
		>9 years	2.06 (1.9-2.23)	
	Fibrosis score high vs. low risk	0-3 years	4.03 (3.23-5.04)	
		4-6 years	3.78 (3.18-4.5)	
		7-9 years	3.13 (2.64-3.71)	
		>9 years	3.33 (2.7-4.11)	
FLI + FIB4 + Covariates	Fibrosis score intermediate vs. low risk	0-3 years	1.06 (0.94-1.18)	1.23e-11
		4-6 years	0.94 (0.86-1.03)	
		7-9 years	1.01 (0.94-1.09)	
		>9 years	1.05 (0.96-1.15)	
	Fibrosis score high vs. low risk	0-3 years	1.85 (1.46-2.33)	
		4-6 years	1.61 (1.34-1.93)	
		7-9 years	1.26 (1.06-1.5)	
		>9 years	1.27 (1.03-1.58)	
FLI + NFS	Fibrosis score intermediate vs. low risk	0-3 years	1.68 (1.51-1.88)	1.93e-198
		4-6 years	1.71 (1.57-1.86)	
		7-9 years	1.83 (1.7-1.97)	
		>9 years	2 (1.84-2.18)	
	Fibrosis score high vs. low risk	0-3 years	3.39 (2.63-4.37)	
		4-6 years	2.89 (2.34-3.57)	
		7-9 years	3.21 (2.69-3.84)	
		>9 years	3.23 (2.55-4.08)	
FLI + NFS + Covariates	Fibrosis score intermediate vs. low risk	0-3 years	1.04 (0.93-1.16)	0.00487
		4-6 years	1.01 (0.92-1.1)	
		7-9 years	1.03 (0.95-1.11)	
		>9 years	1.05 (0.96-1.15)	
	Fibrosis score high vs. low risk	0-3 years	1.6 (1.23-2.07)	
		4-6 years	1.24 (1-1.53)	
		7-9 years	1.27 (1.05-1.52)	
		>9 years	1.15 (0.9-1.46)	

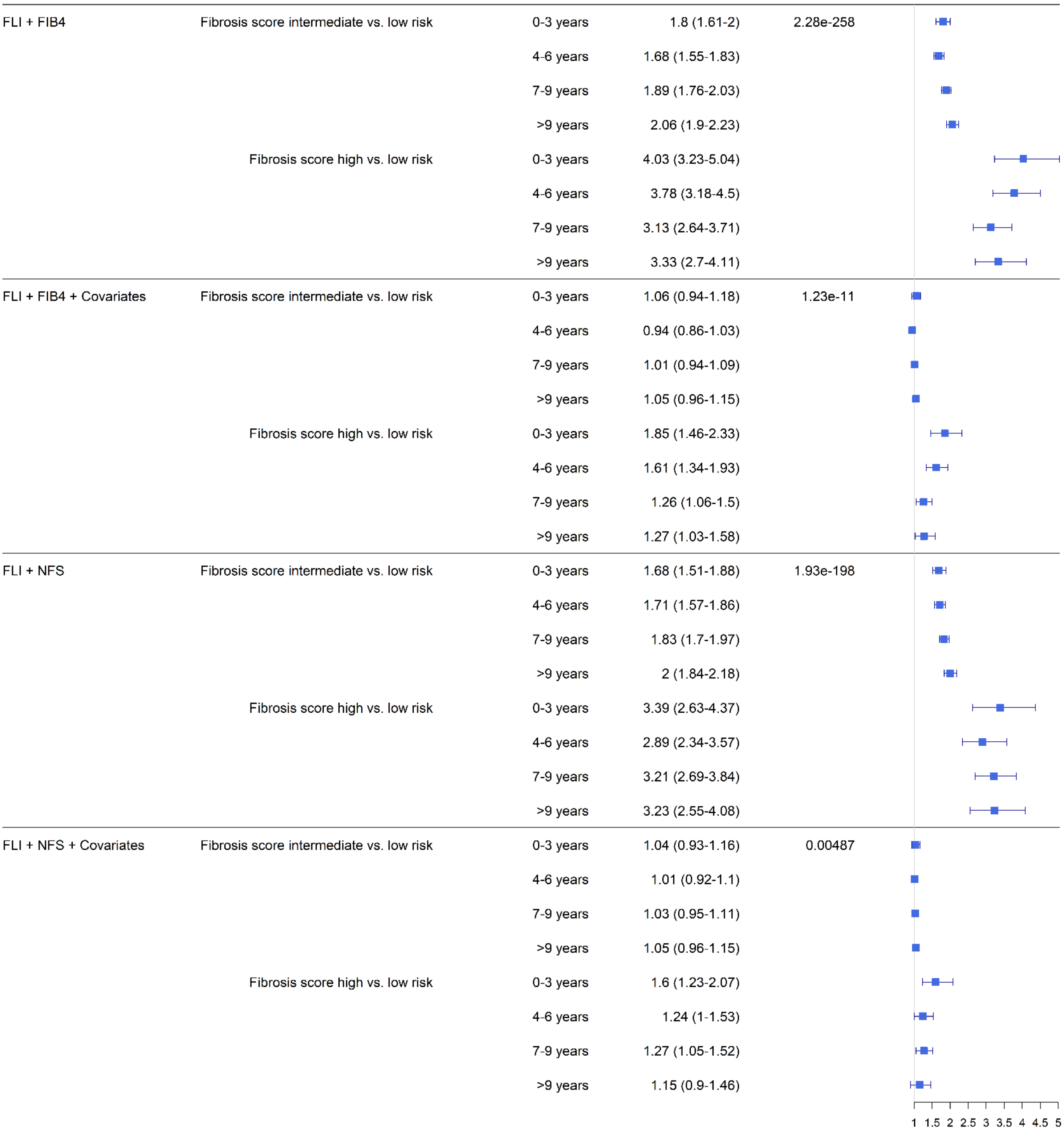

Forest plot showing hazard ratios of intermediate and high-risk non-invasive fibrosis score from Cox regression models. Regressors include FLI alone and FLI with other covariate exposures (age, sex, Townsend Index, smoking status, alcohol intake and T2DM diagnosis). Both FLI and fibrosis scores were treated as dummy variables here. Proportional hazard assumption was violated in all models and thus time dependent coefficients were utilised, dividing follow-up period into intervals of 0-3, 4-6, 7-9 and >9 years from time of risk score calculation. P-values are derived from likelihood ratio test between models with and without (null model) fibrosis score as a regressor.

Supplementary tables

Table S1 – ICD 9 Diagnostic Codes and Definitions For Exclusion

**VIRAL HEPATITIS**	
0700	Viral hepatitis a with hepatic coma
0701	Viral hepatitis a without mention of hepatic coma
0702	Viral hepatitis b with hepatic coma
0703	Viral hepatitis b without mention of hepatic coma
0704	Other specified viral hepatitis with hepatic coma
0705	Other specified viral hepatitis without mention of hepatic coma
0706	Unspecified viral hepatitis with hepatic coma
0709	Unspecified viral hepatitis without mention of hepatic coma
5732	Hepatitis in other infectious diseases classified elsewhere
5731	Hepatitis in viral diseases classified elsewhere
V026	Carrier or suspected carrier of viral hepatitis
77440	Perinatal jaundice due to hepatocellular damage (from hepatitis a)
77441	Perinatal jaundice due to hepatocellular damage (from hepatitis b)
77448	Perinatal jaundice due to other specified hepatitis
**ALCOHOL LIVER DISEASE**	
5710	Alcoholic fatty liver
5711	Acute alcoholic hepatitis
5712	Alcoholic cirrhosis of liver
5713	Alcoholic liver damage, unspecified
**AUTOIMMUNE LIVER DISEASE**	
5714	Chronic hepatitis
**WILSON's DISEASE**	
2751	Disorders of copper metabolism
**BILIARY CIRRHOSIS**	
5716	Biliary Cirrhosis
**IRON METABOLISM RELATED LIVER DISEASE**	
27509	Disorders of iron metabolism (other and unspecified)
27501	Haemosiderosis
27502	Haemochromatosis

**ALPHA-1 ANTITRYPSIN DEFICIENCY**	
27761	Other deficiencies of circulating enzymes (alpha-1-antitrypsin hepatitis)
27762	Other alpha-1-antitrypsin deficiency
**HEPATOVENOUS DISEASE**	
4530	Budd-chiari syndrome
**PRIMARY SCLEROSING CHOLANGITIS**	
5761	Cholangitis
**OTHER**	
27103	Glycogenosis (associated with hepatic cirrhosis)
57152	Cirrhosis of liver without mention of alcohol (childhood function) - Indian childhood

Table S2 – ICD 10 Diagnostic Codes and Definitions For Exclusion

**VIRAL HEPATITIS**	
B160	Acute hepatitis B with delta-agent (coinfection) with hepatic coma
B161	Acute hepatitis B with delta-agent (coinfection) without hepatic coma
B162	Acute hepatitis B without delta-agent with hepatic coma
B169	Acute hepatitis B without delta-agent and without hepatic coma
B171	Acute hepatitis C
B172	Acute hepatitis E
B178	Other specified acute viral hepatitis
B179	Acute viral hepatitis, unspecified
B180	Chronic viral hepatitis B with delta-agent
B181	Chronic viral hepatitis B without delta-agent
B182	Chronic viral hepatitis C
B189	Chronic viral hepatitis, unspecified
B190	Unspecified viral hepatitis with coma
B199	Unspecified viral hepatitis without coma
B251	Cytomegaloviral hepatitis
B942	Sequelae of viral hepatitis
O984	Viral hepatitis complicating pregnancy, childbirth and the puerperium
P353	Congenital viral hepatitis
Z225	Carrier of viral hepatitis
**ALCOHOL LIVER DISEASE**	
K700	Alcoholic fatty liver
K701	Alcoholic hepatitis
K702	Alcoholic fibrosis and sclerosis of liver
K703	Alcoholic cirrhosis of liver
K704	Alcoholic hepatic failure
K709	Alcoholic liver disease, unspecified
**TOXIC LIVER DISEASE**	
K710	Toxic liver disease with cholestasis
K711	Toxic liver disease with hepatic necrosis
K712	Toxic liver disease with acute hepatitis

K713	Toxic liver disease with chronic persistent hepatitis
K715	Toxic liver disease with chronic active hepatitis
K716	Toxic liver disease with hepatitis, not elsewhere classified
K717	Toxic liver disease with fibrosis and cirrhosis of liver
K718	Toxic liver disease with other disorders of liver
K719	Toxic liver disease, unspecified
K753	Granulomatous hepatitis, not elsewhere classified
**AUTOIMMUNE DISEASE**	
K754	Autoimmune hepatitis
**WILSON’S DISEASE**	
E830	Disorders of copper metabolism
**PRIMARY BILIARY CIRRHOSIS**	
K743	Primary biliary cirrhosis
K744	Secondary biliary cirrhosis
K745	Biliary cirrhosis, unspecified
**IRON METABOLISM RELATED LIVER DISEASE**	
E831	Disorders of iron metabolism

S3 – ICD and Primary Care (Read2 & Read3) Codes for NAFLD Classification

The table denotes diagnostic codes and respective clinical definitions used for case finding of NAFLD individuals in the UK Biobank (ICD HESIN and GP primary care dataset). These are used in conjunction with MRI data.

ICD Code	Definition
K760 (ICD10)	Fatty (change of) liver, not elsewhere classified
5718 (ICD9)	Other chronic non-alcoholic liver disease
**Read2**	**Definition**
J61y	Other non-alcoholic chronic liver disease
J61y1	Non-alcoholic fatty liver
J61y7	Steatosis of liver
J61y9	Fatty Change of Liver
**Read3**	**Definition**
J61y.	Other non-alcoholic chronic liver disease
J61y1	Non-alcoholic fatty liver
J61y7	Steatosis of liver
X307v	Fatty change of liver

S4 - Incidence Rates of Disease Outcomes by FLI Category

Table shown looks at number of individuals at risk, person-years of and event frequency for each FLI risk groups over the follow-up period: <30 (low risk), 30-59 (intermediate risk) and ≥60 (high risk). Incident disease per 100-person years subsequently calculated and shown here.

Disease Outcome	FLI Risk group	person-years	n	Event Frequency	Event per 100-person years
Ischaemic Heart Disease	Low	903312	86112	2827	0.31
	Intermediate	590376	57349	3645	0.62
	High	703059	69907	6513	0.93
Stroke	Low	923394	87310	971	0.11
	Intermediate	621927	59311	1060	0.17
	High	770244	74237	1527	0.20
Hypertension	Low	829440	82193	8844	1.07
	Intermediate	501032	52403	10153	2.03
	High	530773	59493	17263	3.25
Dyslipidaemia	Low	881732	85227	4905	0.56
	Intermediate	563581	56262	6061	1.08
	High	655883	67913	10817	1.65
Type 2 Diabetes Mellitus	Low	860877	82016	2124	0.25
	Intermediate	568649	54924	2487	0.44
	High	644163	64469	6660	1.03
Hepatic Malignancy	Low	932102	87716	67	0.01
	Intermediate	631477	59762	57	0.01
	High	785207	75031	133	0.02
Extrahepatic Malignancy	Low	769905	79256	9740	1.22
	Intermediate	534473	53902	7875	1.47
	High	670190	68153	10423	1.56

S5- Unadjusted Incidence Rates of Comorbidites in Individuals Misclassified by FLI with and without NAFLD.

	Incidence (per 100-person years)			
	Perspectum Algorithm Definition		AMRA Definition	
Outcome	Low-Risk FLI with NAFLD	High Risk FLI without NAFLD	Low-Risk FLI with NAFLD	High Risk FLI without NAFLD
Ischaemic Heart Disease	0.27	0.26	0.27	0.3
Ischaemic Stroke	0.025	0.087	0.021	0.052
Type 2 Diabetes Mellitus	0.27	0.27	0.27	0.18
Hypertension	0.92	1.28	0.88	1.08
Dyslipidaemia	0.58	0.88	0.51	0.73
Hepatic Malignancy	0	0.021	0	0.01
Extrahepatic Malignancy	0.94	0.69	0.77	0.61
All-cause Mortality	0.51	0.085	0.44	0.11

Table showing incidence rate of comorbid outcomes in misclassified individuals whereby they have low-risk FLI but found to have hepatic steatosis and high-risk FLI, but normal liver based on MRI-PDFF and ICD9/10 classification. The definition of NAFLD is based on MRI-PDFF values derived by Perspectum Diagnostics (our first definition) and AMRA (our second definition), whereas the diagnostic code is obtained from a common source: diagnostic codes from hospital inpatient records.

S6 - Univariate Cox Regression: FLI and Covariates for Disease Outcomes

Table showing univariate cox regression modelling outputs (hazards ratios and 95% CI) with associated with p-values disease outcomes with all exposures of interest. These are age, alcohol intake and Townsend index were treated as continuous variables, whereas sex, diabetes smoking status, FLI were treated as categorical variables using dummy variables. P <0.1 was used as the threshold for variable selection for downstream multivariate regression modelling.

Outcome	Covariate	HR (95% CI)	p-value
Ischaemic Heart Disease	Age (years)	1.08 (1.08 - 1.09)	<1 E-100
	Male (reference Female)	1.79 (1.74 - 1.83)	<1 E-100
	Alcohol Intake (units/week)	1.02 (1.01- 1.02)	3.26E-23
	Previous smoker (reference non-smoker)	1.48 (1.42 - 1.54)	3.68E-77
	Current smoker (reference non-smoker)	0.88 (0.85 - 0.91)	1.45E-14
	Diabetes (reference healthy)	1.62 (1.55 - 1.69)	1.86E-101
	Townsend Index	1.03 (1.02 - 1.03)	6.60E-17
	FLI intermediate vs. low risk	1.98 (1.88 - 2.08)	1.34E-162
	FLI high vs. low risk	2.97 (2.84 - 3.1)	<1 E-100
Stroke	Age (years)	1.10 (1.10 - 1.10)	7.75E-288
	Male (reference Female)	1.37 (1.31 - 1.44)	1.08E-40
	Alcohol Intake (units/week)	1.01 (1.01 - 1.02)	2.55E-04
	Previous smoker (reference non-smoker)	1.32 (1.22 - 1.43)	2.34E-11
	Current smoker (reference non-smoker)	0.91 (0.86 - 0.97)	5.83E-03
	Diabetes (reference healthy)	1.54 (1.42 - 1.68)	2.06E-24
	Townsend Index	1.02 (1.01 - 1.03)	5.90E-05
	FLI intermediate vs. low risk	1.62 (1.49 - 1.77)	1.29E-27
	FLI high vs. low risk	1.89 (1.74 - 2.05)	3.56E-54
Hypertension	Age (years)	1.08 (1.08 - 1.08)	<1 E-100
	Male (reference Female)	1.26 (1.24 - 1.27)	1.35E-206

	Alcohol Intake (units/week)	1.00 (1.00 - 1.01)	4.25E-03
	Previous smoker (reference non-smoker)	1.19 (1.16 - 1.23)	9.69E-38
	Current smoker (reference non-smoker)	0.85 (0.83 - 0.87)	7.75E-53
	Diabetes (reference healthy)	1.54 (1.50 - 1.59)	2.30E-188
	Townsend Index	1.03 (1.02 - 1.03)	2.11E-44
	FLI intermediate vs. low risk	1.9 (1.85 - 1.96)	<1 E-100
	FLI high vs. low risk	3.05 (2.98 - 3.13)	<1 E-100
Dyslipidaemia	Age (years)	1.08 (1.08 - 1.08)	<1 E-100
	Male (reference Female)	1.34 (1.32 - 1.37)	2.10E-208
	Alcohol Intake (units/week)	1.00 (1.00 - 1.01)	4.17E-02
	Previous smoker (reference non-smoker)	1.41 (1.37 - 1.46)	1.57E-95
	Current smoker (reference non-smoker)	0.88 (0.86 - 0.91)	2.04E-21
	Diabetes (reference healthy)	1.76 (1.70 - 1.82)	8.89E-238
	Townsend Index	1.05 (1.04 - 1.05)	3.66E-86
	FLI intermediate vs. low risk	1.93 (1.86 - 2.01)	3.36E-258
	FLI high vs. low risk	2.96 (2.87 - 3.07)	0.00E+00
Diabetes	Age (years)	1.05 (1.04 - 1.05)	3.00E-267
	Male (reference Female)	1.36 (1.32 - 1.40)	6.55E-117
	Alcohol Intake (units/week)	1.00 (0.99 - 1.00)	5.04E-03
	Previous smoker (reference non-smoker)	1.33 (1.27 - 1.39)	2.79E-34
	Current smoker (reference non-smoker)	0.92 (0.88 - 0.95)	1.39E-06
	Townsend Index	1.04 (1.04 - 1.05)	4.83E-41
	FLI intermediate vs. low risk	1.77 (1.67 - 1.88)	1.33E-83
	FLI high vs. low risk	4.19 (3.99 - 4.4)	<1 E-100
Hepatic Malignancy	Age (years)	1.09 (1.07 - 1.11)	1.43E-18
	Male (reference Female)	1.22 (1.03 - 1.45)	2.42E-02
	Alcohol Intake (units/week)	0.99 (0.97 - 1.02)	5.53E-01
	Previous smoker (reference non-smoker)	1.07 (0.75 - 1.51)	7.10E-01
	Current smoker (reference non-smoker)	0.73 (0.57 - 0.95)	1.67E-02
	Diabetes (reference healthy)	2.62 (2.01 - 3.43)	2.10E-12
	Townsend Index	0.99 (0.95 - 1.04)	6.91E-01

	FLI intermediate vs. low risk	1.26 (0.88 - 1.79)	2.06E-01
	FLI high vs. low risk	2.36 (1.76 - 3.17)	1.00E-08
Extrahepatic Malignancy	Age (years)	1.066(1.065 -1.068)	<1 E-100
	Male (reference Female)	1.17 (1.151 -1.19)	1.69E-77
	Alcohol Intake (units/week)	1.012 (1.009 -1.014)	3.37E-25
	Previous smoker (reference non-smoker)	1.116 (1.082 -1.151)	5.46E-12
	Current smoker (reference non-smoker)	0.874 (0.853 -0.895)	3.58E-28
	Diabetes (reference healthy)	1.025 (0.99 -1.061)	0.16
	Townsend Index	0.978 (0.974 -0.982)	8.95E-26
	FLI intermediate vs. low risk	1.084(1.057-1.113)	1.07E-09
	FLI high vs. low risk	1.119(1.092-1.147)	7.79E-20

S7 – Univariate Cox Regression Analysis for Incident All-cause Mortality

Outcome	Covariate	HR (95% CI)	p-value
All-Cause Mortality	Age (years)	1.107 (1.104 - 1.111)	<1E-100
	Male (reference Female)	1.478 (1.437 - 1.519)	3.13E-167
	Alcohol Intake (units/week)	1.01 (1.006 - 1.013)	2.39E-07
	Previous smoker (reference non-smoker)	1.936 (1.856 - 2.019)	2.45E-206
	Current smoker (reference non-smoker)	0.914 (0.883 - 0.946)	3.35E-07
	Diabetes (reference healthy)	1.997 (1.908 - 2.09)	9.06E-194
	Townsend Index	1.044 (1.037 - 1.05)	8.68E-40
	FLI intermediate vs. low risk	1.42 (1.35-1.50)	1.13E-38
	FLI high vs. low risk	1.96 (1.87-2.06	5.3E-176
	FIB4 intermediate vs. low risk	1.81 (1.74-1.89)	2.5E-176
	FIB4 high risk vs. low risk	3.51 (3.19-3.86)	7.1E-148
	NFS intermediate risk vs. low risk	1.98 (1.90-2.06)	2E-220
	NFS high risk vs. low risk	3.77 (3.38-4.19)	1.4E-131

Table showing hazards ratios (95% CI) for univariate Cox regression outputs in time to event analysis for incident all-cause mortality with all exposure covariates of interest. Categorical variables are analysed as dummy variables.

S8 – Adjusted Model Outputs of Cox Regression for Incident Diseases of Interest

Table output of multivariate cox regression analysis for incident ischaemic heart disease, stroke, hypertension, dyslipidaemia, hepatic malignancy, and extrahepatic malignancy. This is replicate data from the main article's forest plot, but p-values for the regression dummy variable created for FLI risk categories is shown here, with the significance column showing whether level reaches Bonferonni-correction threshold.

	**FLI (Intermediate vs. Low Risk)**			**FLI (High vs. Low Risk)**		
Follow Up Period	HR (95% CI)	p-value	Significance	HR (95% CI)	p-value	Significance
**Ischaemic Heart Disease**						
0-3 years	1.47 (1.32 - 1.63)	2.13E-12	*	2.14 (1.94 - 2.36)	4.78E-52	*
4-6 years	1.38 (1.25 - 1.52)	6.10E-11	*	1.97 (1.80 - 2.15)	1.71E-50	*
7-9 years	1.45 (1.33 - 1.59)	3.40E-16	*	1.80 (1.65 - 1.96)	3.08E-42	*
>9 years	1.31 (1.17 - 1.47)	2.61E-06	*	1.66 (1.49 - 1.85)	1.11E-20	*
**Stroke**						
Entire	1.21 (1.11 - 1.33)	2.55E-05	*	1.31 (1.20 - 1.42)	1.26E-09	*
**Diabetes**						
0-3 years	1.83 (1.59 - 2.10)	1.58E-17	*	4.55 (4.04 - 5.12)	2.81E-139	*
4-6 years	1.36 (1.25 - 1.48)	2.56E-13	*	2.69 (2.50 - 2.88)	6.14E-162	*
7-9 years	2.23 (1.97 - 2.52)	3.19E-36	*	5.69 (5.11 - 6.34)	4.48E-217	*
>9 years	2.76 (2.28 - 3.34)	2.20E-25	*	7.05 (5.95 - 8.34)	3.77E-113	*
**Hypertension**						
0-3 years	1.74 (1.64 - 1.83)	5.54E-87	*	2.84 (2.70 - 2.98)	<1.00E-100	*
4-6 years	1.63 (1.55 - 1.72)	2.59E-71	*	2.61 (2.48 - 2.74)	<1.00E-100	*
7-9 years	1.59 (1.51 - 1.68)	4.98E-64	*	2.42 (2.30 - 2.55)	1.66E-267	*
>9 years	1.48 (1.38 - 1.59)	4.39E-26	*	2.36 (2.21 - 2.53)	1.30E-142	*
**Dyslipidaemia**						
0-3 years	1.70 (1.58 - 1.83)	2.33E-47	*	2.48 (2.32 - 2.64)	2.31E-163	*
4-6 years	1.52 (1.41 - 1.63)	9.41E-31	*	2.09 (1.96 - 2.23)	1.87E-111	*
7-9 years	1.55 (1.44 - 1.67)	2.50E-32	*	2.15 (2.01 - 2.30)	3.26E-114	*
>9 years	1.40 (1.28 - 1.54)	1.34E-12	*	2.05 (1.89 - 2.23)	7.61E-64	*
**Hepatic Malignancy**						
Entire	1.01 (0.70 - 1.45)	0.965429969		1.69 (1.23 - 2.32)	0.001	*
**Extrahepatic Malignancy**						
0-3 years	0.91 (0.86 - 0.97)	0.002	*	0.93 (0.88 - 0.99)	0.014	
4-6 years	1.04 (0.99 - 1.10)	0.146		1.04 (0.98 - 1.10)	0.172	
7-9 years	1.04 (0.98 - 1.10)	0.198		1.10 (1.03 - 1.16)	0.001	*
>9 years	0.94 (0.88 - 1.02)	0.169		1.02 (0.96 - 1.11)	0.4553	

S9 – Cox Regression Analysis of Non-Invasive Fibrosis Score in High Risk FLI Individuals

Non-invasive fibrosis scores		intermediate risk		high risk	
	Follow-up Period	HR (95% CI)	p-value	HR (95% CI)	p-value
FIB4	0-3 Years	1.16 (0.98 - 1.38)	0.08	1.91 (1.37 - 2.66)	0.0002
	3-6 Years	0.94 (0.82 - 1.06)	0.31	1.53 (1.16 - 2.00)	0.0022
	6-9 Years	1.03 (0.92 - 1.16)	0.56	1.42 (1.11 - 1.83)	0.0061
	>9 Years	1.07 (0.94 - 1.22)	0.29	1.40 (1.03 - 1.89)	0.0308
NAFLD Fibrosis Scores	Entire	1.05 (0.98 - 1.13)	0.13	1.28 (1.12 - 1.46)	0.0004

The table shows hazards ratios for multivariate Cox regression models of intermediate and high-risk FIB4 and NAFLD Fibrosis score (reference: low risk) in UKB participants with high-risk FLI. All exposure covariates of interest are included. FIB4 did not meet proportional hazards assumption on regression, thus the risk estimates were modelled at three yearly intervals to allow estimation of time dependent coefficients.

S10- Multivariable Covariate Adjusted Regression Outputs with Components of FLI

Outcome	Ischaemic Heart Disease		Ischaemic Stroke		Type 2 Diabetes Mellitus		Hypertension		Dyslipidaemia		Hepatic Malignancy		Extrahepatic Malignancy		All-cause Mortality	
FLI Component	HR (95%CI)	p	HR (95%CI)	p	HR (95%CI)	p	HR (95%CI)	p	HR (95%CI)	p	HR (95%CI)	p	HR (95%CI)	p	HR (95%CI)	p
BMI	***1.03 (1.01-1.05)***	***0.000146***	0.99 (0.98-1.00)	0.164	***1.06 (1.05-1.07)***	***1.5E-15***	***1.06 (1.05-1.06)***	***3E-56***	1.02 (1.01-1.02)	7.96E-11	1.03 (0.98-1.08)	0.293	0.99 (0.98-1.00)	0.0002	***0.25 (0.19-0.32)***	***6.54E-27***
Waist Circumference (cm)	***1.01 (1.00-1.01)***	***0.005151***	1.01 (1.01-1.02)	2.67E-06	***1.03 (1.02-1.03)***	***4.3E-18***	***1.01 (1.01-1.02)***	***5E-23***	1.01 (1.01-1.01)	9.16E-18	1.00 (0.98-1.03)	0.731	1.01 (1.00-1.01)	4E-08	1.02 (1.02-1.02)	2.075E-29
GGT (U/L)	1.00 (1.00-1.00)	7.44E-13	1.00 (1.00-1.00)	0.009	***1.00 (1.00-1.00)***	***1.9E-12***	***1.00 (1.00-1.00)***	***5E-19***	1.00 (1.00-1.00)	1.1E-177	1.11 (0.99-1.24)	0.066	1.00 (1.00-1.00)	0.0027	***6.41 (4.80-8.56)***	***3.08E-36***
Total Triglyceride (mmol/L)	1.11 (1.09-1.13)	2.25E-37	1.01 (0.98-1.05)	0.576	1.18 (1.16-1.20)	4.09E-87	1.05 (1.04-1.07)	2E-23	***1.19 (1.17-1.20)***	***9.93E-18***	1.01 (1.00-1.01)	5E-38	***0.95 (0.92-0.97)***	***4E-05***	1.00 (0.95-1.05)	0.89

Table showing multivariate Cox regression model outputs for components of FLI (BMI, waist circumference, GGT and total triglyceride levels) and their associated p-value. Bolded and italicised values indicate that a time-dependent coefficient was fitted for the variable and the values indicate the first three years of follow-up from time of FLI calculation. Splines were not fitted in this instance to allow for ease of interpretation of the significance of the FLI component in predicting the comorbid outcome and its general direction of effect. Caution needs to be had with interpreting this due to likely correlation between these components and the magnitude of its risk effect, which is likely to be imprecise.

S11 – Sensitivity Analysis of Listwise Deletion Estimates Accounting for Missing Data in Alcohol Intake

Table below shows hazard ratios for intermediate and high-risk FLI with reference to low-risk FLI derived from multivariable of Cox proportional hazards models fitted without alcohol intake as covariate, for all comorbid outcomes of interest. Alcohol intake has the highest rate of missing and therefore allow assessment of risk estimates effect towards missing data. Models for each time to event outcome used a listwise deletion dataset accounting (not shaded) and not accounting (shaded) for missing data in alcohol intake to examine differences in risk effect estimation.

		intermediate vs. low risk		High vs. low risk		
Time to Event of Interest	Follow up Period	HR (95% CI)	p-value	HR (95% CI)	p-value	n
CVA	**Entire**	1.21 (1.12 - 1.30)	3.863E-07	1.33 (1.25 - 1.43)	1.34E-16	322837
	Entire	1.21 (1.11 - 1.33)	2.105E-05	1.31 (1.20 - 1.43)	6.05E-10	220858
IHD	**0-3 years**	1.44 (1.38 - 1.50)	5.364E-68	2.02 (1.95 - 2.10)	7.67E-307	310714
	**4-6 years**	1.40 (1.34 - 1.47)	1.013E-47	1.94 (1.86 - 2.02)	3.28E-214	
	**7-9 years**	1.40 (1.32 - 1.48)	2.137E-31	1.87 (1.77 - 1.96)	4.45E-123	
	**>9 years**	1.41 (1.29 - 1.54)	1.112E-13	1.79 (1.65 - 1.94)	3.16E-43	
	0-3 years	1.47 (1.32 - 1.64)	1.292E-12	2.16 (1.96 - 2.38)	2.78E-53	213368
	4-6 years	1.39 (1.26 - 1.53)	3.563E-11	1.99 (1.82 - 2.18)	7.70E-52	
	7-9 years	1.46 (1.33 - 1.60)	1.59E-16	1.82 (1.67 - 1.98)	1.41E-43	
	>9 years	1.32 (1.18 - 1.48)	2.163E-06	1.67 (1.50 - 1.86)	5.02E-21	
Hypertension	**0-3 years**	1.75 (1.67 - 1.83)	1.46E-130	2.89 (2.78 - 3.01)	<1.00 E-100	280617
	**4-6 years**	1.70 (1.63 - 1.78)	1.14E-121	2.72 (2.62 - 2.84)	<1.00 E-100	
	**7-9 years**	1.58 (1.52 - 1.66)	2.74E-92	2.46 (2.37 - 2.56)	<1.00 E-100	
	**>9 years**	1.52 (1.43 - 1.61)	4.29E-45	2.41 (2.28 - 2.53)	5.52E-236	
	0-3 years	1.74 (1.65 - 1.84)	4.78E-88	2.86 (2.72 - 3.00)	<1.00 E-100	194089
	4-6 years	1.64 (1.55 - 1.73)	3.02E-72	2.62 (2.50 - 2.76)	<1.00 E-100	
	7-9 years	1.60 (1.51 - 1.68)	6.96E-65	2.44 (2.32 - 2.56)	5.97E-272	
	>9 years	1.49 (1.38 - 1.60)	1.55E-26	2.38 (2.23 - 2.54)	5.28E-145	
Dyslipidaemia	**0-3 years**	1.72 (1.62 - 1.83)	1.26E-72	2.52 (2.39 - 2.65)	4.07E-257	305075
	**4-6 years**	1.58 (1.49 - 1.67)	1.41E-53	2.20 (2.09 - 2.32)	2.29E-192	
	**7-9 years**	1.55 (1.46 - 1.64)	3.54E-46	2.14 (2.03 - 2.26)	2.30E-169	
	**>9 years**	1.47 (1.37 - 1.59)	3.43E-24	2.09 (1.95 - 2.23)	5.23E-105	

	0-3 years	1.71 (1.59 - 1.84)	3.01E-48	2.51 (2.35 - 2.67)	1.36E-167	209402
	4-6 years	1.53 (1.42 - 1.64)	1.77E-31	2.11 (1.98 - 2.25)	7.93E-115	
	7-9 years	1.56 (1.45 - 1.68)	4.44E-33	2.17 (2.03 - 2.32)	1.71E-117	
	>9 years	1.41 (1.28 - 1.55)	5.63E-13	2.07 (1.91 - 2.26)	1.40E-65	
Diabetes	**0-3 years**	1.92 (1.70 - 2.16)	5.11E-27	5.28 (4.77 - 5.83)	5.20E-232	291437
	**4-6 years**	1.31 (1.22 - 1.40)	4.66E-15	2.68 (2.53 - 2.83)	4.92E-256	
	**7-9 years**	2.20 (2.00 - 2.42)	1.46E-59	5.57 (5.13 - 6.05)	<1.00 E-100	
	**>9 years**	2.57 (2.23 - 2.95)	4.33E-40	6.35 (5.62 - 7.18)	1.52E-191	
	0-3 years	1.69 (1.47 - 1.94)	1.18E-13	1.69 (1.47 - 1.94)	2.61E-130	201409
	4-6 years	1.26 (1.16 - 1.37)	5.31E-08	1.26 (1.16 - 1.37)	2.79E-143	
	7-9 years	2.04 (1.80 - 2.31)	5.46E-29	2.04 (1.80 - 2.31)	3.74E-200	
	>9 years	2.52 (2.08 - 3.05)	2.63E-21	2.52 (2.08 - 3.05)	2.76E-105	
Hepatic Malignancy	**Entire**	0.90 (0.67 - 1.21)	0.4829121	1.66 (1.29 - 2.12)	6.30E-05	325483
	Entire	1.01 (0.70 - 1.45)	0.956358	1.70 (1.24 - 2.33)	1.03E-03	222509
Extrahepatic Malignancy	**0-3 years**	0.99 (0.96 - 1.01)	0.2967836	1.01 (0.99 - 1.04)	0.36550	294213
	**4-6 years**	1.01 (0.98 - 1.04)	0.7210924	1.04 (1.02 - 1.07)	0.00236	
	**7-9 years**	1.00 (0.97 - 1.04)	0.8972509	1.07 (1.04 - 1.11)	0.00005	
	**>9 years**	0.97 (0.92 - 1.04)	0.4213545	1.07 (1.00 - 1.13)	0.03258	
	0-3 years	0.91 (0.85 - 0.97)	0.002405	0.93 (0.87 - 0.98)	0.01289	202879
	4-6 years	1.04 (0.99 - 1.10)	0.1477786	1.04 (0.98 - 1.10)	0.18354	
	7-9 years	1.04 (0.98 - 1.10)	0.201177	1.09 (1.04 - 1.15)	0.00106	
	>9 years	0.95 (0.88 - 1.02)	0.1692405	1.03 (0.96 - 1.11)	0.46389	
All-Cause Mortality	**Entire**	0.99 (0.95 - 1.03)	0.636152	1.18 (1.13 - 1.22)	2.05E-16	325517
	Entire	1.01 (0.95 - 1.06)	0.7951523	1.01 (0.95 - 1.06)	6.04E-14	222532

JHEP Reports

CTAT methods

Tables for a "Complete, Transparent, Accurate and Timely account" (CTAT) are now mandatory for all revised submissions. The aim is to enhance the reproducibility of methods.

- Only include the parts relevant to your study
- Refer to the CTAT in the main text as 'Supplementary CTAT Table'
- Do not add subheadings
- Add as many rows as needed to include all information
- Only include one item per row

If the CTAT form is not relevant to your study, please outline the reasons why:

1.1 Antibodies

Name	Citation	Supplier	Cat no.	Clone no.

1.2 Cell lines

Name	Citation	Supplier	Cat no.	Passage no.	Authentication test method

1.3 Organisms

Name	Citation	Supplier	Strain	Sex	Age	Overall n number

1.4 Sequence based reagents

Name	Sequence	Supplier

1.5 **Biological samples**

Description	Source	Identifier

1.6 **Deposited data**

Name of repository	Identifier	Link

1.7 **Software**

Software name	Manufacturer	Version
**RStudio**	**The R Foundation for Statistical Computing**	**4.1.3**

1.8 **Other (*e.g*. drugs, proteins, vectors etc.)**

1.9 **Please provide the details of the corresponding methods author for the manuscript:**

Brian Ho

Wolfson Centre for Personalised Medicine,
Institute of Translational Medicine,
University of Liverpool,
Liverpool, UK

b.ho@liverpool.ac.uk

2.0 Please confirm for randomised controlled trials all versions of the clinical protocol are included in the submission. These will be published online as supplementary information.

